# A theoretical study on toluene oxidization by OH radical

**DOI:** 10.1186/s13065-024-01163-w

**Published:** 2024-04-12

**Authors:** Yumin Mao, Lijuan Yang, Siqi Liu, Yunchang Song, Mengchao Luo, Yongxue Guo

**Affiliations:** 1 Department of Environmental Science and Engineering, North China Electric Power University, Baoding, 071003 Hebei China; 2 Hebei Key Laboratory of Multi-Pollutant Collaborative Control of Flue Gas From Coal-Fired Power Stations, Department of Environmental Science and Engineering, North China Electric Power University, Baoding, 071003 Hebei China

**Keywords:** Quantum chemical calculations, ·OH, Advanced oxidation, Reaction mechanism

## Abstract

Toluene, a prominent member of volatile organic compounds (VOCs), exerts a substantial adverse influence on both human life and the environment. In the context of advanced oxidation processes, the ·OH radical emerges as a highly efficient oxidant, pivotal in the elimination of VOCs. This study employs computational quantum chemistry methods (G4MP2//B3LYP/6-311++G(d,p)) to systematically investigate the degradation of toluene by ·OH radicals in an implicit solvent model, and validates the rationale of choosing a single-reference method using T1 diagnostics. Our results suggest three possible reaction mechanisms for the oxidation of toluene by ·OH: firstly, the phenyl ring undergoes a hydrogen abstraction reaction followed by direct combination with ·OH to form cresol; secondly, ·OH directly adds to the phenyl ring, leading to ring opening; thirdly, oxidation of sidechain to benzoic acid followed by further addition and ring opening. The last two oxidation pathways involve the ring opening of toluene via the addition of ·OH, significantly facilitating the process. Therefore, both pathways are considered feasible for the degradation of toluene. Subsequently, the UV-H_2_O_2_ system was designed to induce the formation of ·OH for toluene degradation and to identify the optimal reaction conditions. It was demonstrated that ·OH and ^1^O_2_ are the primary active species for degrading toluene, with their contribution ranking as ·OH > ^1^O_2_. The intermediates in the mixture solution after reactions were characterized using GC–MS, demonstrating the validity of theoretical predictions. A comparative study of the toluene consumption rate revealed an experimental comprehensive activation energy of 10.33 kJ/mol, which is consistent with the preliminary activation energies obtained via theoretical analysis of these three mechanisms (0.56 kJ/mol to 13.66 kJ/mol), indicating that this theoretical method can provide a theoretical basis for experimental studies on the oxidation of toluene by ·OH.

**Supplementary Information:**

The online version contains supplementary material available at 10.1186/s13065-024-01163-w.

## Introduction

As industrialization rapidly progresses, there’s an increased production and use of a variety of organic materials, with Volatile Organic Compounds (VOCs) being particularly significant [1, 2]. Toluene, a common volatile organic compound, constitutes about 10% of the various VOCs identified in the atmosphere [3]. Its high volatility [4] and pervasive presence in industrial productions and daily life [4–7] contribute to human health risks [8] and induce multiple types of environmental pollutions [9]. This underscores the profound societal significance of exploring efficient removal techniques for these compounds. Traditional VOCs removal techniques primarily target the mitigation of high-concentration VOCs, including toluene, utilizing adsorption, membrane separation, and other recovery methods, as well as degradation methods like combustion [10]. However, as front-end industrial processes continue to advance, the emission of low-concentration VOCs from production pollution sources are increasing. This is compounded by the urgent need to manage low-concentration VOCs resulting from daily activities such as printing [7], indoor coal combustion [11], and automotive activities [12, 13]. Regrettably, traditional techniques designed for high-concentration VOCs, inclusive of toluene, could not efficiently handle low-concentration VOCs [14].

Thermal catalysis and Advanced Oxidation Processes (AOPs, including photocatalysis, Fenton oxidation, electrochemical oxidation, etc.) offer efficient solutions for eliminating low-concentration gaseous and liquid VOCs [15–17]. Thermal catalysis, a conventional method that offers a path to surmount the thermodynamic barrier through heating, enjoys advantages such as economic viability, cost-effectiveness, and minimal secondary pollutant generation [18–20]. Nonetheless, its requirement for elevated temperatures leads to substantial energy consumption, which serves as a key limitation. Consequently, scientists are increasingly leaning towards Advanced Oxidation Processes (AOPs) that offer comparable efficiency but could function at considerably lower reaction temperatures.

Advanced Oxidation Processes (AOPs) are an emerging oxidation technology characterized by the generation of highly active oxidative substances (free radicals), exhibiting versatility in terms of reactants and reaction patterns [21]. Various active species may be present during these processes. For instance, Ohno and co-workers [22] reported the chlorocarboxylation of toluene leveraging ClO_2_· generated from photo-induction of NaClO_2_ under ambient conditions (298 K and 1 atm), yielding 2-chlorobenzoic acid and 4-chlorobenzoic acid as oxidized products. Despite the mild reaction conditions and high reaction rates offered by this active species, the final products still remain pollutant in nature, necessitating further treatment to achieve degradation standards. Additionally, the use of the precursor substance for this active species (NaClO_2_) is not convenient.

Given these factors, this study centers on hydroxyl radicals (·OH) [23], which are typically the workhorses in AOPs, effectuating the actual oxidation process. Hydroxyl radicals exhibit high reactivity and can be generated through a variety of convenient methods. For example, they can be produced from O_2_ and H_2_O on the surface of TiO_2_/Sr_2_Sb_2_O_7_ under photo-excitation conditions [4, 24], or catalytically generated under H_2_O_2_/PMS conditions utilizing MnCo_2_O_4.5_ as the catalyst [25]. Additionally, these radicals can work synergistically with other active species (such as SO_4_·^−^) [25] and are capable of completely mineralizing VOCs into environmentally benign end products, CO_2_ and H_2_O, which require no further treatment [26, 27]. Therefore, increasing attention is being paid to the application of ·OH in the degradation of toluene. Experimental studies aiming at engineering applications mainly focus on regulating factors including the amount of reactants, reaction temperature, humidity, pH, and flow rate to identify optimal conditions for efficient removal of VOCs. Theoretical investigations predominantly intended to elucidate the mechanisms often start with the characterization of intermediate and final products during the reaction process, supplemented by Density Functional Theory (DFT) calculations, with the goal of hypothesizing potential degradation pathways for toluene by ·OH. Therefore, elucidating the multiple possible pathways for the degradation of toluene by ·OH from a theoretical perspective, based on empirical data and species identification during the process, is of considerable value. This is achieved by employing computational quantum chemistry to obtain related thermodynamic data, along with the data from electron density topological and charge analyses. Uncovering the most probable degradation pathway and identifying the rate-determining elementary reaction provide a reliable theoretical underpinning for the further development of AOP techniques deploying ·OH for toluene degradation.

## Calculation and experimental methods

### Calculation methods

This paper uses the Gaussian 09 software package [28] to systematically study the degradation of toluene by ·OH using the B3LYP method at the 6-311++G(d,p) basis set level. The geometric configurations of all species involved in the oxidation pathway were fully optimized under the Implicit Solvent Model (SMD) [29] conditions. The stability of the stable species was confirmed through vibrational frequency analysis, and the reliability of the transition states was verified by Intrinsic Reaction Coordinate (IRC) calculations [30].

The G4MP2 method is grounded in Gaussian-4 theory [31] and integrates approaches like MP2 [32], CCSD(T) [33], and advanced DFT [34], offering reliable predictions for chemical phenomena such as reaction enthalpies, reaction barriers, and ion binding energies [35]. The process begins with geometric optimization and frequency analysis at the B3LYP/6-31G(2d,2p) level [36]. This is followed by MP2 and CCSD(T) calculations across various basis sets [37]. The method also employs an extrapolation technique to determine the basis set limit Hartree–Fock energy (ΔE_HF_), which includes adjustments for spin–orbit corrections (E(SO)) and higher-order corrections (E(HLC)). The final results are obtained using Eq. 1 [31]. Thus, the G4MP2 method offers both accuracy and reduced computation time, making it an effective tool for thermodynamic property prediction [38].

1 $$ E_{0} (G4MP2) =\, E[CCSD(T,FC)/6 - 31G(d)//B3LYP/6 - 31G(2df,p)] + \Delta E_{MP2} + \Delta E_{HF} + E(HLC) + E(ZPE) + \Delta E(SO) $$

Consequently, on the basis of the previously mentioned groundwork, the present study utilizes the G4MP2 thermodynamic composite approach at each stationary point. This is for the purpose of performing calculations with enhanced precision. As a result, the study acquires detailed thermodynamic profiles for all relevant species and each individual reaction within the degradation pathway, all computed at the G4MP2 theoretical level.

To verify the reliablility of the methodology chosen, T1 diagnostics were applied to each species within the reaction system. This diagnostic is an indicator that measures the energy disparity between singlet excited states and the reference state, primarily utilized to determine the multi-reference nature of a wave function. In systems where the T1 diagnostic value is below a certain threshold, single-reference methods like density functional theory can generate accurate results. However, if this threshold is exceeded, indicating a strong multi-reference character, single-reference methods may not be as reliable, necessitating the use of more advanced multi-reference approaches [39]. For closed-shell systems, a T1 diagnostic value below 0.02 is generally considered acceptable [40]. In open-shell systems, this threshold is higher, around 0.045 [41], as seen in species like oxygen-containing compounds and radicals [42].

To test the reliability of single-reference methods in computing this system, a T1 diagnostic was performed on all species involved in the reaction process under the SMD model, using the CCSD/cc-pVDZ combination. The results, as shown in Additional file 1: Table S1, indicate that the T1 values for closed-shell species are all below 0.02, and for open-shell species, all below 0.045, which are within the thresholds for single-reference methods. Thus, the selection of the B3LYP method for structural optimization and the G4MP2 method for thermodynamic data calculation is validated as both reasonable and accurate when assessed through the lens of T1 diagnostics.

### Experimental methods

Nitrogen gas is utilized as a carrier gas. A modest quantity is introduced into a bubbler containing a toluene solution, which entrains the toluene. Nitrogen gas carrying entrained toluene is mixed with another stream of nitrogen gas in a mixing cylinder to dilute the toluene to the desired concentration (90 ppm, 0.6 L/min). This mixed gas is then passed into a reactor filled with a quantified oxidizing agent and fitted with an ultraviolet lamp of 254 nm wavelength. The outlet of the reactor is connected to a gas chromatograph or total hydrocarbon analyzer to measure the concentration of toluene in the exhaust gas. The process is illustrated in Additional file 1: Figure S1.

Toluene concentrations in the gas phase before and after the reaction are assessed via gas chromatography. The formula for calculating the efficiency of toluene removal is as Eq. 2:

2 $$ \eta = \frac{{\left( {c_{inlet} - c_{outlet} } \right)}}{{c_{inlet} }} \times 100\% $$

in the formula; $c_{inlet}$ is the Initial concentration of toluene (mg/m^3^); $c_{outlet}$ is the Toluene concentration after reaction (mg/m^3^); $\eta$ is the oluene Removal Efficiency (%).

The pH of the H_2_O_2_ solution in the bubbler reactor was regulated using H_3_PO_4_ and NaOH. This procedure was implemented to explore the influence of pH on the efficiency of toluene removal via H_2_O_2_ degradation.

DMPO was chosen as the spin-trap reagent to capture the reactive species. A specified volume of DMPO solution was mixed with the sample solution. After mixing, the solution was subjected to ultraviolet light irradiation for a certain duration. The EPR spectra were recorded at room temperature using an Electron Paramagnetic Resonance (EPR) spectrometer to identify the primary free radicals involved in the reaction. Finally, the concentration of volatile organic compounds (VOCs) in the post-reaction solution was determined using purge-and-trap gas chromatography-mass spectrometry (GC–MS).

A specified volume of the post-reaction solution was injected into a purge tube. An inert gas was introduced to purge the VOCs, which were then captured by an adsorbent in a trap tube. The trap tube was heated and back-flushed to desorb the organic compounds, which were then introduced into the gas chromatograph. Following programmed temperature vaporization, the separated compounds were identified by mass spectrometry, with potential organic molecular formulas being determined through online database comparison.

The reaction instruments, models and manufacturers used in the reaction are shown in Additional file 1: Table S2(a) below. The reaction reagent specifications and suppliers used in the reaction are shown in Additional file 1: Table S2(b) below.

## Results and discussion

### Calculation results

Computational quantum chemistry studies has identified three possible mechanisms for the oxidation of toluene by ·OH, which are dehydrogenation on the benzene ring followed by addition reaction (Path 1), direct addition reaction followed by ring-opening (Path 2), and side-chain oxidation followed by ring-opening (Path 3). This study conducts a detailed quantum chemical investigation of aforementioned three possible oxidation mechanisms. The spatial geometric configurations of each species involved in these oxidation pathways were optimized, and their thermodynamic data were obtained via vibrational analysis (as shown in Additional file 1: Figure S2 and Table S3, the Cartesian coordinates for each species are provided in Additional file 1: Table S4. The imaginary frequency data for various transition states and the Intrinsic Reaction Coordinate (IRC) analysis are presented in Additional file 1: Table S5 and Figure S3, respectively). Subsequently, the changes in free energy (∆G_r_) and activation free energy (∆G) for each reaction were calculated. The free energy change and activation free energy obtained using the B3LYP/6-311++G(d,p) combination are denoted as ∆G_r_ʹ and ∆Gʹ, respectively. In this context, reactants, transition states, intermediate products, combined mixtures, and final states are represented by IS, TS, IM, COM, and FS, respectively.

At the beginning of the reaction, we observed that toluene binds to ·OH to form a complex through van der Waals forces, resulting in a decrease in free energy, indicative of an exergonic reaction. Additionally, Intrinsic Reaction Coordinate (IRC) analysis of this initial reaction revealed that the reactions of ·OH with toluene, as well as ·OH with benzoic acid FS8 at all sites both start from the same complex, aligning with the views of Rui Ming Zhang et al. [43]. The free energy data for each reaction is shown in Table 1.

**Table 1:** Comparative analysis of free energy variations pre- and post-reaction and activation free energy values, calculated using B3LYP/6-311++G(d,p) and G4MP2 precision levels

Reaction	Reaction step	∆Gʹ_r_ (kJ/mol)	∆Gʹ (kJ/mol)	∆G_r_ (kJ/mol)	∆G (kJ/mol)
Path1	IS+·OH → IM1 + H_2_O	− 46.80	12.43	− 64.33	28.55
	IS+·OH → IM2 + H_2_O	− 45.85	13.83	− 65.96	31.15
	IS+·OH → IM3 + H_2_O	− 41.93	11.93	− 64.38	29.89
	IM1+·OH → FS1	− 397.08	–	− 408.85	–
	IM2+·OH → FS2	− 397.32	–	− 405.70	–
	IM3+·OH → FS3	− 401.28	–	− 406.93	–
Path2	IS+·OH → IM4	− 40.51	7.96	− 63.82	16.92
	IS+·OH → IM5	− 46.43	0.93	− 60.74	14.37
	IS+·OH → IM6	− 39.29	4.39	− 56.39	17.63
	IS+·OH → IM7	− 44.27	0.17	− 58.95	13.77
	IM4 → FS4	192.82	222.41	216.64	246.52
	IM5 → FS5.1	216.75	223.61	215.58	227.53
	IM5 → FS5.2	187.32	199.05	232.04	242.19
	IM6 → FS6.1	205.76	216.80	226.10	240.04
	IM6 → FS6.2	204.33	214.67	222.79	236.53
	IM7 → FS7	211.42	218.55	227.04	239.30
Path3-1	IS+·OH → IM8 + H_2_O	− 139.78	9.07	− 150.46	5.18
	IM8+·OH → IM9	− 268.74	–	− 295.31	–
	IM9+·OH → IM10.1 + H_2_O	− 174.50	8.92	− 167.12	16.55
	IM9+·OH → IM10.2 + H_2_O	− 85.73	9.27	− 102.18	28.12
	IM10.1+·OH → IM11.1	− 279.88	–	− 306.41	–
	IM10.2+·OH → IM11.2	− 101.44	–	− 117.95	–
	IM11.1 → IM12 + H_2_O	− 49.50	152.32	− 43.18	161.15
	IM11.2 → IM12 + H2O	− 310.29	186.06	− 315.06	189.43
	IM12+·OH → IM13 + H_2_O	− 107.12	–	− 126.61	–
	IM13+·OH → FS8	− 390.01	–	− 395.59	–
Path3-2	FS8+·OH → IM14	− 27.50	10.29	− 43.83	34.85
	FS8+·OH → IM15	− 63.42	8.01	− 61.08	27.16
	FS8+·OH → IM16	− 37.44	11.17	− 47.41	30.53
	FS8+·OH → IM17	− 49.67	10.02	− 56.46	29.93
	IM14 → FS9	202.35	205.10	222.53	233.54
	IM15 → FS10.1	200.70	206.34	231.83	255.92
	IM15 → FS10.2	247.03	244.02	250.94	234.63
	IM16 → FS11.1	210.28	228.05	237.66	247.57
	IM16 → FS11.2	196.15	216.29	213.82	240.08
	IM17 → FS12	208.44	212.50	231.14	241.39

**Fig. 1:**
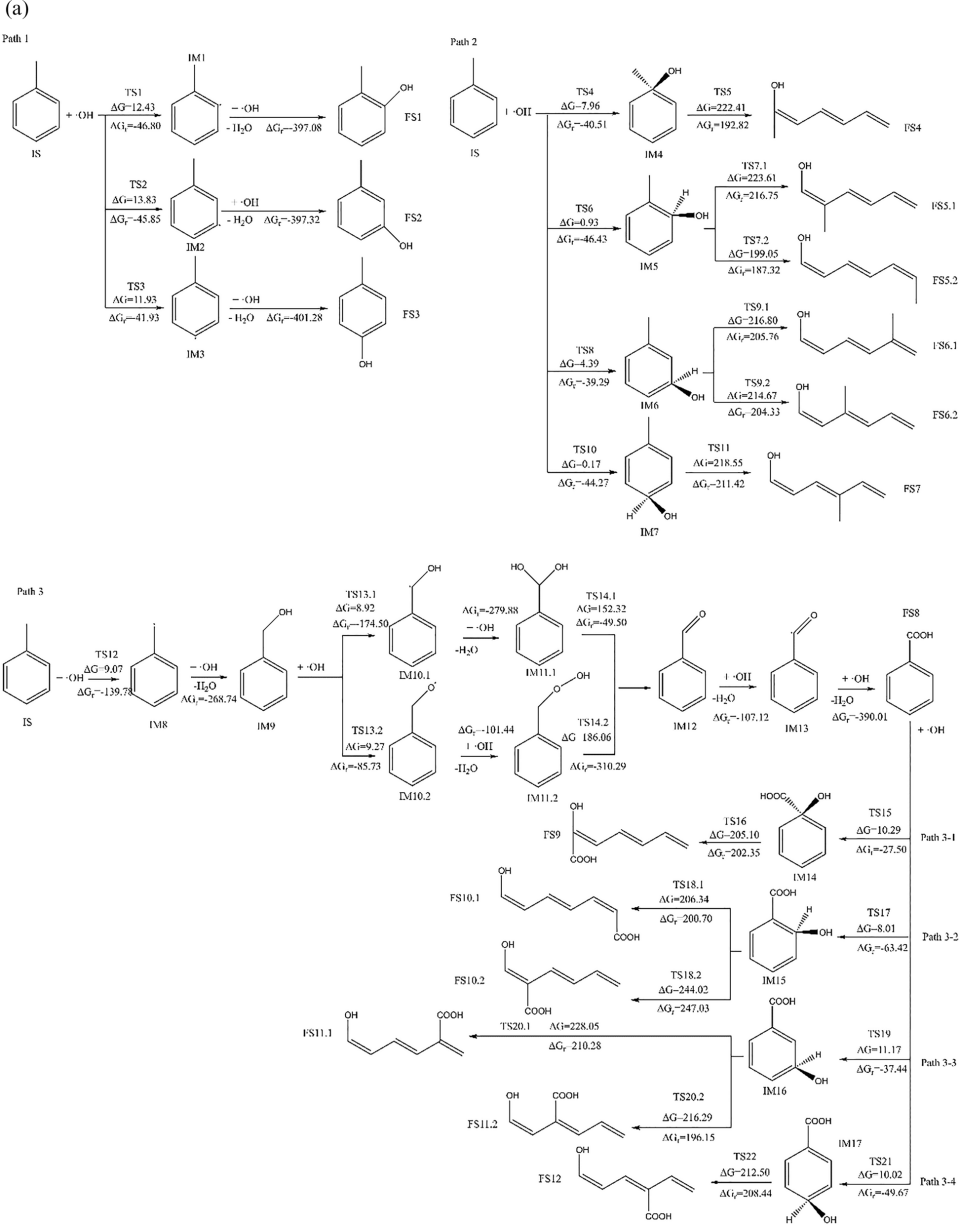

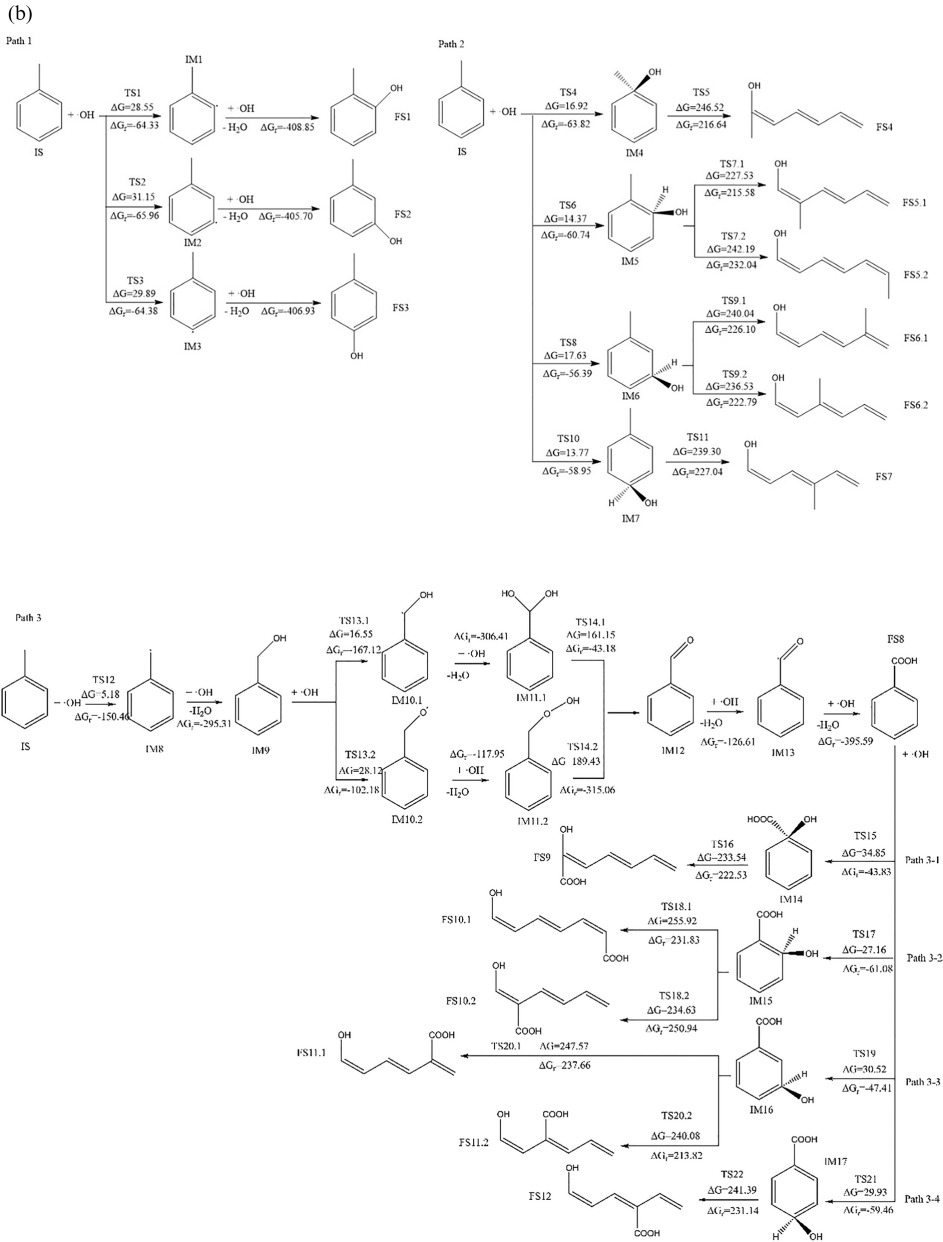

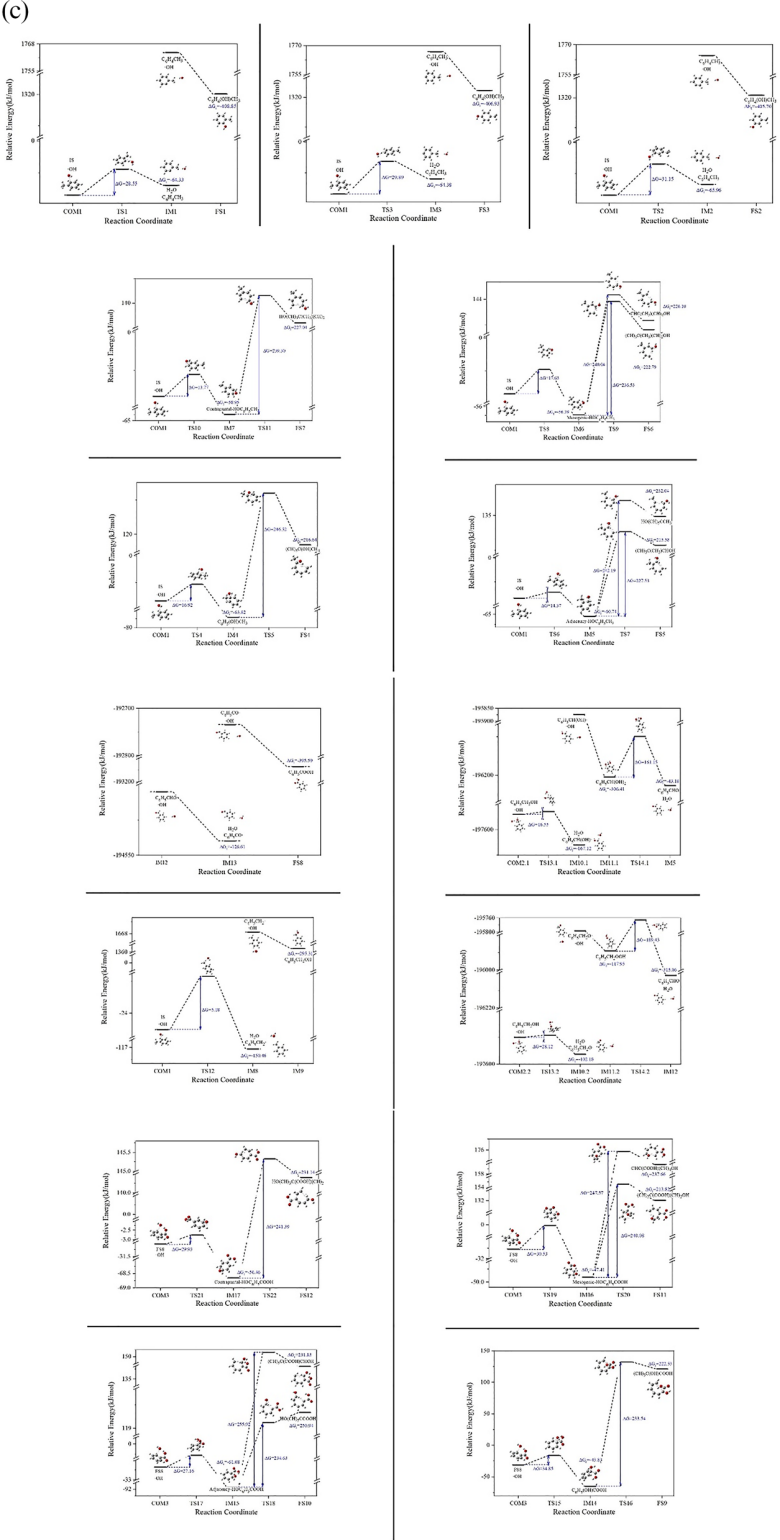
**a** Reaction process diagram at the B3LYP/6-311++G(d,p) level of accuracy. **b** Reaction process diagram at the G4MP2 level of accuracy. **c** The energy profile under G4MP2 precision conditions

When examining the data from two different computational methods in Table 1, we notice that the B3LYP/6-311++G(d,p) method has a relative error of − 12.52 ± 12.37% and − 32.96 ± 37.80% in calculating the free energy changes and activation free energies for this reaction process compared to the G4MP2 method. Despite these discrepancies in error margins, a comparison of Fig. 1 a and b indicates that both methods are consistent in predicting the general trend and direction of the reaction. Based on this observation, the reaction characteristics will be further analyzed using the energy data derived from the more accurate G4MP2 method. The energy profile graph is shown in Fig. 1c.

#### Path 1: Hydrogen abstraction followed by addition reaction

As can be seen from Fig. 1b and c in Path 1, when ·OH approaches toluene, they would interact via weak intermolecular forces to form a complex. After that, ·OH attacks the H atoms located at the 2-, 3-, and 4-positions of the benzene ring (as shown in Fig. 1b). Hydrogen abstraction then occurs via transition states TS1, TS2, and TS3 respectively, generating toluene free radical on the corresponding abstraction sites and H_2_O as the products. The activation free energies for these processes are 28.55 kJ/mol, 31.15 kJ/mol, and 29.89 kJ/mol, respectively. The changes in free energy before and after these reactions are − 64.33 kJ/mol, − 65.96 kJ/mol, and − 64.38 kJ/mol, respectively, indicating that the reactions are energetically favorable. Additionally, due to the low activation free energies, this hydrogen abstraction reaction is likely to occur readily. The generated toluene-based free radical fragments [44] are highly reactive and will rapidly react with ·OH, directly yielding benzyl alcohol, which is consistent to the results described by Sourab Sinha and others [45], the changes in free energy before and after the reaction are − 408.85 kJ/mol, − 405.70 kJ/mol, and − 406.93 kJ/mol, respectively.

#### Path 2: Addition reaction followed by ring opening

**Fig. 2:**
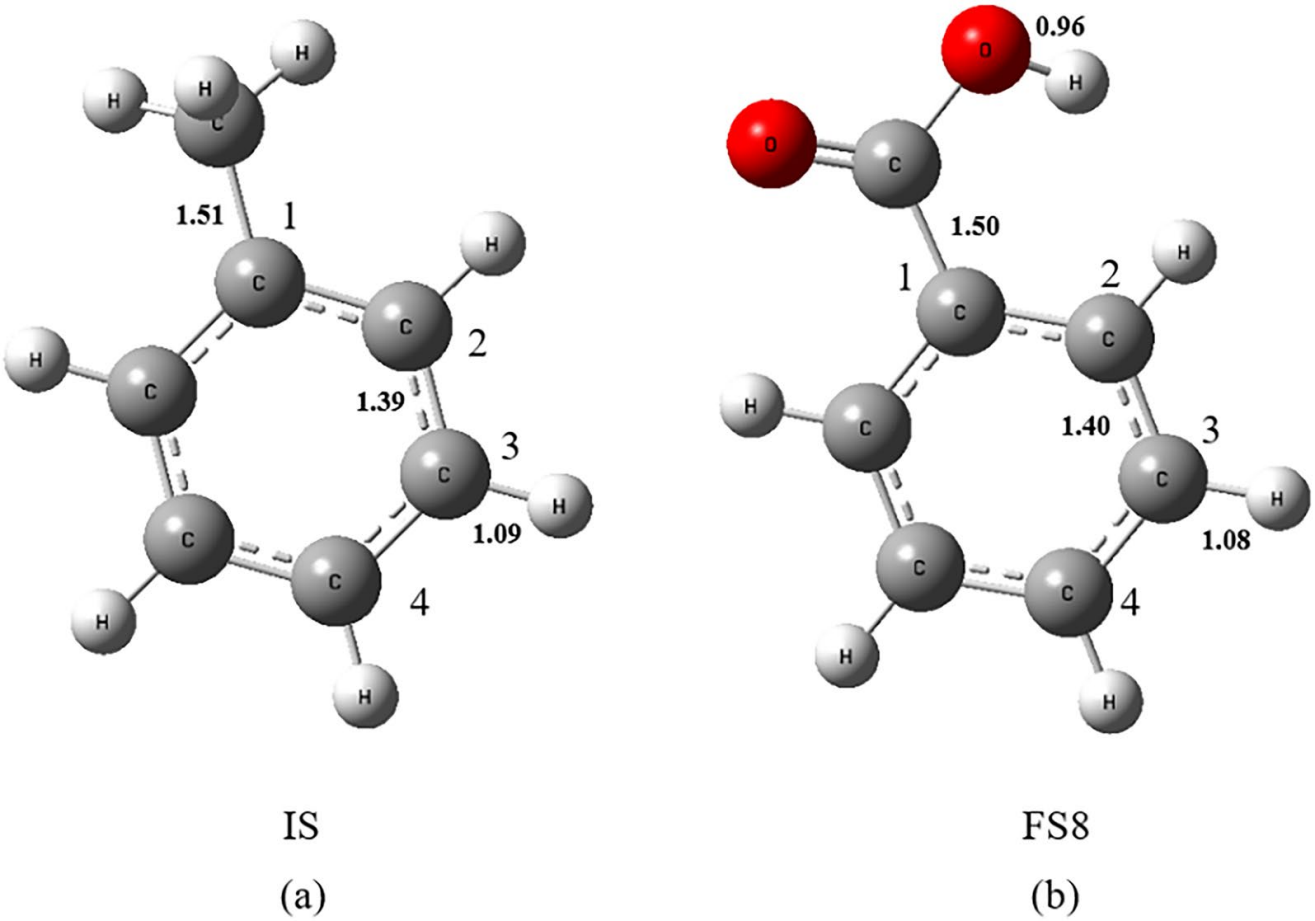
Structural Diagrams of Toluene (IS) and Benzoic Acid (FS8) with Attack Sites

As can be seen from Fig. 1 b and c in Path 2, ·OH attacks the C1, C2, C3, and C4 positions of the benzene ring (as shown in Fig. 2a), and products IM4, IM5, IM6, and IM7 were generated via transition states TS4, TS6, TS8, and TS10, respectively. For the four examined reactions, the measured activation free energies are respectively 16.92 kJ/mol, 14.37 kJ/mol, 17.63 kJ/mol, and 13.77 kJ/mol. Correspondingly, the free energy changes are − 63.82 kJ/mol, − 60.74 kJ/mol, − 56.39 kJ/mol, and − 58.95 kJ/mol. These values collectively suggest that the addition reactions involving ·OH at these four specific carbon positions proceed spontaneously. In products IM4, IM5, IM6 and IM7, our data revealed that after the hydroxyl addition, the bond length between the attacked carbon atom and its neighboring carbon atom increases from 1.39 Å to roughly 1.50 Å/1.51 Å, indicating that the C–C bond is weakened. Theoretically, opening the weakened C–C bond should be easier than the original benzene ring's C–C bond. Depending on the structural differences of IM4, IM5, IM6, IM7, there could be either one or two pathways to cleavage the C–C bond. Among them, IM4 and IM7 only have one ring-opening pathway: In IM4, via transition state TS5, the C1–C2 bond is broken, generating product FS4; in IM7, via transition state TS11, the C3–C4 bond is broken, generating product FS7. The activated carbon–carbon bond breakage in IM5 and IM6 has two mechanisms to open the ring: IM5 can go through transition state TS7.1 for C2–C3 bond breaking ring-opening reaction, generating product FS5.1, or it can go through transition state TS7.2 for C1–C2 bond breaking ring-opening reaction, generating product FS5.2; IM7 may go through transition state TS9.1, for C2–C3 bond breaking ring-opening reaction, generating product FS6.1, or it may go through transition state TS9.2 for C3–C4 bond breaking ring-opening reaction generating product FS6.2. As can be seen from Fig. 1b, in the aforementioned ring-opening reactions, the reaction via transition state TS7.1 has the lowest activation free energy, with an activation free energy of 227.53 kJ/mol and a free energy change of 215.58 kJ/mol. The reaction via transition state TS5 has the highest activation free energy, with an activation free energy of 246.52 kJ/mol and a free energy change of 216.6 kJ/mol. This suggests that the hydroxyl addition has already weakened the C–C bond strength, making the ring-opening reactions easier to occur, with the reaction via transition state TS7.1 being relatively more facile.

#### Path 3: Side chain oxidation followed by ring opening

As seen in Fig. 1 b and c, ·OH attacks the hydrogen on the side chain (–CH_3_) of toluene and proceeds via the transition state TS12 to undergo a hydrogen abstraction reaction, forming a benzyl free radical (IM8) and H_2_O. The reaction’s activation free energy is recorded at 5.18 kJ/mol, accompanied by a substantial free energy change of − 150.46 kJ/mol. This significant negative change in free energy and the low activation energy strongly suggest that the reaction, specifically the hydrogen abstraction process, is spontaneous and also occurs with ease. Following this, IM8 rapidly reacts with ·OH to generate benzyl alcohol (IM9), the change in free energy for this reaction is − 295.31 kJ/mol. Studies indicate that ·OH's further oxidation of benzyl alcohol (IM9) can proceed via two pathways: one of them is that ·OH continues to attack the hydrogen on the methyl group to undergo a further hydrogen abstraction reaction, proceeding via transition state TS13.1 to generate IM10.1; the other is that ·OH attacks the hydrogen of the hydroxyl group, proceeds via transition state TS13.2 to undergo a hydrogen abstraction reaction, and generate IM10.2. For the hydrogen abstraction reactions, the activation free energies determined by the two methods are 16.55 kJ/mol and 28.12 kJ/mol. The corresponding changes in free energy are − 167.12 kJ/mol and − 102.18 kJ/mol, respectively. These values indicate that the reactions are inclined to occur without significant barriers. Radicals IM10.1 and IM10.2 continue to react swiftly with ·OH, directly forming the addition products IM11.1 and IM11.2 with changes in free energy of − 306.41 kJ/mol and − 117.95 kJ/mol, respectively. Subsequently, these products undergo dehydration reactions via TS14.1 and TS14.2 to form benzaldehyde (IM12). The activation free energies for these dehydration reactions are 161.15 kJ/mol and 189.43 kJ/mol, respectively, with changes in free energy of − 43.18 kJ/mol and − 315.06 kJ/mol. This indicates that the dehydration reaction of IM11.1 is more facile than that of IM11.2. Furthermore, this dehydration step is the rate-determining step in the transformation from IM9 to IM12, suggesting that the pathway where ·OH attacks the methyl group of benzyl alcohol (IM9) to form benzaldehyde is favorable. Following this, ·OH continues to attack the hydrogen on the aldehyde group of benzaldehyde (IM12), which then undergoes a hydrogen abstraction reaction to generate the free radical IM13. IM13 combines with ·OH to generate benzoic acid (FS8). In terms of intermediate products and reaction processes, this reaction pattern is similar with the results reported in Sr_1−x_Ba_x_TiO_3_ surface [46], UV/TiO_2_ [47], and Mn/UIO-66/H_2_O_2_ [48] systems.

·OH can continue to add onto the phenyl ring of benzoic acid (FS8), subsequently triggering ring-opening reactions. Similar to Path 2, as depicted in Figs. 1a, c, 2b, ·OH targets positions C1, C2, C3, and C4 on the benzoic acid phenyl ring, leading to addition reactions via transition states TS15, TS17, TS19, and TS21. The resulting products are IM14, IM15, IM16, and IM17, respectively. The activation free energies for the four reactions are 34.85 kJ/mol, 27.16 kJ/mol, 30.53 kJ/mol, and 29.93 kJ/mol, with changes in free energy of − 43.83 kJ/mol, − 61.08 kJ/mol, − 47.41 kJ/mol, and − 56.46 kJ/mol, respectively, suggesting the thermodynamic feasibility of addition reactions at these carbon atom positions, with the addition at C1 being the most likely to occur.

In products IM14, IM15, IM16, and IM17, the bond length between the carbon atom, which has been attacked by the hydroxyl group, and its adjacent carbon atom increases from 1.40 Å to approximately 1.51 Å. This lengthening indicates that both hydroxyl addition to benzoic acid and toluene elongate the C–C bond to approximately 1.51 Å, theoretically weakening the C–C bond to a similar extent. Thus, it can be speculated that the reaction barriers of ring-opening after radical addition should be quite similar for both benzoic acid and toluene.

Similar to Path 2, IM14 and IM17 each have a single ring-opening mechanism. IM14 undergoes C1–C2 bond cleavage via transition state TS16, generating product FS9. IM17 undergoes C3–C4 bond cleavage via transition state TS22, generating product FS12. Both IM15 and IM16 have two ring-opening pathways: one involves C1–C2 bond cleavage in IM15 via transition state TS18.1, generating product FS10.1, and the other involves C2–C3 bond cleavage via transition state TS18.2, yielding product FS10.2. Similarly, IM16 can undergo C2–C3 bond cleavage via transition state TS20.1 to generate product FS11.1, or it can undergo C3–C4 bond cleavage via transition state TS20.2 to yield product FS11.2.

Figures 1b and c reveal that among the ring-opening reactions analyzed, the pathway involving transition state TS16 has the lowest activation free energy, measured at 233.54 kJ/mol, with a corresponding free energy change of 222.53 kJ/mol. The pathways via transition states TS20.1, TS20.2, and TS22 show activation free energies of 247.57 kJ/mol, 240.08 kJ/mol, and 241.39 kJ/mol, respectively. This highlights the close similarity between the reaction barriers for ring-opening after hydroxyl addition to benzoic acid and hydroxyl addition to toluene. Although the ring-opening reaction is the most challenging step in both Path 2 and Path 3, the reaction barriers are all around 240 kJ/mol, significantly lower than the approximately 350 kJ/mol needed to directly open the phenyl ring C–C bond. Hence, it can be inferred that the pathway for ·OH to degrade toluene via Path 2 and Path 3 is indeed viable due to the greatly reduced barrier of ring-opening reaction after hydroxyl radical addition.

### Evaluation of toluene removal performance

**Fig. 3:**
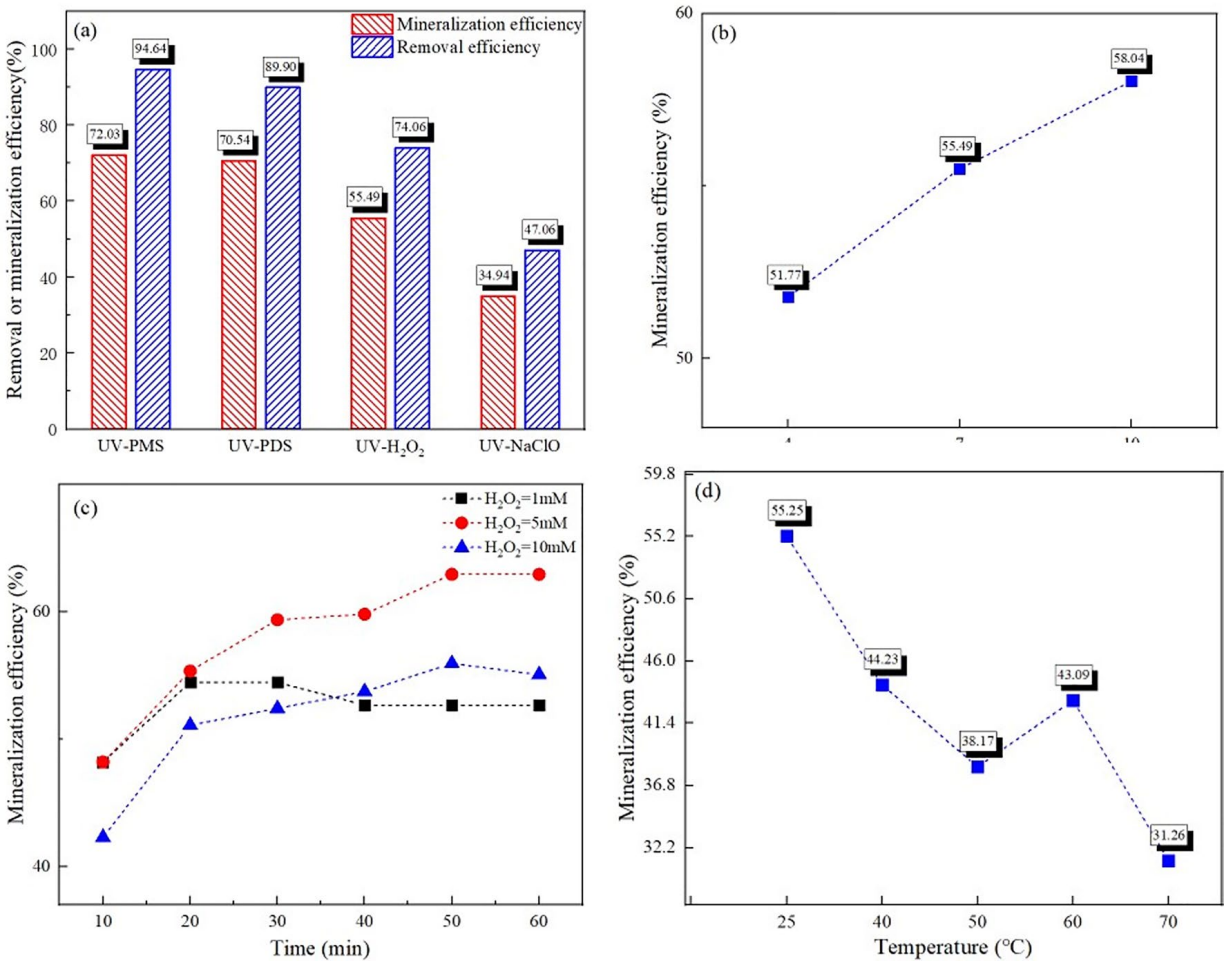
Performance of typical UV-AOP systems on the removal and mineralization of toluene (**a**). Effect of the H_2_O_2_ solution pH (**b**), the H_2_O_2_ concentration (**c**) and the reaction temperature (**d**) on the toluene removal

To validate the preceding computational results and investigate the efficacy of ·OH in degrading toluene experimentally, we utilized UV irradiation to stimulate H_2_O_2_ for ·OH generation. As shown in Fig. 3a, through an integrative comparison of the mineralization and removal efficiency of toluene across different systems (wherein the oxidizing agent was dosed at 10 mM in each system), it was observed that the toluene removal effectiveness ranked as follows: UV-PMS>UV-PDS>UV-H_2_O_2_>UV-NaClO. In particular, UV-PMS achieved a toluene removal rate of 94.64% and a mineralization rate of 73.02%. Compared to the UV-PMS system, the UV-PDS system displayed a comparable mineralization efficiency for toluene, though its removal rate was slightly lower, at 89.90%. The toluene removal efficiency of UV-H_2_O_2_ was 74.06%, with a mineralization efficiency of 55.49%. The UV-NaClO system exhibited the poorest performance in terms of toluene removal, with a mineralization efficiency and removal efficiency of 34.94% and 47.06% respectively. Considering cost-effectiveness, the industrial prices of PMS and PDS are 3–5 times that of H_2_O_2_. Consequently, H_2_O_2_ offers superior cost-effectiveness and is thus more suitable for industrial applications and technological promotion.

To determine the optimal experimental conditions for the UV-H_2_O_2_ mineralization of toluene, we further investigated the influence of some key parameters, including solution pH, H_2_O_2_ concentration, and reaction temperature. Given that the pH of the H_2_O_2_ solution determines the existing form of H_2_O_2_ and the formation of free radicals, we first investigated the influence of the pH of H_2_O_2_ solution on toluene degradation (the H_2_O_2_ dosage is 10 mM). As depicted in Fig. 3b, with the increase of pH in the H_2_O_2_ solution, the mineralization efficiency of toluene improved accordingly, increasing from 52.94% at pH = 4 to 56.63%. This could be attributed to alkaline conditions fostering the decomposition of H_2_O_2_ into HO_2_^−^, which is more readily photolyzed into ·OH under UV light compared to H_2_O_2_, as indicated by Eqs. (3–4). Additionally, according to literature, acidic conditions impede ·OH production [49], thus at pH 4, the yield of ·OH from the photolytic reaction of H_2_O_2_ may diminish, resulting in a decline in toluene mineralization efficiency. The positive correlation of ·OH concentration with toluene mineralization efficiency observed from these experiments led us to further study the influence of H_2_O_2_ concentration (i.e., ·OH precursor concentration) on toluene’s mineralization efficiency. As shown in Fig. 3c, when the pH is the origin, with the increment of H_2_O_2_ concentration from 1 to 5 mM, the mineralization efficiency of toluene initially rose, then fell, peaking at a H_2_O_2_ concentration of 5 mM with a toluene mineralization rate of 62.99%. The impact of temperature on toluene mineralization efficiency was further examined, as shown in Fig. 3d. When the H_2_O_2_ dosage is 10 mM and the pH is the origin, as temperature rose from 25 to 50 °C, the mineralization efficiency of toluene in the UV-H_2_O_2_ system gradually decreased. Although at 60 °C, toluene mineralization efficiency increased to 43.09%, it subsequently dropped to 31.26% at 70 °C. This pattern could be attributed to the fact that higher temperatures reduce toluene’s solubility in water while simultaneously increasing the system’s gas–liquid mass transfer resistance, thus hampering the reaction. Therefore, the overall mineralization efficiency of UV-H_2_O_2_ on toluene demonstrates a decreasing trend. However, between 50 and 60 °C, the mineralization efficiency of UV-H_2_O_2_ on toluene showed a temporary uptick, due to heat activation promoting free radical generation and accelerating toluene oxidative degradation. But between 60 and 70 °C, the inhibitory effect of temperature on gas–liquid mass transfer surpassed its activating effect, resulting in a continuing decline in toluene's mineralization efficiency.

3 $$ {\text{H}}_{{2}} {\text{O}}_{{2}} \leftrightarrow {\text{HO}}_{{2}}^{ - } + {\text{H}}^{ + } $$

4 $$ {\text{HO}}_{{2}}^{ - } + {\text{hv}} \to \cdot {\text{OH}} + \cdot {\text{O}}^{ - } $$

### Determination of free radical species and reaction mechanism

**Fig. 4:**
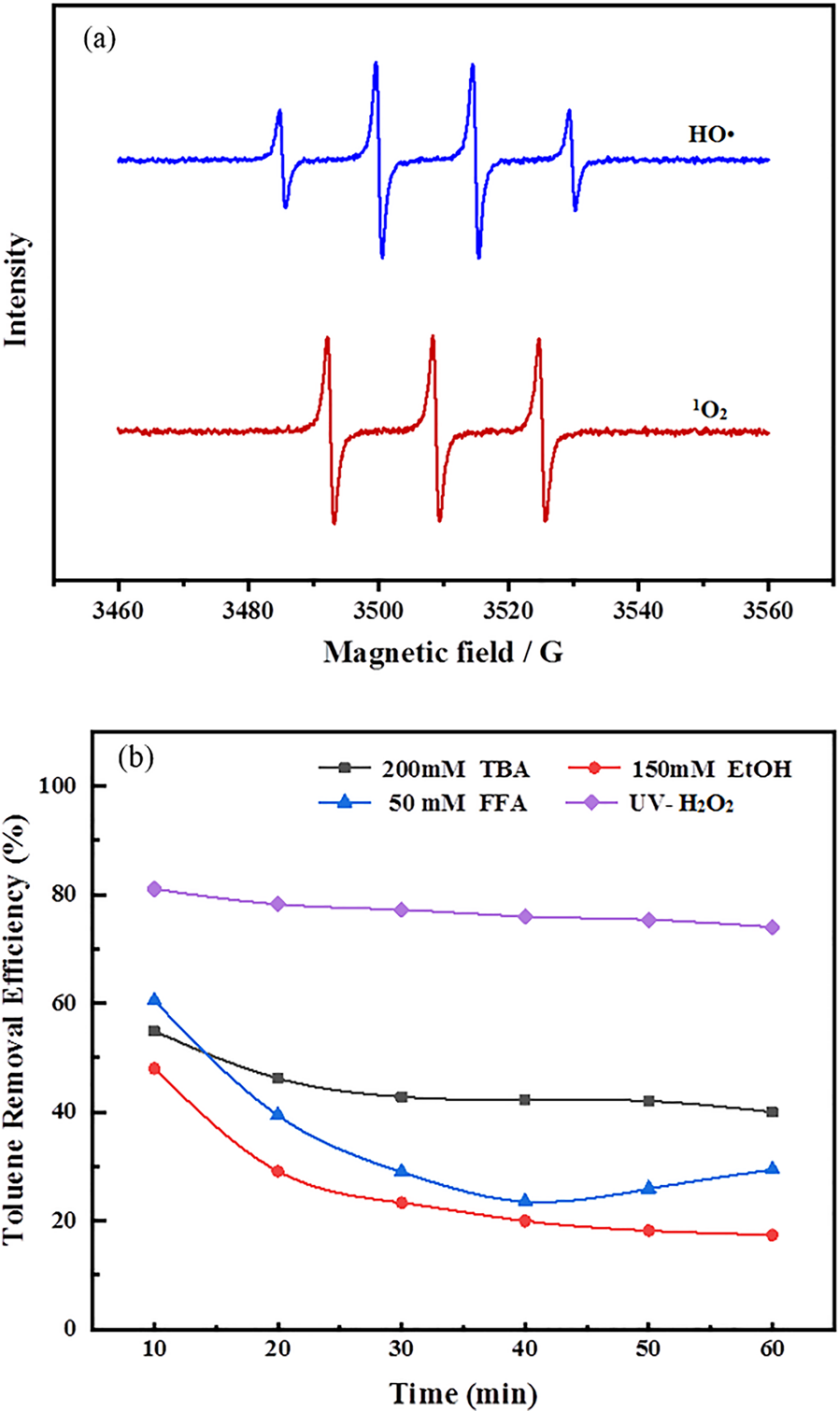
EPR analysis of UV-H_2_O_2_ system (**a**). Radical quenching tests (**b**)

In order to explore the reaction pathways and mechanism of mineralization of toluene under UV-H_2_O_2_ conditions, we first performed an analysis on the free radicals in the UV-H_2_O_2_ system using Electron Paramagnetic Resonance (EPR). As shown in the EPR spectrum in Fig. 4a, both ·OH and ^1^O_2_ show strong signals, indicating that the major active free radical species in the UV-H_2_O_2_ system are ·OH and ^1^O_2_, which probably play a leading role in the degradation of toluene. Next, we employed a free radical quenching experiment to quantify the contributions of ·OH and ^1^O_2_ in the UV-H_2_O_2_ system. Table 2 lists the most common quenchers for ·OH and ^1^O_2_, which are tert-Butyl alcohol (TBA), ethanol (EtOH), and furfuryl alcohol (FFA). Their reaction rate constants with ·OH and ^1^O_2_ are also detailed in the table. The results of the free radical quenching experiment, as shown in Fig. 4b, revealed that after the addition of TBA, the removal rate of toluene dropped from 74.05 to 39.99%. When EtOH was added, the removal rate of toluene dropped to 17.34%. Considering that TBA is a quencher for ·OH, and EtOH is capable of quenching both active species, it is apparent that ·OH contributed more to the degradation of toluene in the UV-H_2_O_2_ system. The addition of FFA caused the degradation efficiency of toluene to decrease to 29.40%, suggesting that ^1^O_2_ also takes part in the UV-H_2_O_2_ degradation reaction of toluene and makes a significant contribution. In conclusion, based on the free radical quenching experiment and EPR characterization, ·OH is the primary active species for the degradation of toluene by UV-H_2_O_2_, while ^1^O_2_ is the secondary one. The aforementioned experimental results and analyses solidly supports the feasibility and rationality of theoretical calculations. Nevertheless, further investigation is required to elucidate the reaction pathways for the degradation of toluene induced by ^1^O_2_.

**Table 2:** Reaction rate constant of quenching agent and free radical reaction

Quenching agent	Radical	Reaction rate constant
Tert-butanol (TBA)	·OH	k = 3.8–7.6 × 10^8^ M^−1^S^−1^
Ethyl alcohol (EtOH)	·OH	k = 1.9 × 10^9^ M^−1^S^−1^
Furfuryl alcohol (FFA)	^1^O_2_	k = 1.2 × 10^8^ M^−1^S^−1^
	·OH	k = 1.5 × 10^10^ M^−1^S^−1^
	·OH	k = 6.6 × 10^9^ M^−1^S^−1^

**Fig. 5:**
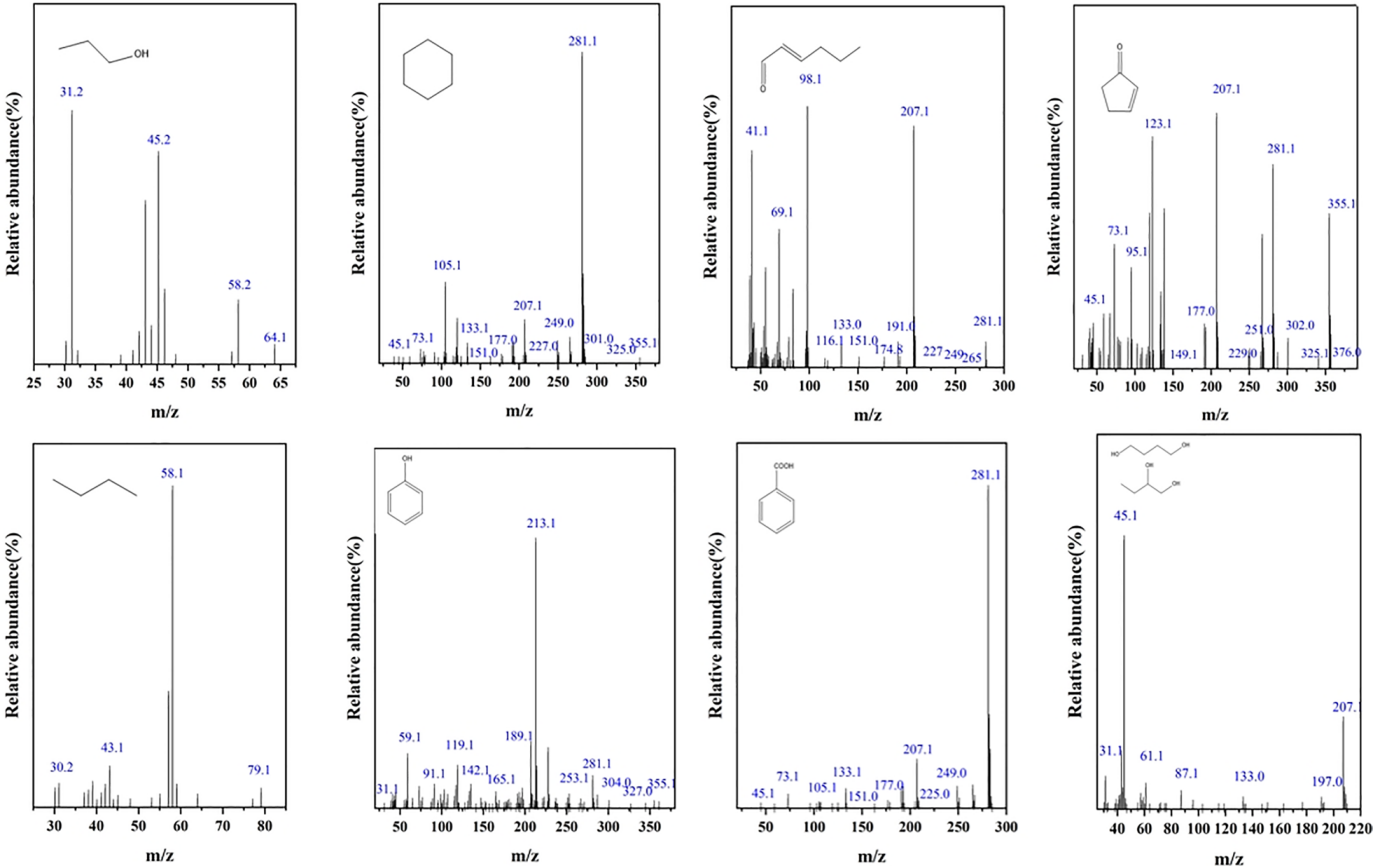
GC–MS analyses on key intermediates generated in toluene degradation by UV-H_2_O_2_ system

In order to elucidate the degradation pathways of toluene by ·OH and confirm the key intermediates predicted by theoretical calculations, GC–MS analysis was performed on the solution following UV-H_2_O_2_ induced degradation of toluene. The gas chromatography data presented in Fig. 5 identified various compounds including ethanol, propane, 2-pentenal, benzoic acid, 2-cyclopenten-1-one, cyclohexane, along with other alcohols, aldehydes, ketones, and both aromatic and aliphatic hydrocarbons in the post-reaction liquid phase. The identification of benzoic acid (FS8) aligns with the predicted key product, supporting the theoretical analysis. Additionally, the detection of chain alcohols and aldehydes in the mixture indicates that ring-opening and further degradation of toluene occurred.

### Investigation into the reaction rate constants

Building on the aforementioned calculations and analyses, an in-depth kinetic study of the reaction was conducted. The initial investigation involved examining the molecular dynamics of the experiment. It is widely recognized that the reaction between hydrogen peroxide and toluene in a liquid-phase reactor follows first-order kinetics [50]. In light of this, using Eq. 5, the reaction rate constants at different temperatures were calculated by fitting the data (as illustrated in Additional file 1: Figure S4). Following this, the Arrhenius expression for these temperature ranges was ascertained through additional data fitting (as illustrated in Additional file 1: Figure S5), leading to the experimentally determined activation energy. The results of this process are presented in Table 3.

5 $$ \ln \frac{{c_{t} }}{{c_{0} }} = - kt $$

where t is the Time(s); C_t_ is the The concentration of toluene at time (mg/m^3^); C_0_ is the The initial concentration of toluene(mg/m^3^); k is the rate constant for the reaction(s^−1^).

**Table 3:** Experimentally determined reaction rate constants, arrhenius expressions, and activation energies

T(K)	298	313	323	333	343
Lnk	− 8.61	− 8.23	− 8.18	− 8.10	− 8.04
Arrenius expression (s^−1^)	k = 1.27 × 10^−2^exp (− 1241.89/T)				
E_a_ (kJ/mol)	10.33				

Next, we engaged in a direct dynamics study based on theoretical calculations. Mirroring the experimental approach, which measured the rate of toluene consumption, the theoretical segment focused on the initial step reactions of each pathway. Activation free energies for these initial reactions across a range of temperatures (303–340 K) were calculated using the Shermo software package [51] (as illustrated in Additional file 1: Table S6). Following this, liquid-phase reaction rate constants were determined using Transition State Theory (TST) [52]. The next step involved fitting the Arrhenus expressions for each reaction within this temperature range, as shown in Additional file 1: Figure S6, which facilitated the computation of activation energies for each reaction. These findings are summaried in Tables 4 and 5. In addressing the initial steps’ varying selectivities, reactions with the highest and lowest activation free energies were selected for the calculations, with the combined data for this step expected to span these extremes.

**Table 4:** Initial steps of each reaction pathway, their activation free energies, and reaction rate constants at various temperatures

T	1000/T	k(s^−1^ M^−1^)						
		Path1		Path2		Path3-1	Path3-2	
		IS+·OH → IM1 + H_2_O	IS+·OH → IM2 + H_2_O	IS+·OH → IM6	IS+·OH → IM7	IS+·OH → IM8 + H_2_O	FS8+·OH → IM14	FS8+·OH → IM15
303	3.30	9.17 × 10^10^	2.66 × 10^10^	1.29 × 10^12^	6.56 × 10^12^	1.07 × 10^11^	5.32 × 10^11^	9.10 × 10^11^
305	3.28	9.43 × 10^10^	2.75 × 10^10^	1.30 × 10^12^	6.61 × 10^12^	1.10 × 10^11^	5.32 × 10^11^	9.16 × 10^11^
310	3.23	1.01 × 10^11^	3.00 × 10^10^	1.32 × 10^12^	6.66 × 10^12^	1.16 × 10^11^	5.34 × 10^11^	9.30 × 10^11^
315	3.17	1.08 × 10^11^	3.26 × 10^10^	1.35 × 10^12^	6.71 × 10^12^	1.23 × 10^11^	5.36 × 10^11^	9.42 × 10^11^
320	3.13	1.15 × 10^11^	3.54 × 10^10^	1.37 × 10^12^	6.77 × 10^12^	1.30 × 10^11^	5.38 × 10^11^	9.56 × 10^11^
325	3.08	1.22 × 10^11^	3.83 × 10^10^	1.39 × 10^12^	6.83 × 10^12^	1.37 × 10^11^	5.39 × 10^11^	9.70 × 10^11^
330	3.03	1.30 × 10^11^	4.14 × 10^10^	1.41 × 10^12^	6.89 × 10^12^	1.44 × 10^11^	5.41 × 10^11^	9.83 × 10^11^
335	2.99	1.38 × 10^11^	4.45 × 10^10^	1.44 × 10^12^	6.95 × 10^12^	1.51 × 10^11^	5.43 × 10^11^	9.96 × 10^11^
340	2.94	1.46 × 10^11^	4.79 × 10^10^	1.46 × 10^12^	6.98 × 10^12^	1.59 × 10^11^	5.45 × 10^11^	1.01 × 10^12^

**Table 5:** Arrhenius expressions and activation energies (E_a_) for each initial step reaction in the 303–340 K temperature range

Reaction	Rate determining step	E_a_ (kJ/mol)	Arrenius expression (s^−1^ M^−1^)
Path1	IS+·OH → IM1 + H_2_O	10.80	k = 6.66 × 10^12^ exp (− 1298.59/T)
	IS+·OH → IM2 + H_2_O	13.66	k = 6.00 × 10^12^ exp (− 1642.61/T)
Path2	IS+·OH → IM6	2.79	k = 3.91 × 10^13^ exp (− 335.47/T)
	IS+·OH → IM7	1.42	k = 1.15 × 10^13^ exp (− 170.71/T)
Path3-1	IS+·OH → IM8 + H_2_O	9.14	k = 4.03 × 10^12^ exp (− 1099.27/T)
Path3-2	FS8+·OH → IM14	0.56	k = 6.65 × 10^11^ exp (− 67.73/T)
	FS8+·OH → IM15	2.38	k = 2.34 × 10^12^ exp (− 286.01/T)

Table 5 shows that the activation energies for the initial reactions of the four pathways range from 0.56 to 13.66 kJ/mol. These notably low activation energies promote the progress of the subsequent reactions, implying that each of the four pathways is viable for further reaction. Meanwhile, Table 3 presents an experimentally determined composite activation energy of 10.33 kJ/mol. This correspondence underscores the effectiveness of the theoretical kinetic analysis in precisely predicting reaction rates observed under experimental conditions.

**Fig. 6:**
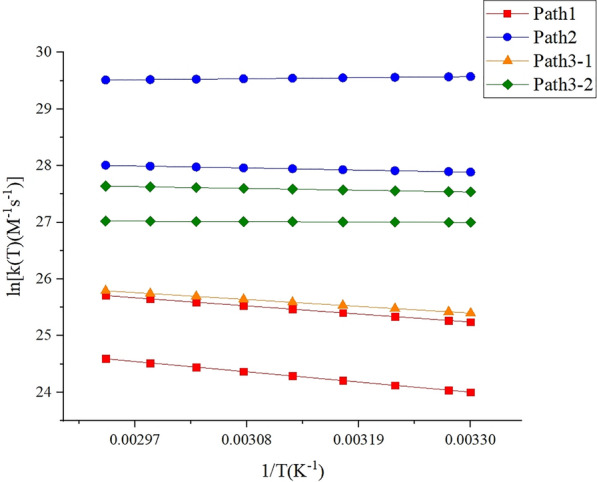
Variation of Reaction rate constants for the initial steps of each reaction in the 303–340 K temperature range

To explore the impact of temperature on reaction rate constants, data from Table 4 are plotted in Fig. 6. This visualization clearly shows that within the temperature range of 303–340 K, the initial reaction rate constants for the four pathways do not exhibit substantial temperature dependency. However, there is a noticeable temperature sensitivity observed for Path1 and Path3-1, in contrast to the consistently maintained reaction rates of Path2 and Path3-2 across the same temperature range.

## Conclusions

This study utilizes Gaussian 09 with the G4MP2//B3LYP/6-311++G(d,p) approach for an in-depth theoretical analysis of the ·OH oxidation of toluene, incorporating the implicit solvent model (SMD) conditions. Complementing this, we integrate experimental research, radical detection, analytical product testing, and kinetic studies to comprehensively examine the UV-H_2_O_2_ degradation mechanism of toluene. Our investigation spans both computational simulations and experimental observations. Key findings are summarized as follows:

1. T1 diagnostics validate the suitability of single-reference methods for the system involving ·OH oxidation of toluene. When comparing the energy values derived from the single-reference B3LYP/6-311G++(d,p) method with those from the G4MP2 method, some variations are observed. However, both methods consistently align in their predictions of reaction mechanisms and trends in energy changes.
2. The ·OH oxidation of toluene primarily follows three mechanisms: dehydrogenation followed by addition to the benzene ring forming phenol, direct addition of ·OH to the benzene ring followed by ring-opening, and side-chain oxidation by ·OH forming benzoic acid followed by ring-opening. Both of the latter mechanisms involve ring-opening of toluene via ·OH addition, with reaction barriers around 240 kJ/mol, significantly lower than the bond energy of the benzene ring’s C–C bond. This indicates that ·OH addition significantly eases ring-opening, suggesting that both pathways can effectively degrade toluene.
3. The major free radical species in the UV-H_2_O_2_ system are ·OH and ^1^O_2_, and ·OH plays a more vital role in the degradation.
4. Experiments employing UV/H_2_O_2_ to generate ·OH for the degradation of toluene confirm that the reaction rate equation fits a first-order kinetic model. The Arrhenius expression derived is k = 1.27 × 10^–2^ exp(− 1241.89/T), with an overall experimental activation energy of 10.33 kJ/mol. This value falls within the range of activation energies calculated theoretically for the initial steps of various reaction pathways (0.56 kJ/mol–13.66 kJ/mol). This correlation indicates the capability of the theoretical kinetic study to accurately predict experimentally determined reaction rates.
5. The characterization of intermediates in the post-reaction solution via GC–MS confirms the presence of benzoic acid (FS8) and various chain alcohols and aldehydes in the UV-H_2_O_2_ system. This substantiates the existence of Path3 in the actual reaction and indicates the ultimate ring-opening and degradation of toluene.

### Supplementary Information

**Additional file 1: Figure S1.** Flow chart of experimental reaction of toluene degradation. **Figure S2.** Results of Geometric Optimization Employing the B3LYP/6-311++G(d,p) Computational Scheme under Solvation Model based on Density (SMD) Conditions. **Figure S3.** Intrinsic Reaction Coordinate (IRC) Analysis of Each Transition State at the B3LYP/6-311++G(d,p) Level of Theory. **Figure S4.** (a): Comprehensive Reaction Rate Constant Fitting at a Reaction Temperature of 25 °C. (b): Comprehensive Reaction Rate Constant Fitting at a Reaction Temperature of 40 °C. (c): Comprehensive Reaction Rate Constant Fitting at a Reaction Temperature of 50 °C. (d): Comprehensive Reaction Rate Constant Fitting at a Reaction Temperature of 60 °C. (e): Comprehensive Reaction Rate Constant Fitting at a Reaction Temperature of 70 °C. **Figure S5.** Fitting of the Experimental Comprehensive Reaction Arrhenius Equation. **Figure S6.** (a) Arrhenius Equation Fitting for the Reaction IS+·OH → IM1 + H_2_O. (c) Arrhenius Equation Fitting for the Reaction IS+·OH → IM6. (e) Arrhenius Equation Fitting for the Reaction IS+·OH → IM8 + H_2_O. (f) Arrhenius Equation Fitting for the Reaction FS8+·OH → IM14. (g) Arrhenius Equation Fitting for the Reaction FS8+·OH → IM15. **Table S1.** T1 Diagnostic Values for All Species in the Reaction under CCSD/cc-pVDZ. **Table S2.** (a) Experimental instrument for degradation of toluene and model thereof. (b) Experimental Reagent specifications and supplier for degradation of toluene. **Table S3.** (a): Absolute Energy Data for All Species Calculated Using the B3LYP/6-311++G(d,p) Computational Scheme. (b): Absolute Energy Data for All Species Calculated at the G4MP2 Level of Theory. **Table S4.** Cartesian Coordinates of Each Species Calculated Using the B3LYP/6-311++G(d,p) Computational Scheme. **Table S5.** Imaginary Frequency Data of Each Transition State Calculated at the B3LYP/6-311++G(d,p) Level of Theory. **Table S6.** Free Energy Data of Various Reaction Species at 303 K–340 K (in atomic units, a.u.).

## Data Availability

The data used to support the findings of this study are available from the corresponding author upon request.
