# Supplementary material for: A theoretical study on toluene oxidization by OH radical
Source: BMC Chem. 2024 Apr 12;18(1):72. doi: 10.1186/s13065-024-01163-w (PMC11015615; doi:10.1186/s13065-024-01163-w)
Supplement: Supplementary file 1 — Additional file 1: Figure S1. Flow chart of experimental reaction of toluene degradation. Figure S2. Results of Geometric Optimization Employing the B3LYP/6-311++G(d,p) Computational Scheme under Solvation Model based on Density (SMD) Conditions. Figure S3. Intrinsic Reaction Coordinate (IRC) Analysis of Each Transition State at the B3LYP/6-311++G(d,p) Level of Theory. Figure S4. (a): Comprehensive Reaction Rate Constant Fitting at a Reaction Temperature of 25 °C. (b): Comprehensive Reaction Rate Constant Fitting at a Reaction Temperature of 40 °C. (c): Comprehensive Reaction Rate Constant Fitting at a Reaction Temperature of 50 °C. (d): Comprehensive Reaction Rate Constant Fitting at a Reaction Temperature of 60 °C. (e): Comprehensive Reaction Rate Constant Fitting at a Reaction Temperature of 70 °C. Figure S5. Fitting of the Experimental Comprehensive Reaction Arrhenius Equation. Figure S6. (a) Arrhenius Equation Fitting for the Reaction IS+·OH → IM1 + H2O. (c) Arrhenius Equation Fitting for the Reaction IS+·OH → IM6. (e) Arrhenius Equation Fitting for the Reaction IS+·OH → IM8 + H2O. (f) Arrhenius Equation Fitting for the Reaction FS8+·OH → IM14. (g) Arrhenius Equation Fitting for the Reaction FS8+·OH → IM15. Table S1. T1 Diagnostic Values for All Species in the Reaction under CCSD/cc-pVDZ. Table S2. (a) Experimental instrument for degradation of toluene and model thereof. (b) Experimental Reagent specifications and supplier for degradation of toluene. Table S3. (a): Absolute Energy Data for All Species Calculated Using the B3LYP/6-311++G(d,p) Computational Scheme. (b): Absolute Energy Data for All Species Calculated at the G4MP2 Level of Theory. Table S4. Cartesian Coordinates of Each Species Calculated Using the B3LYP/6-311++G(d,p) Computational Scheme. Table S5. Imaginary Frequency Data of Each Transition State Calculated at the B3LYP/6-311++G(d,p) Level of Theory. Table S6. Free Energy Data of Various Reaction Species at 303 K–340 K (in atomic units, a.u.). [file 13065_2024_1163_MOESM1_ESM.docx]

**Additional Material for**

**A theoretical study on toluene oxidization by OH radical**

Table

[Figure S1 Flow chart of experimental reaction of toluene degradation 3](#_Toc159101448)

[Figure S2: Results of Geometric Optimization Employing the B3LYP/6-311++G(d,p) Computational Scheme under Solvation Model based on Density (SMD) Conditions. 3](#_Toc159101449)

[Figure S3: Intrinsic Reaction Coordinate (IRC) Analysis of Each Transition State at the B3LYP/6-311++G(d,p) Level of Theory. 11](#_Toc159101450)

[Figure S4(a): Comprehensive Reaction Rate Constant Fitting at a Reaction Temperature of 25°C. 25](#_Toc159101451)

[Figure S4(b): Comprehensive Reaction Rate Constant Fitting at a Reaction Temperature of 40°C. 26](#_Toc159101452)

[Figure S4(c): Comprehensive Reaction Rate Constant Fitting at a Reaction Temperature of 50°C. 27](#_Toc159101453)

[Figure S4(d): Comprehensive Reaction Rate Constant Fitting at a Reaction Temperature of 60°C. 28](#_Toc159101454)

[Figure S4(e): Comprehensive Reaction Rate Constant Fitting at a Reaction Temperature of 70°C. 29](#_Toc159101455)

[Figure S5: Fitting of the Experimental Comprehensive Reaction Arrhenius Equation. 29](#_Toc159101456)

[Figure S6 (a) Arrhenius Equation Fitting for the Reaction IS+·OH→IM1+H_2_O 30](#_Toc159101457)

[Figure S6 (c) Arrhenius Equation Fitting for the Reaction IS+·OH→IM6 31](#_Toc159101458)

[Figure S6 (e) Arrhenius Equation Fitting for the Reaction IS+·OH→IM8+H_2_O 32](#_Toc159101459)

[Figure S6 (f) Arrhenius Equation Fitting for the Reaction FS8+·OH→IM14 33](#_Toc159101460)

[Figure S6 (g) Arrhenius Equation Fitting for the Reaction FS8+·OH→IM15 34](#_Toc159101461)

[Table S1 T1 Diagnostic Values for All Species in the Reaction under CCSD/cc-pVDZ 34](#_Toc159101462)

[Table S2(a) Experimental instrument for degradation of toluene and model thereof 35](#_Toc159101463)

[Table S2(b) Experimental Reagent specifications and supplier for degradation of toluene 35](#_Toc159101464)

[Table S3(a): Absolute Energy Data for All Species Calculated Using the B3LYP/6-311++G(d,p) Computational Scheme. 37](#_Toc159101465)

[Table S3(b): Absolute Energy Data for All Species Calculated at the G4MP2 Level of Theory. 38](#_Toc159101466)

[Table S4: Cartesian Coordinates of Each Species Calculated Using the B3LYP/6-311++G(d,p) Computational Scheme. 40](#_Toc159101467)

[Table S5: Imaginary Frequency Data of Each Transition State Calculated at the B3LYP/6-311++G(d,p) Level of Theory. 66](#_Toc159101468)

[Table S6: Free Energy Data of Various Reaction Species at 303K-340K (in atomic units, a.u.). 67](#_Toc159101469)

# Figure S1 Flow chart of experimental reaction of toluene degradation


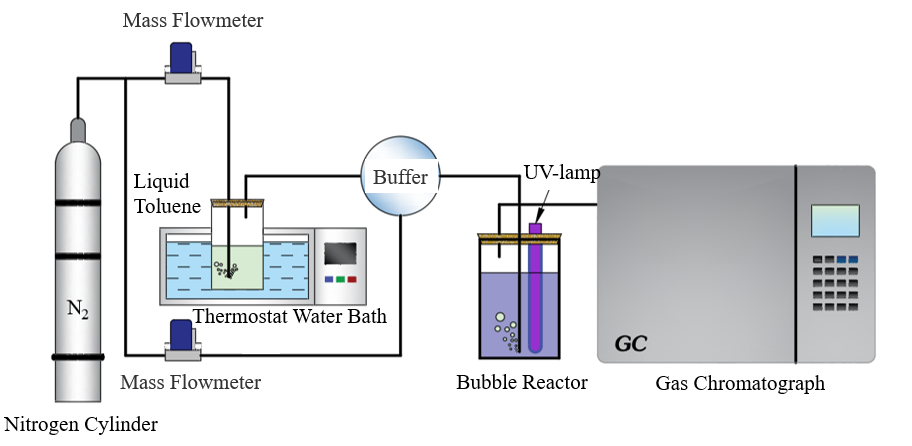


# Figure S2: Results of Geometric Optimization Employing the B3LYP/6-311++G(d,p) Computational Scheme under Solvation Model based on Density (SMD) Conditions.


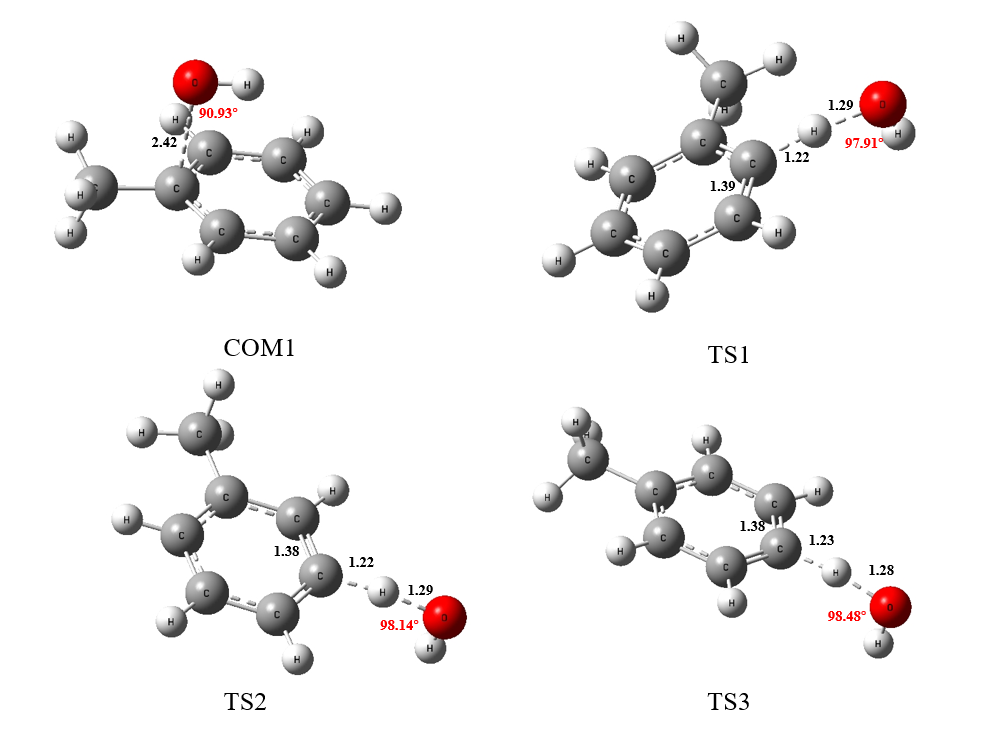

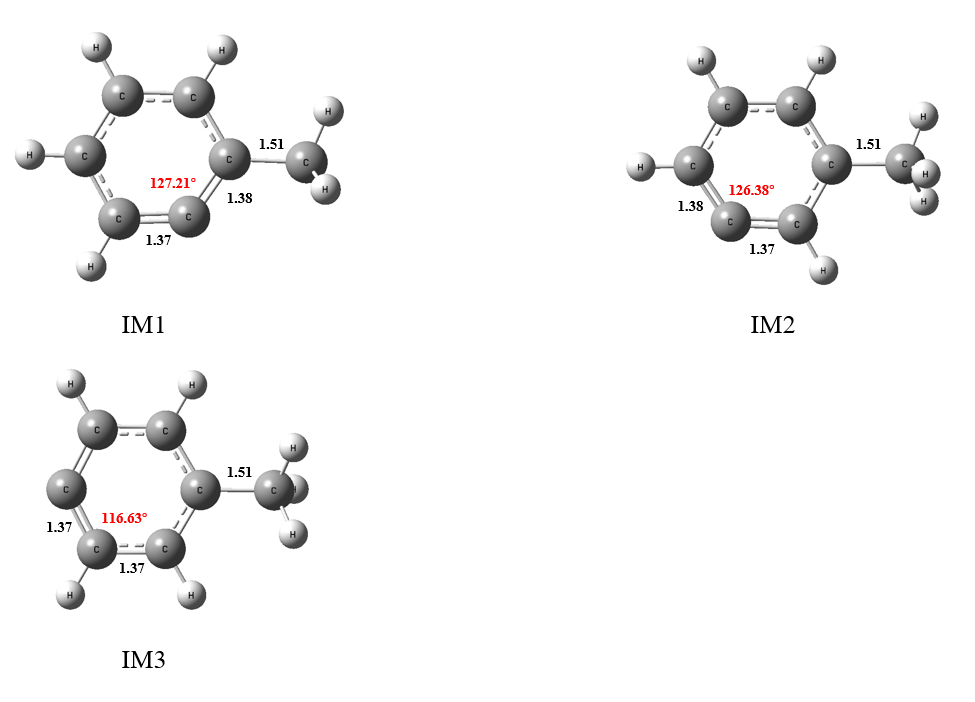

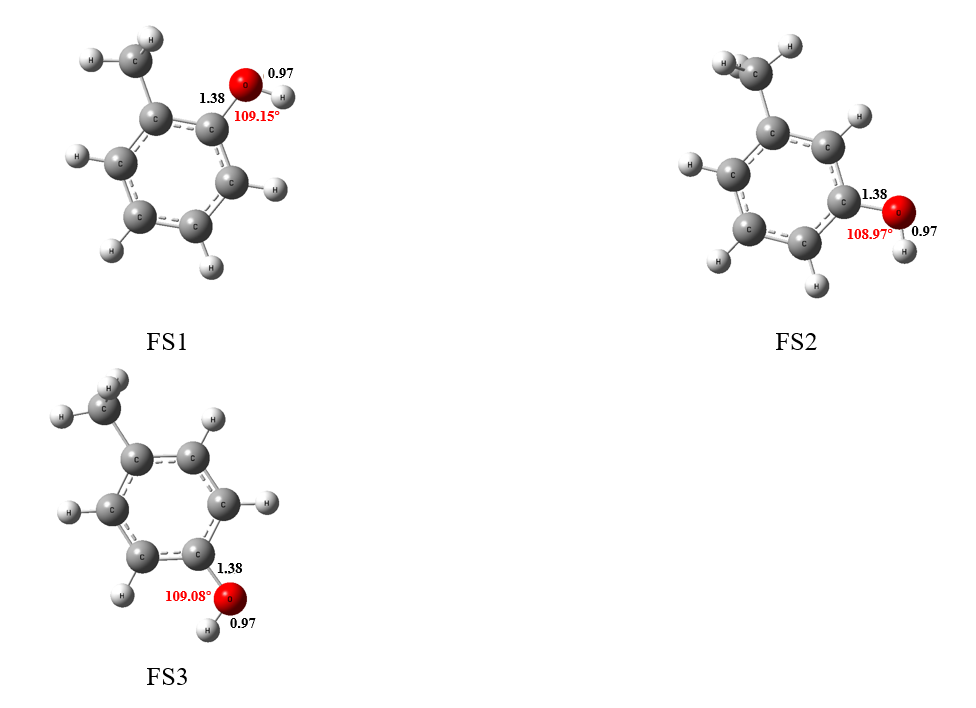

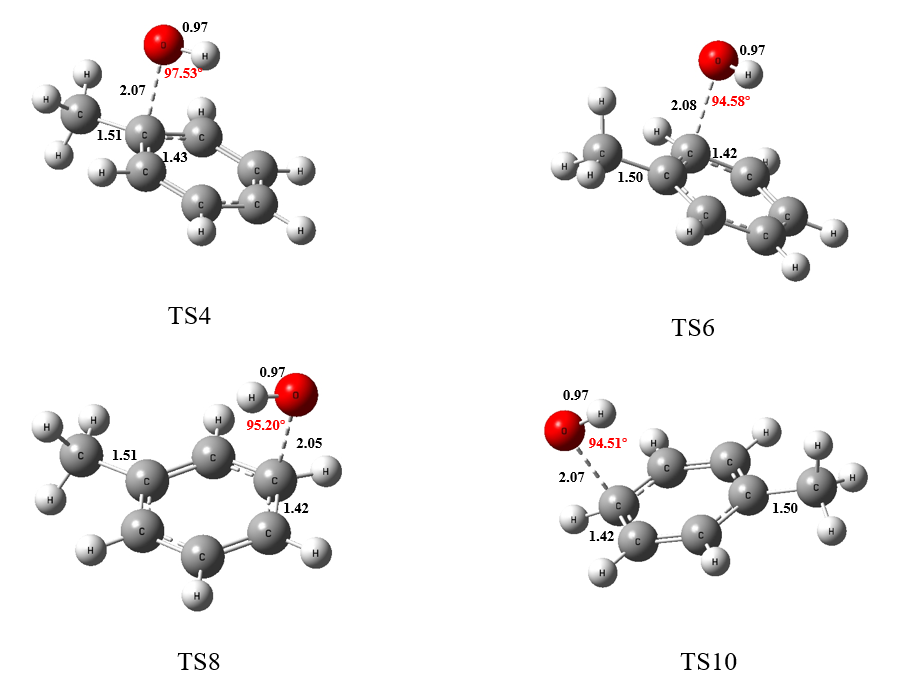

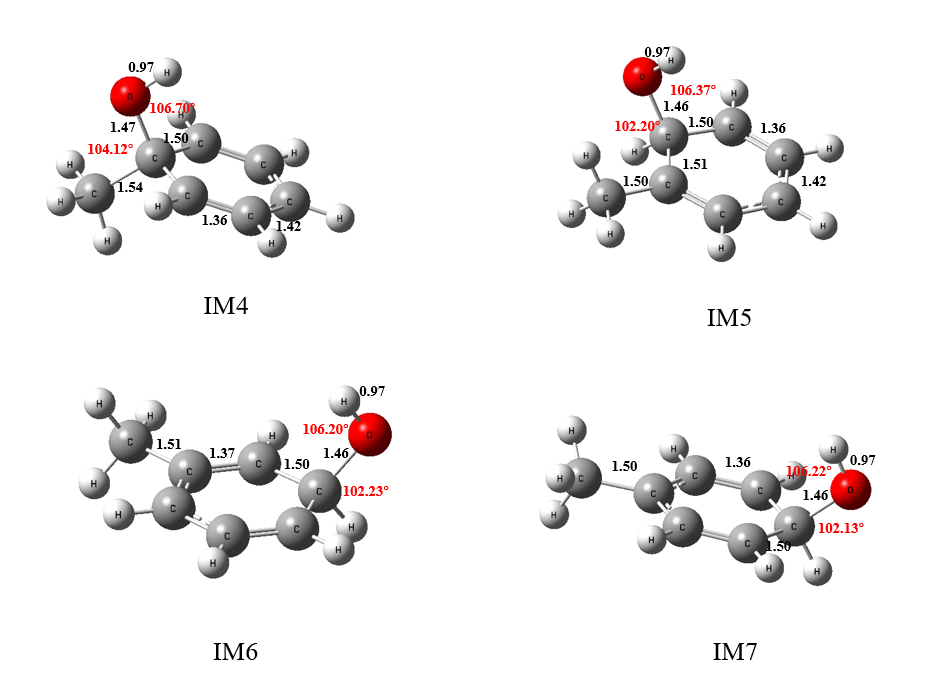

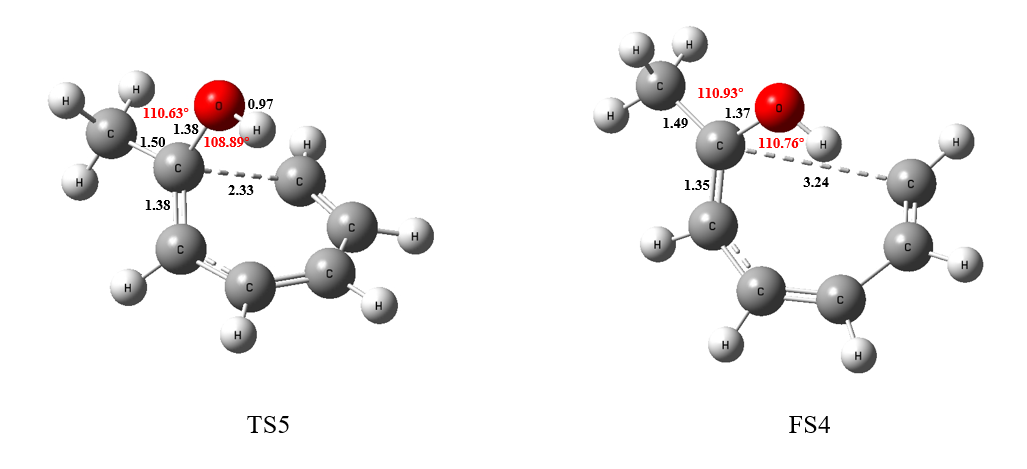

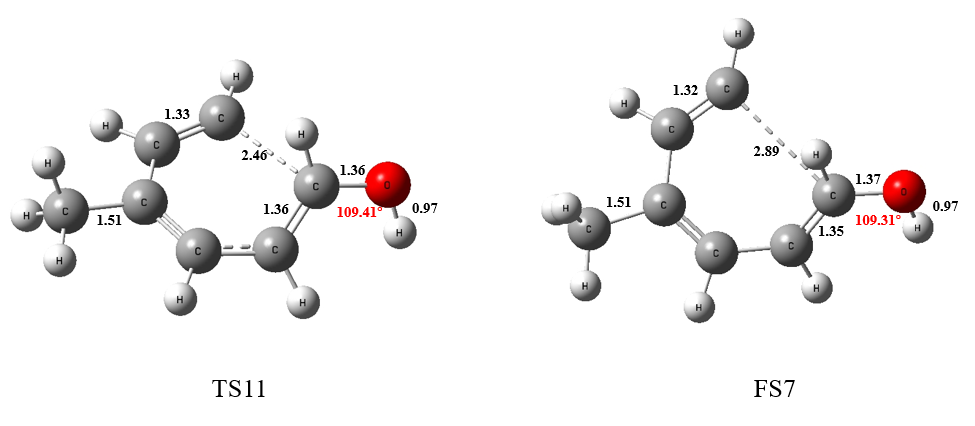

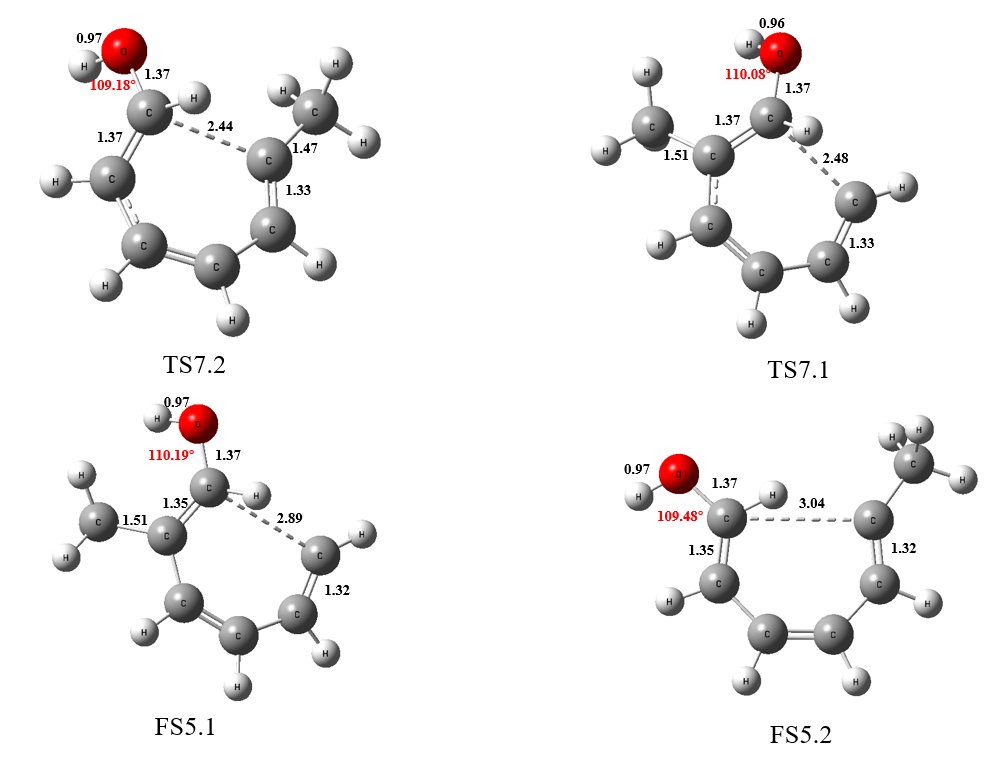

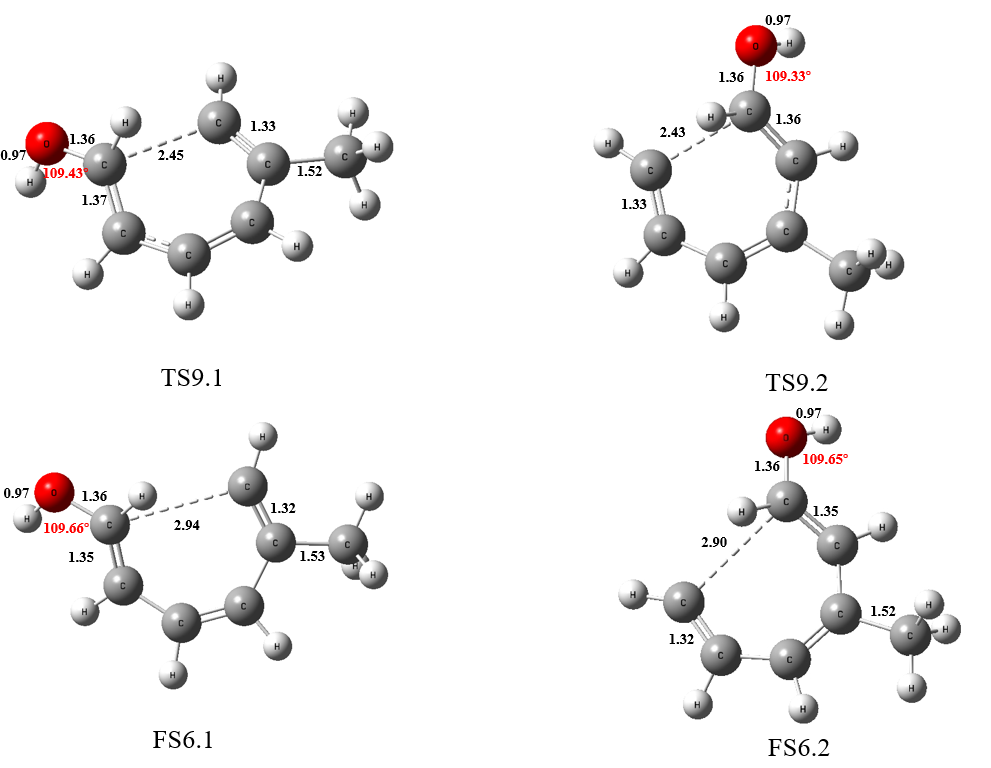

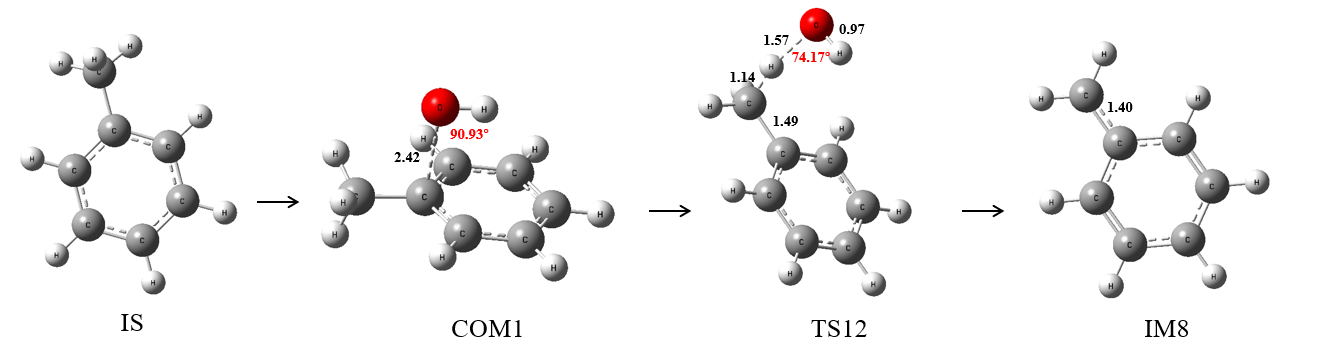

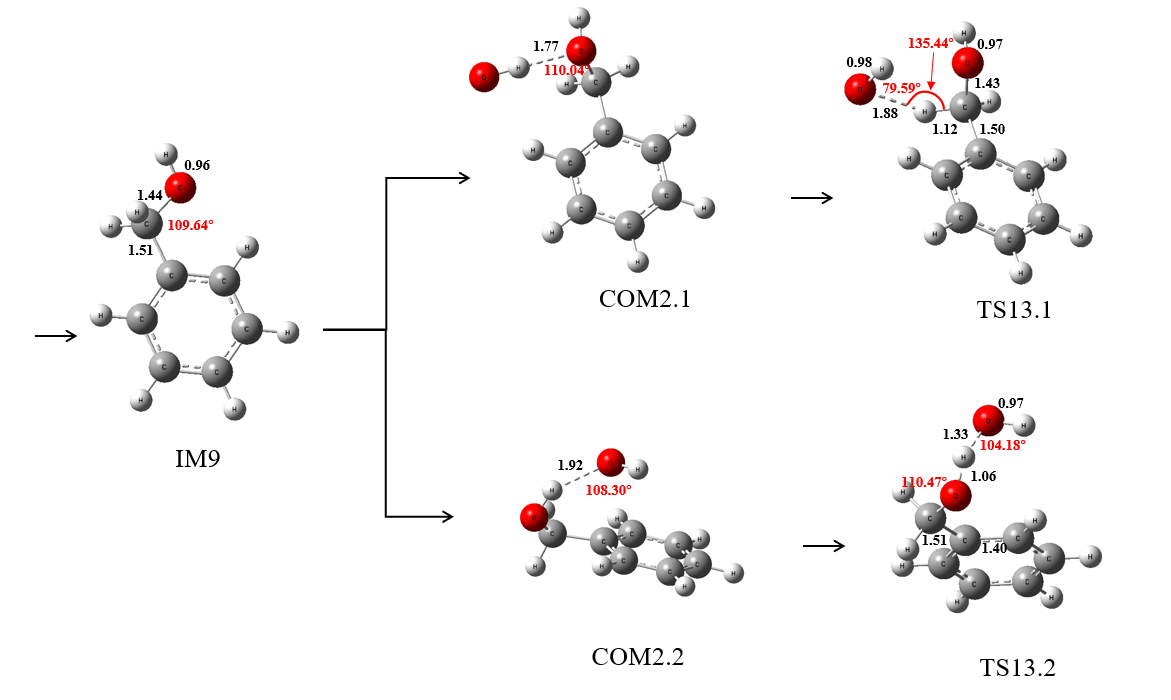

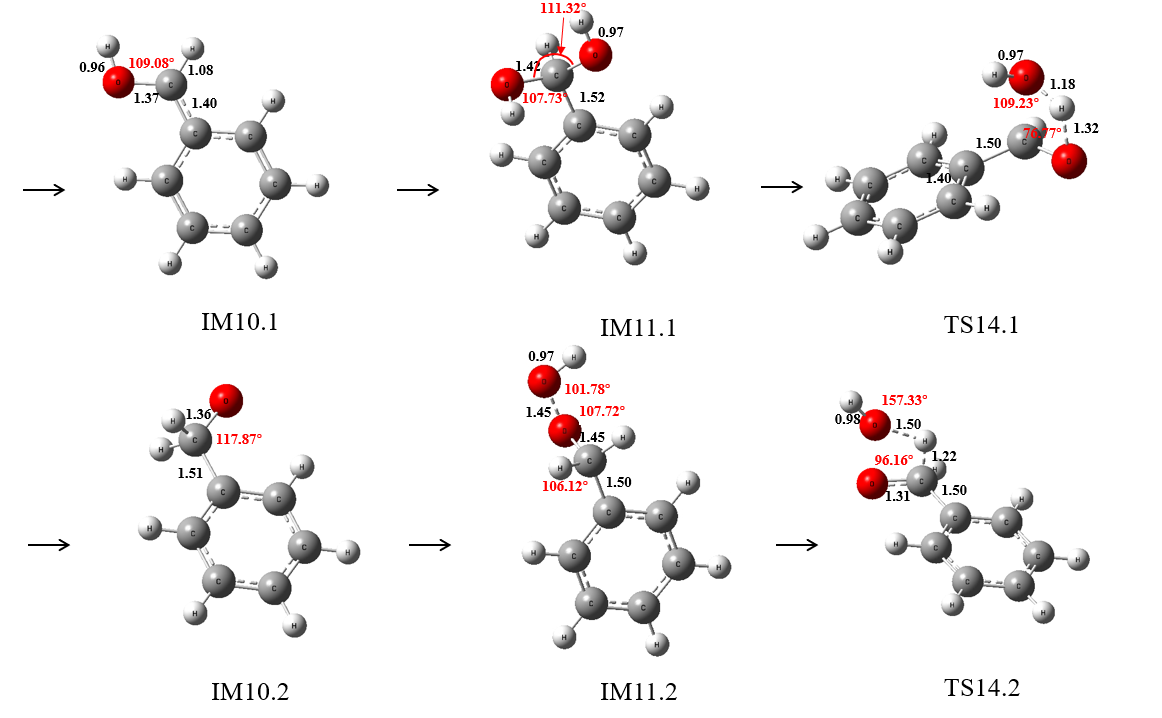

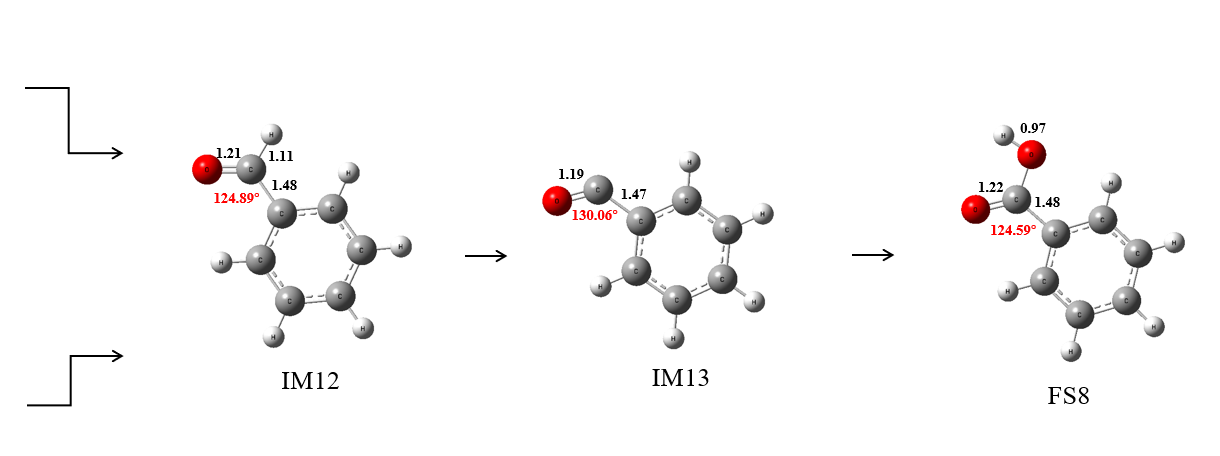

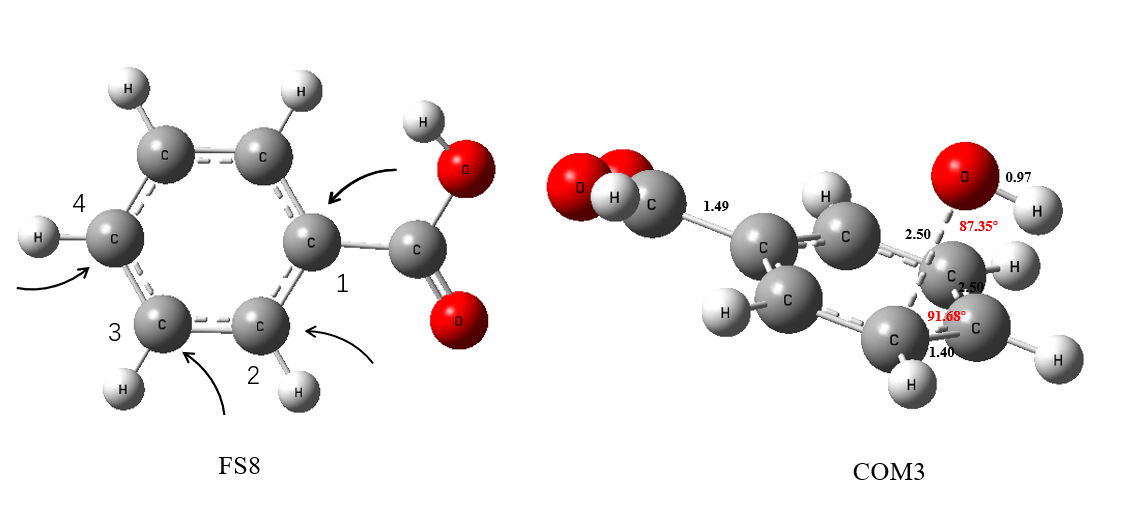

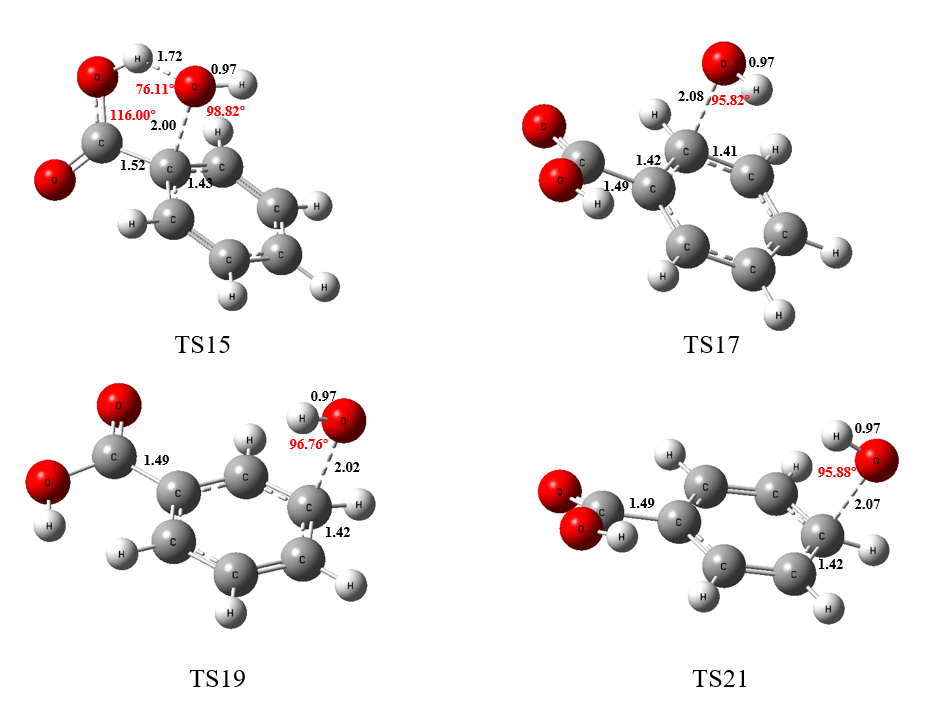

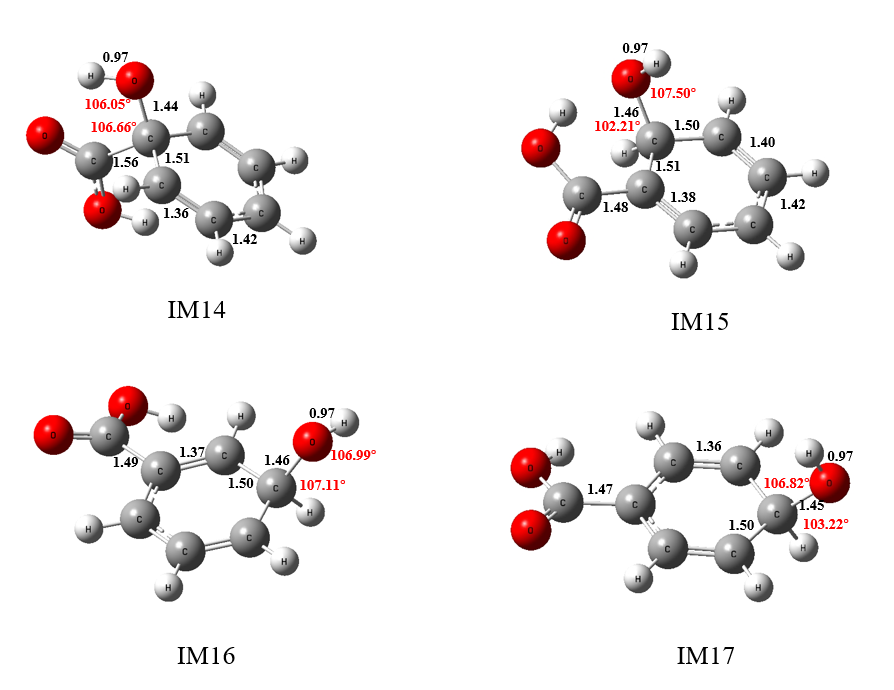

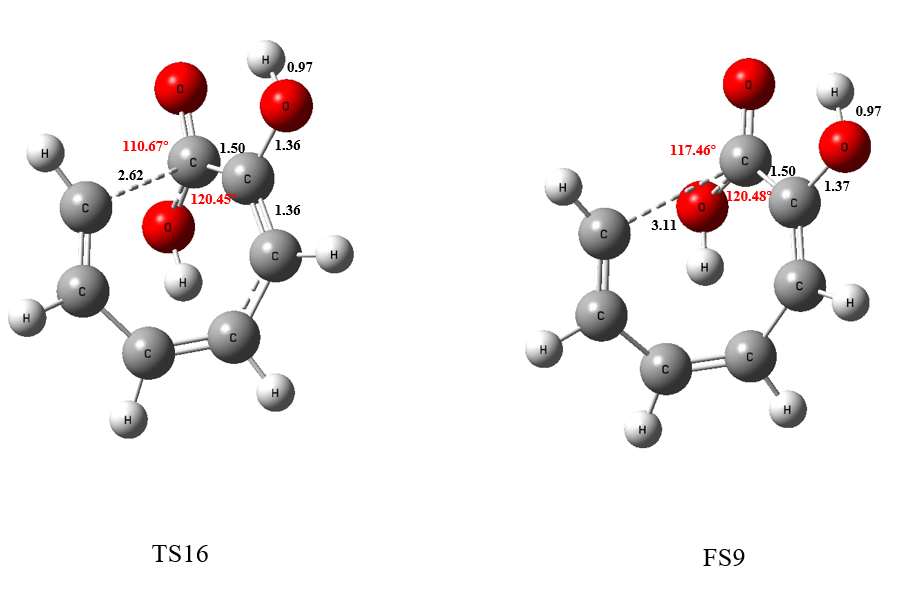

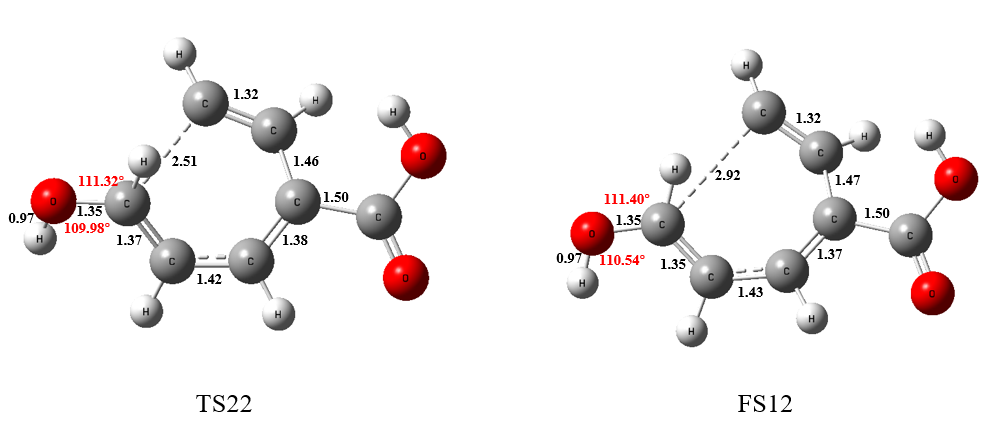

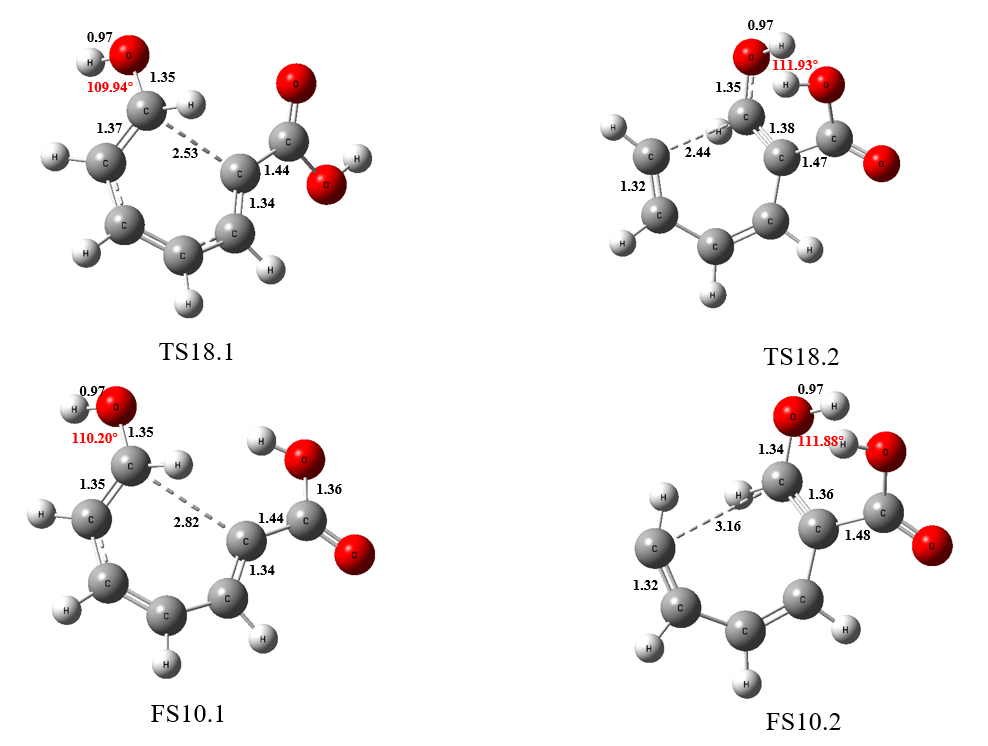

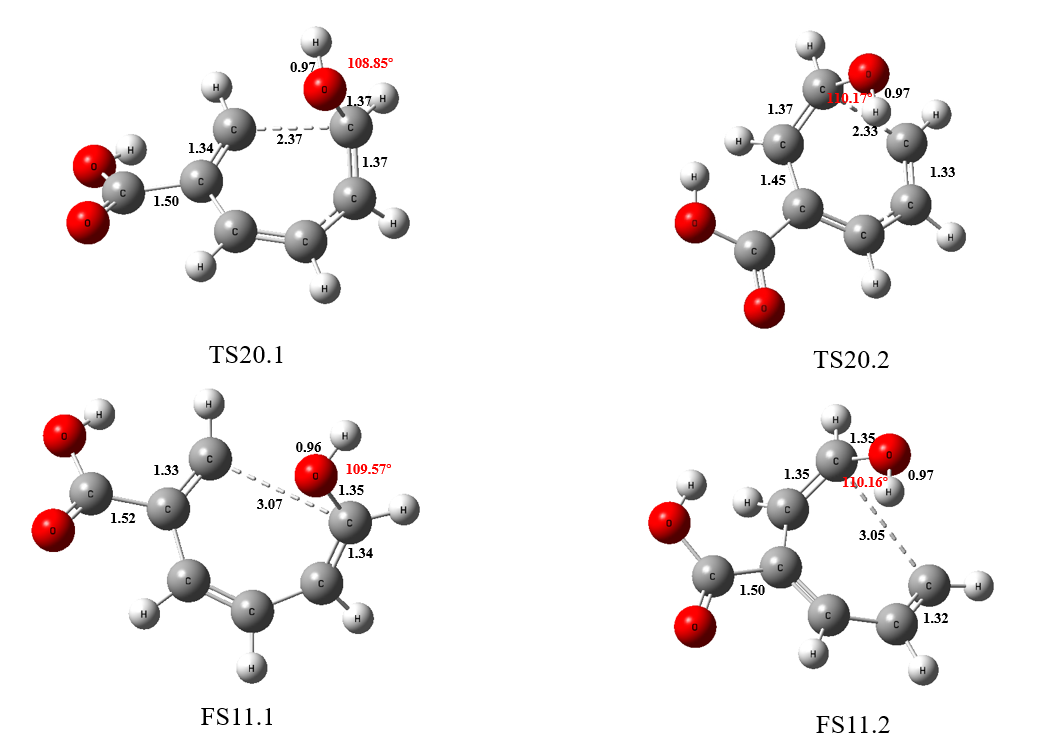


# Figure S3: Intrinsic Reaction Coordinate (IRC) Analysis of Each Transition State at the B3LYP/6-311++G(d,p) Level of Theory.


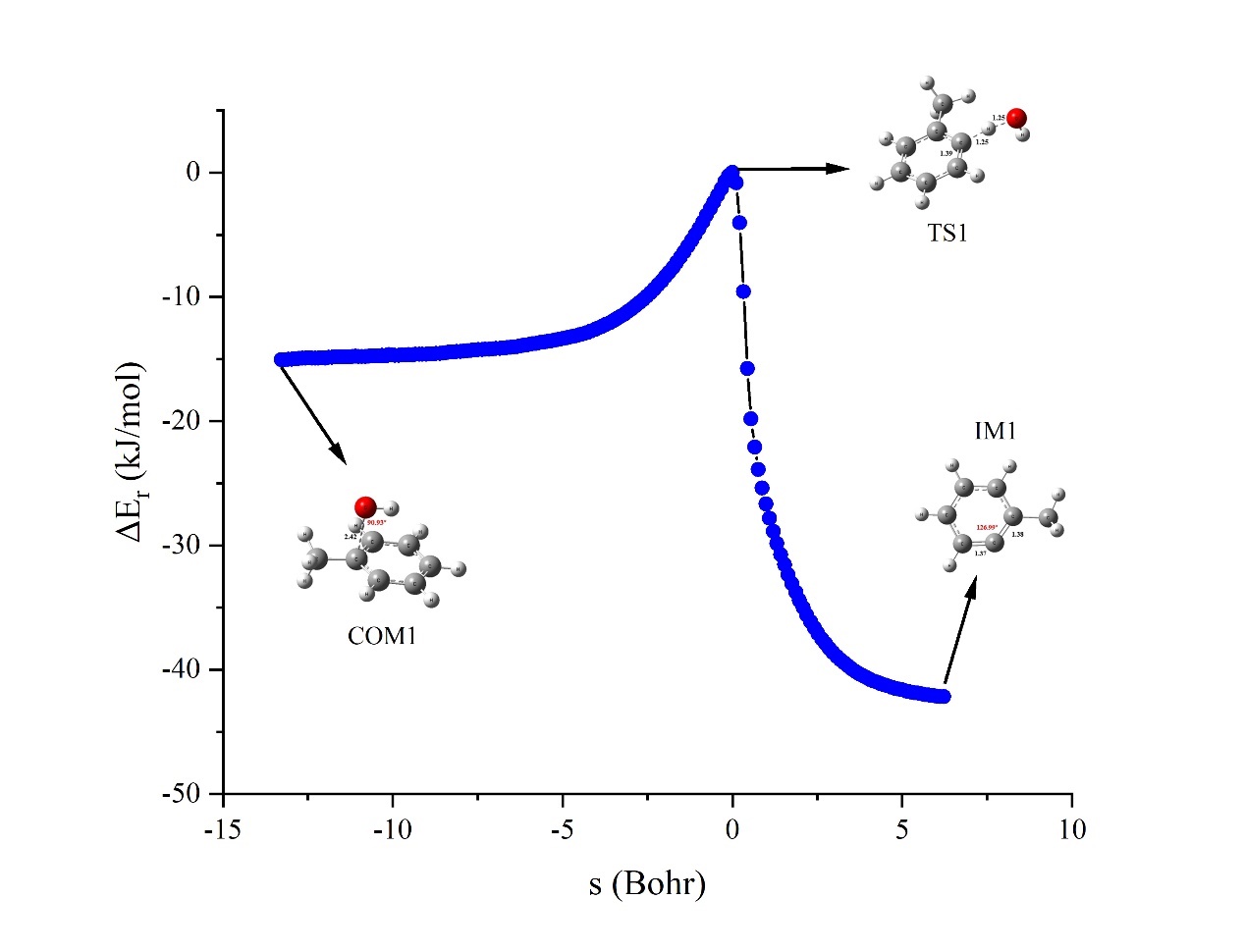


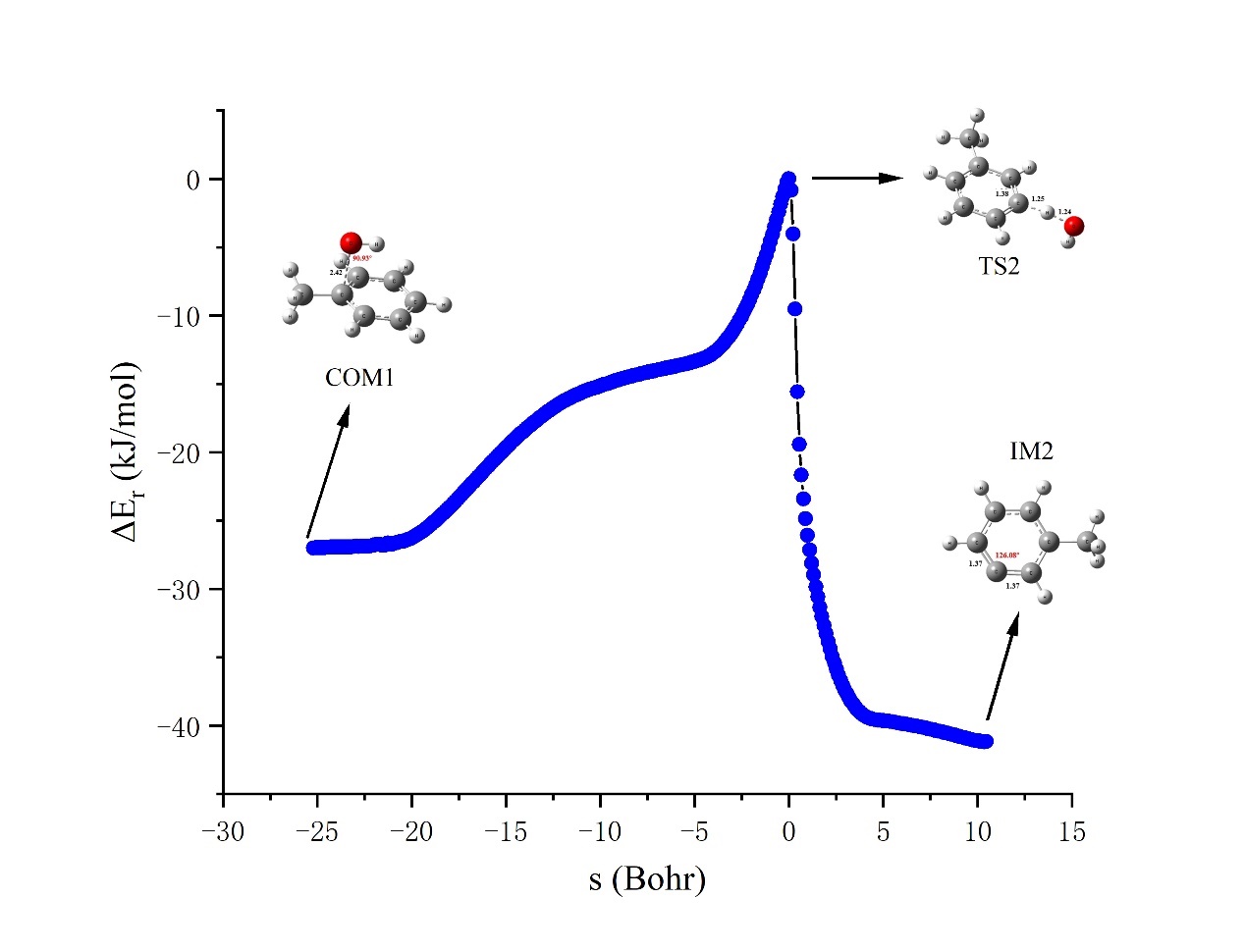

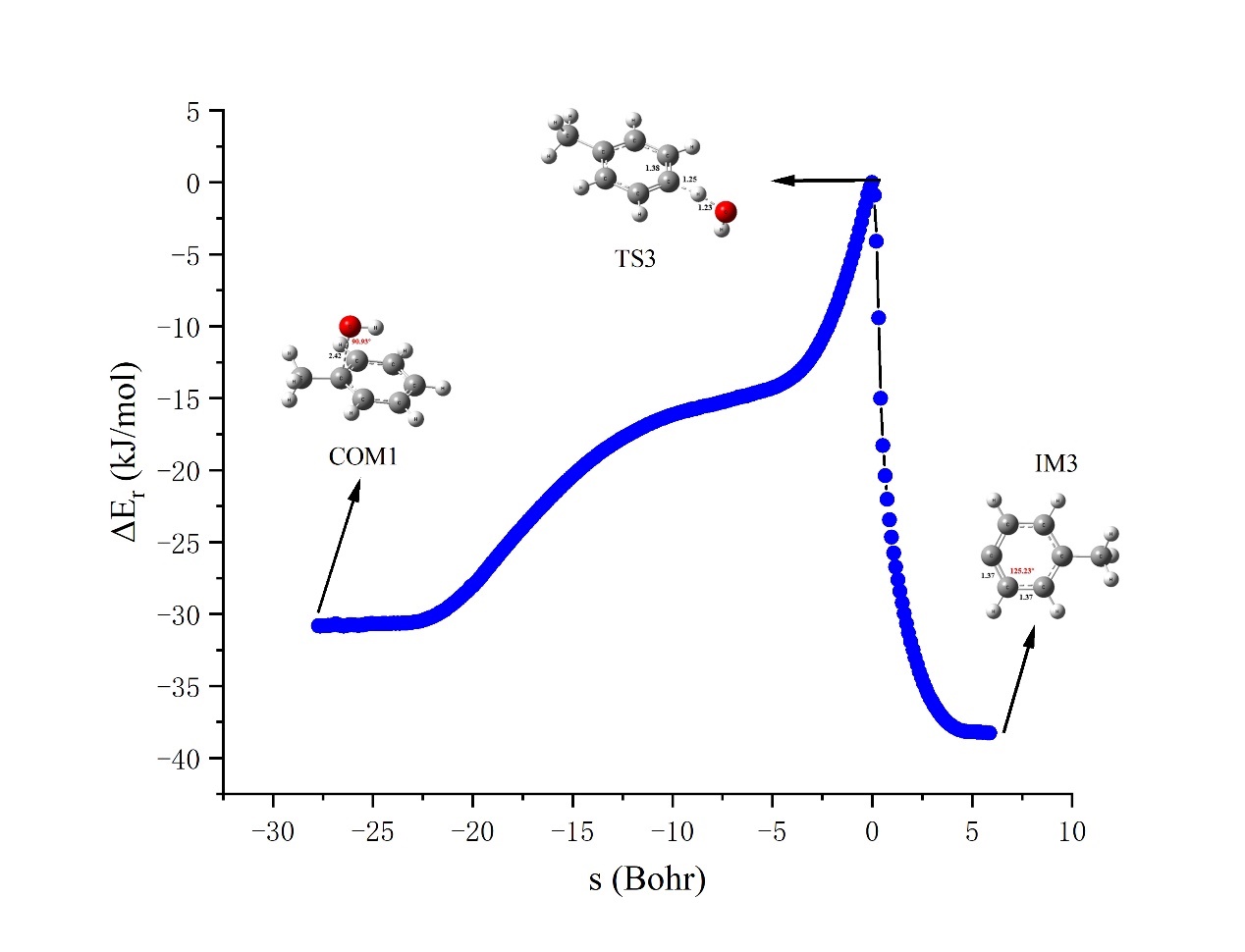

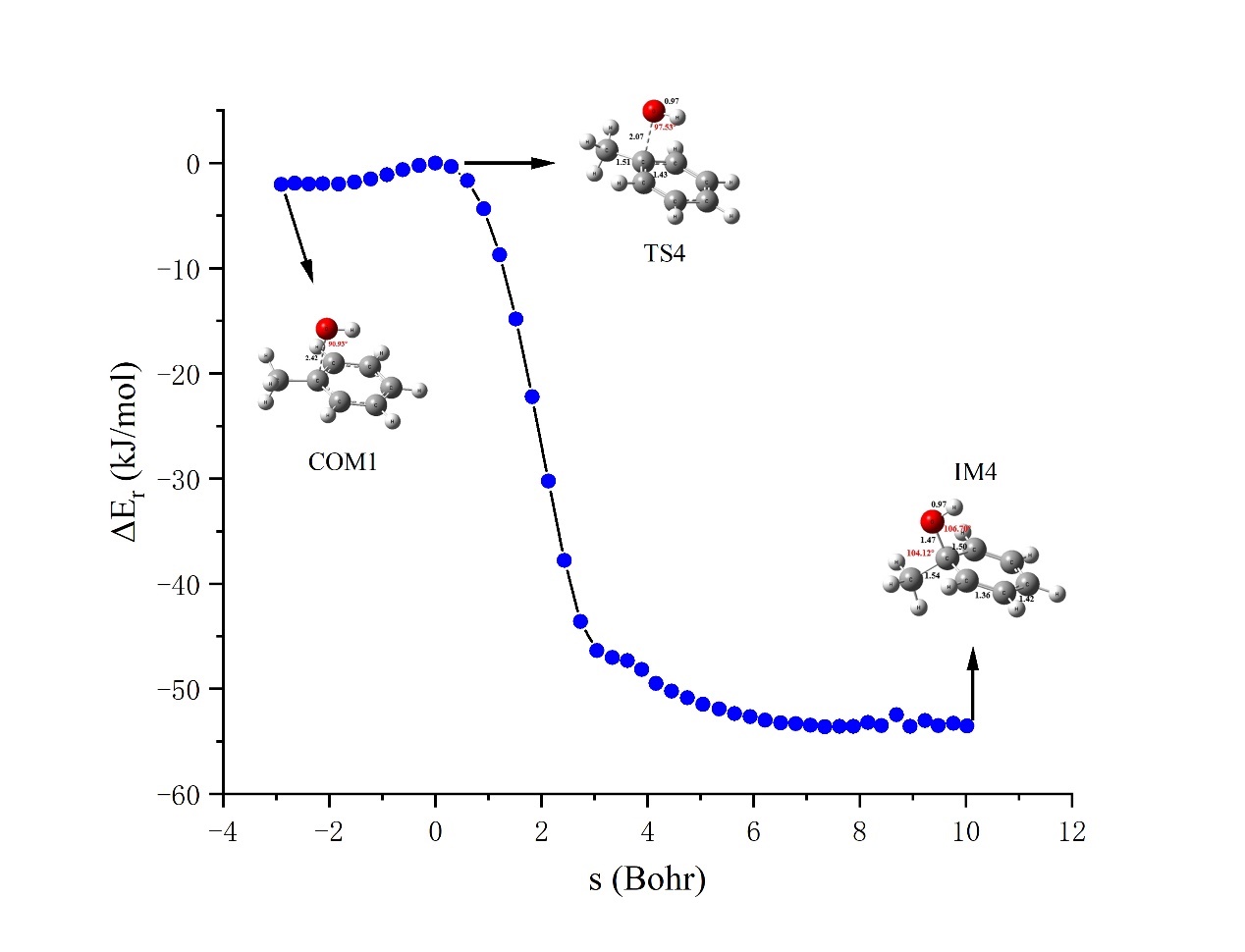

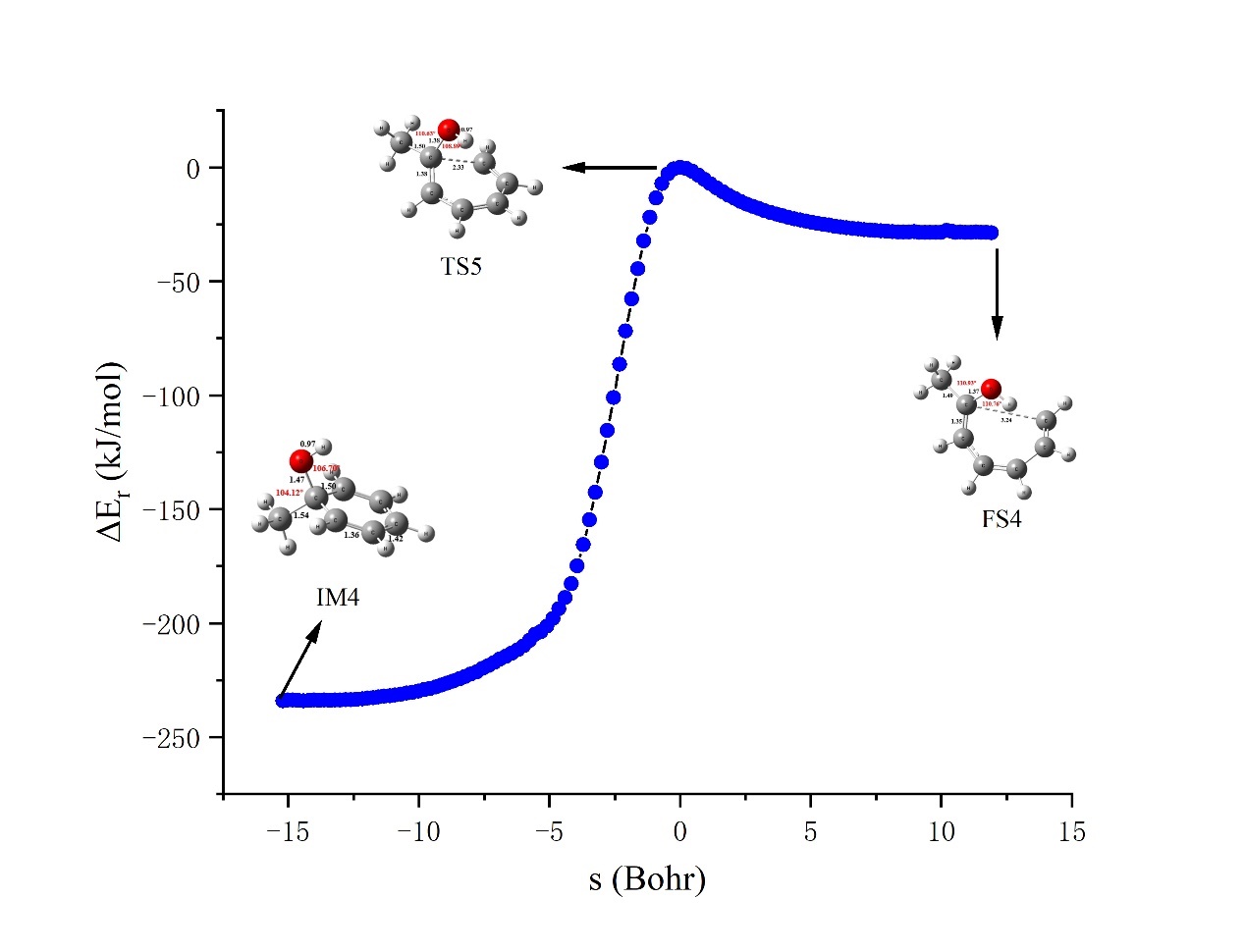

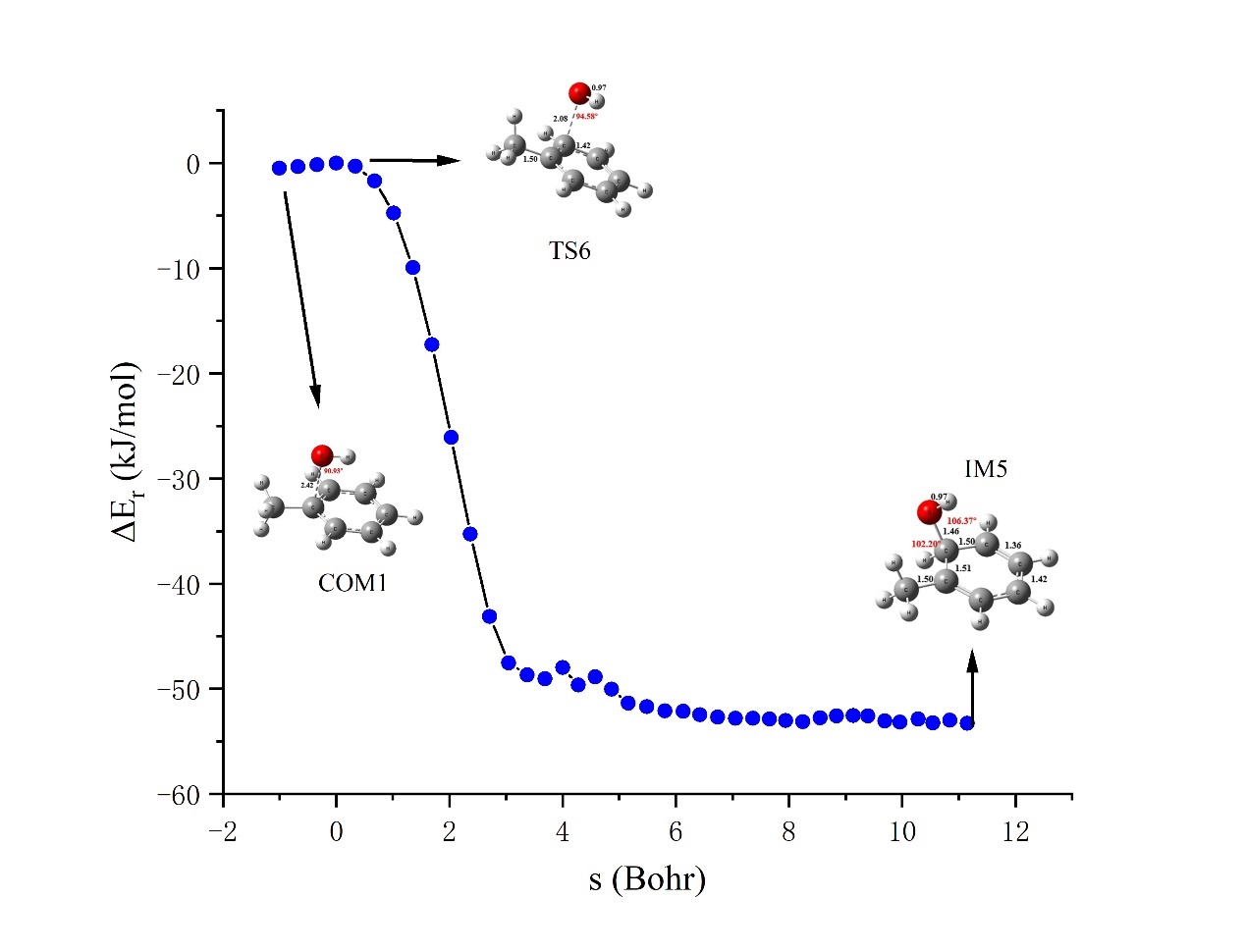

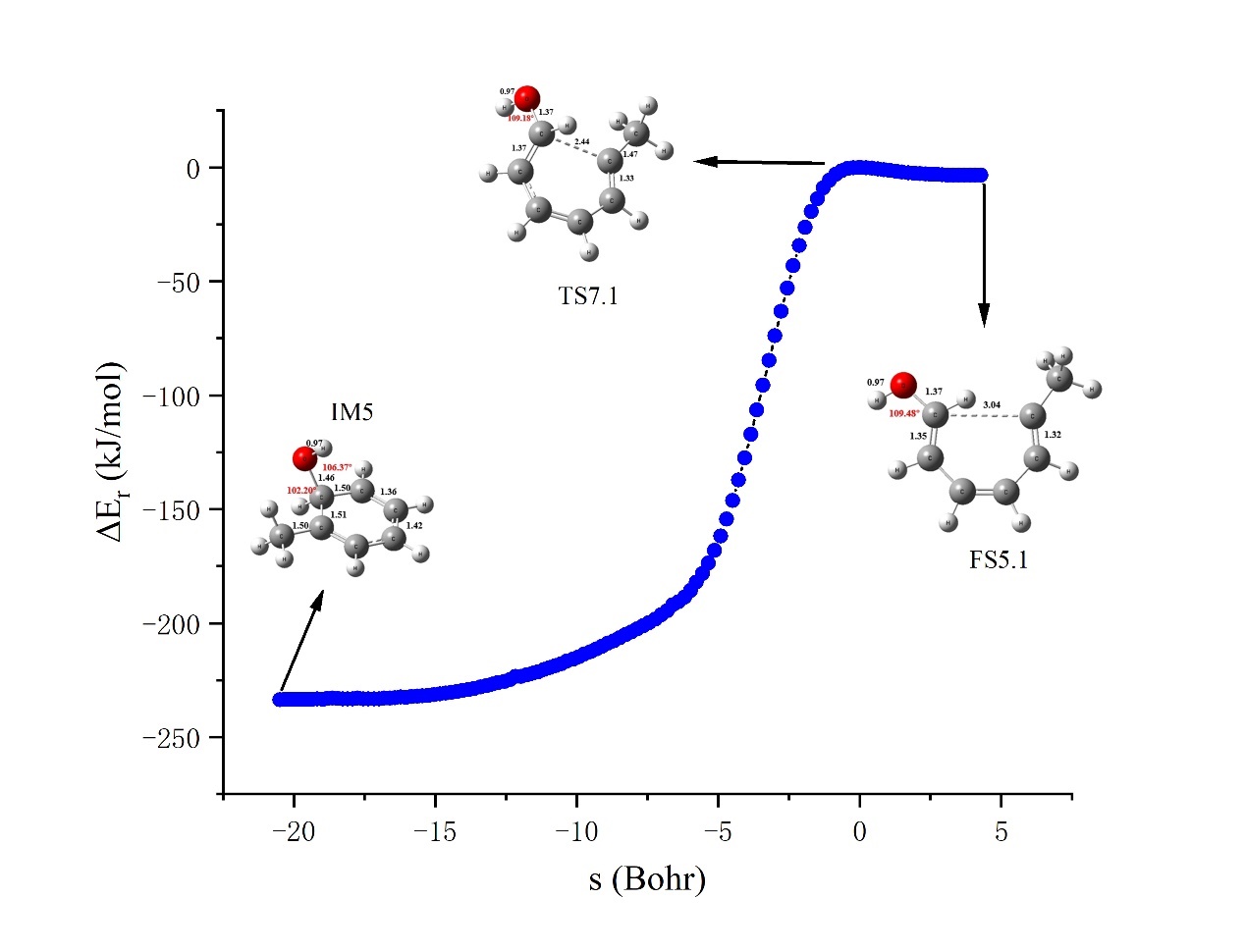

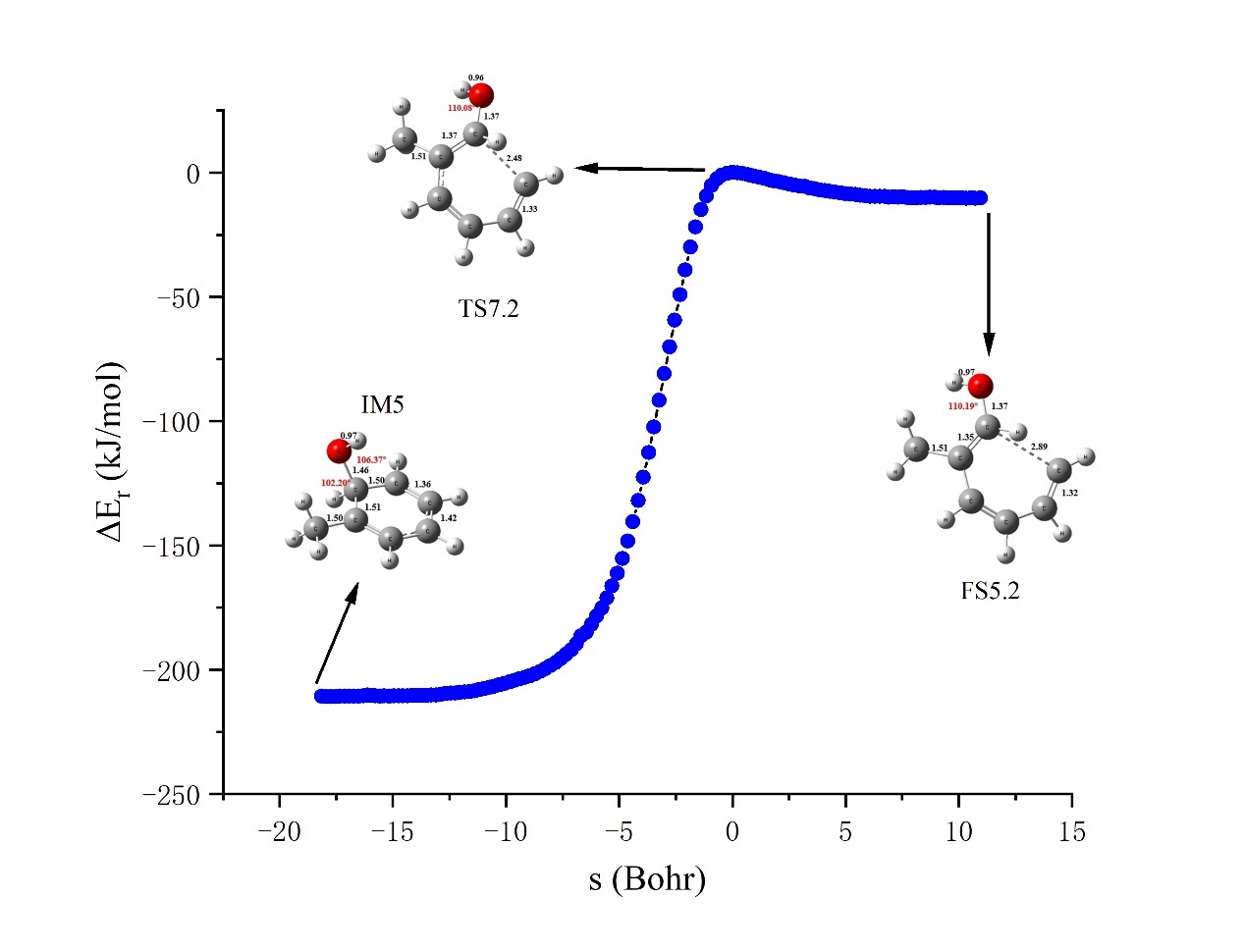

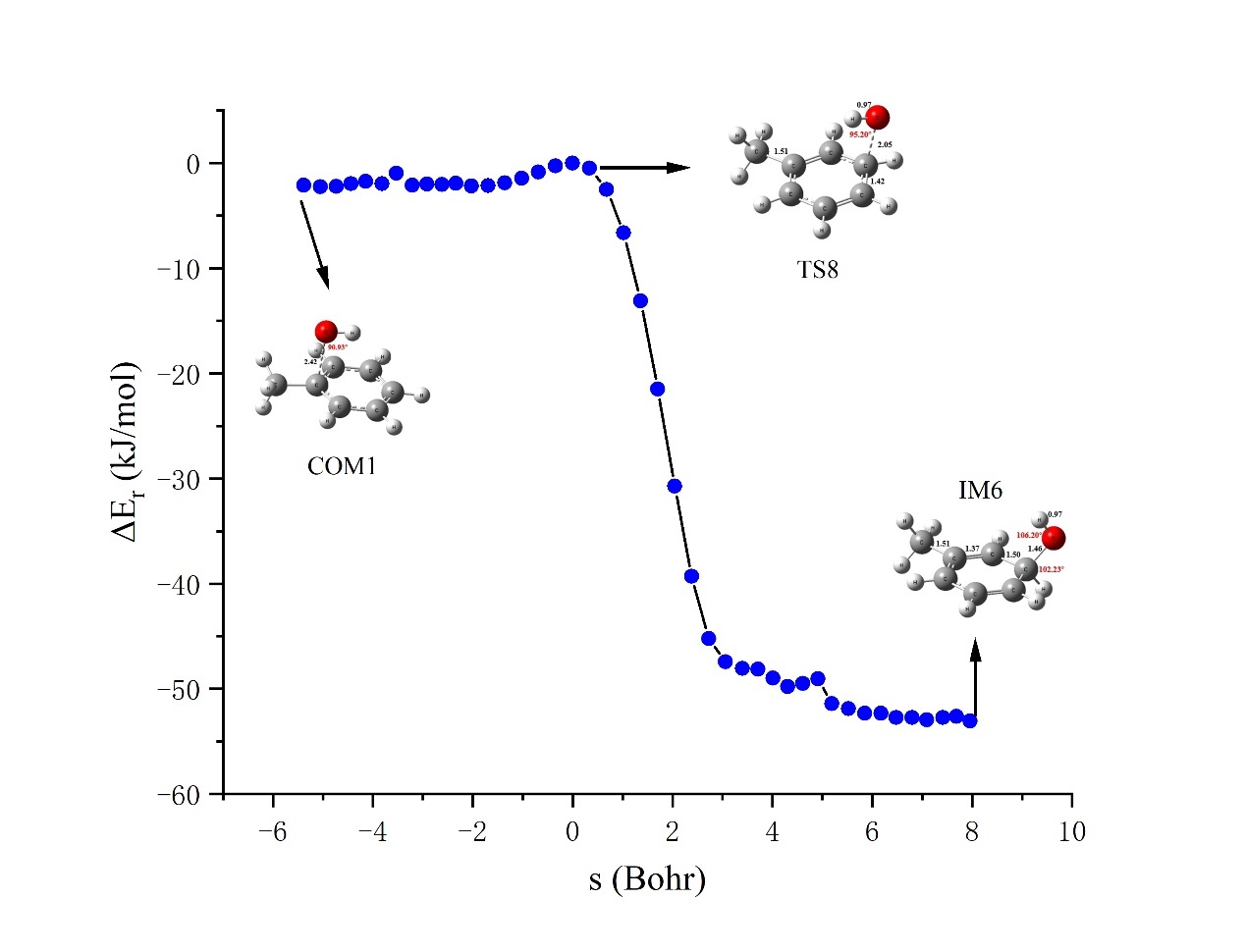

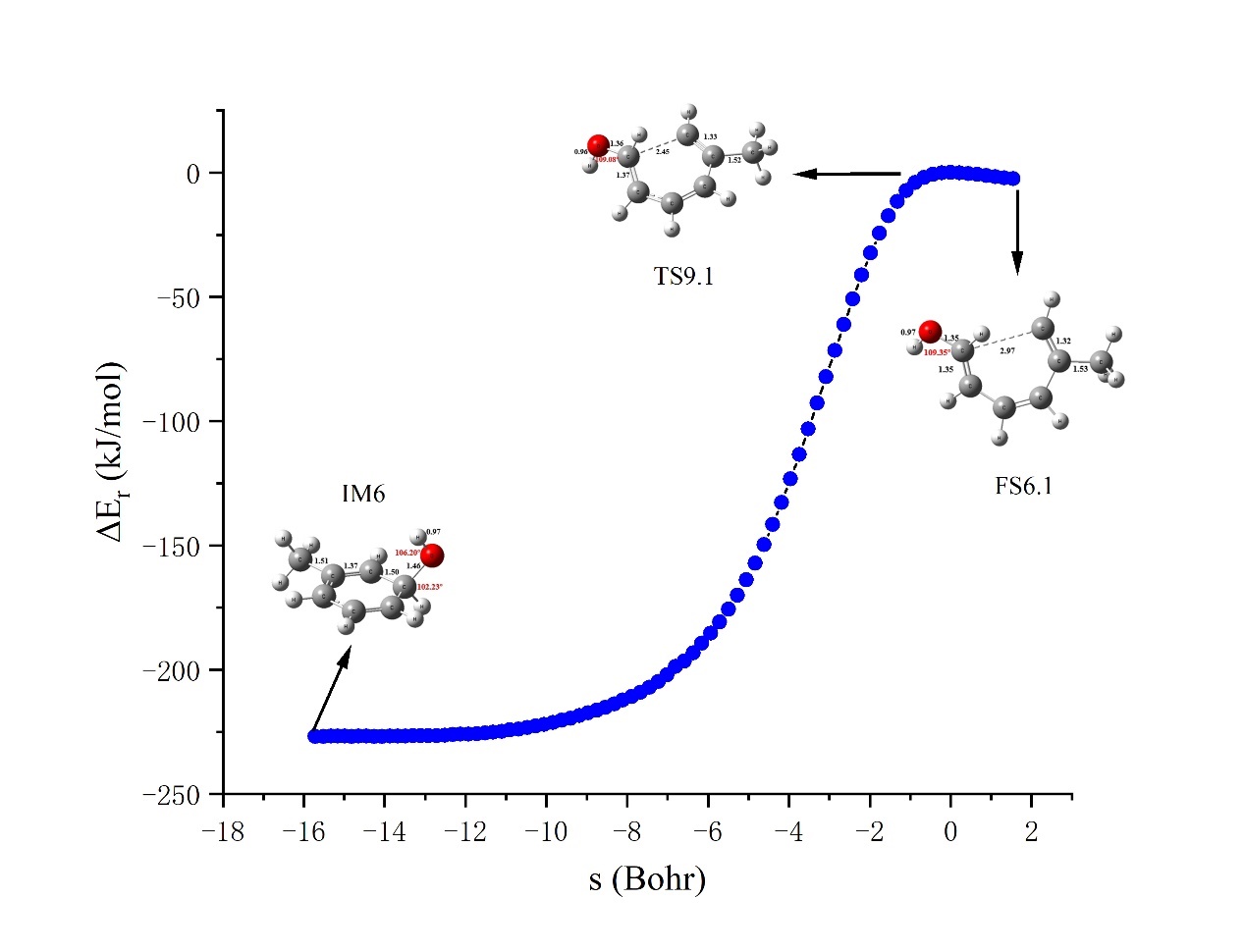

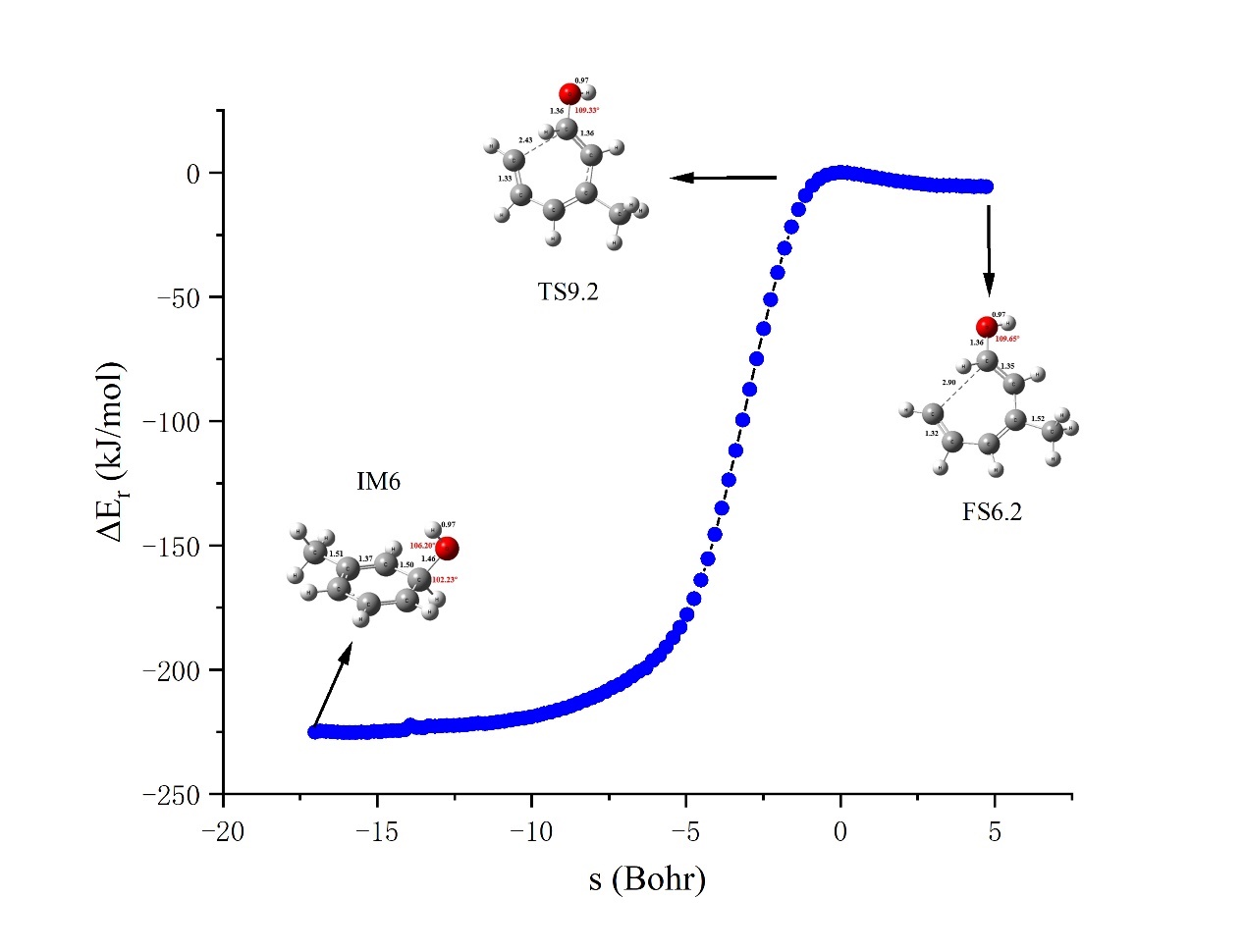

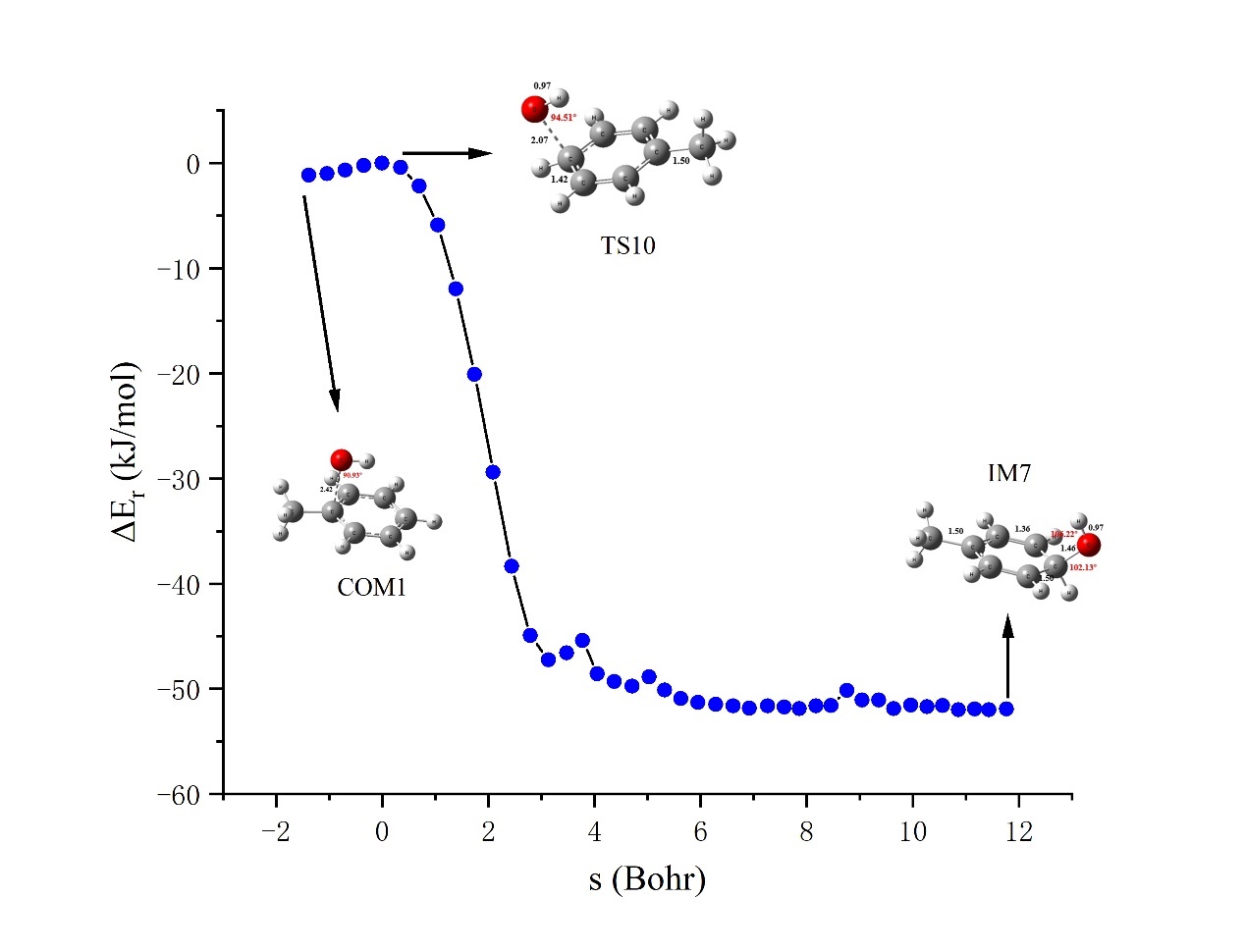

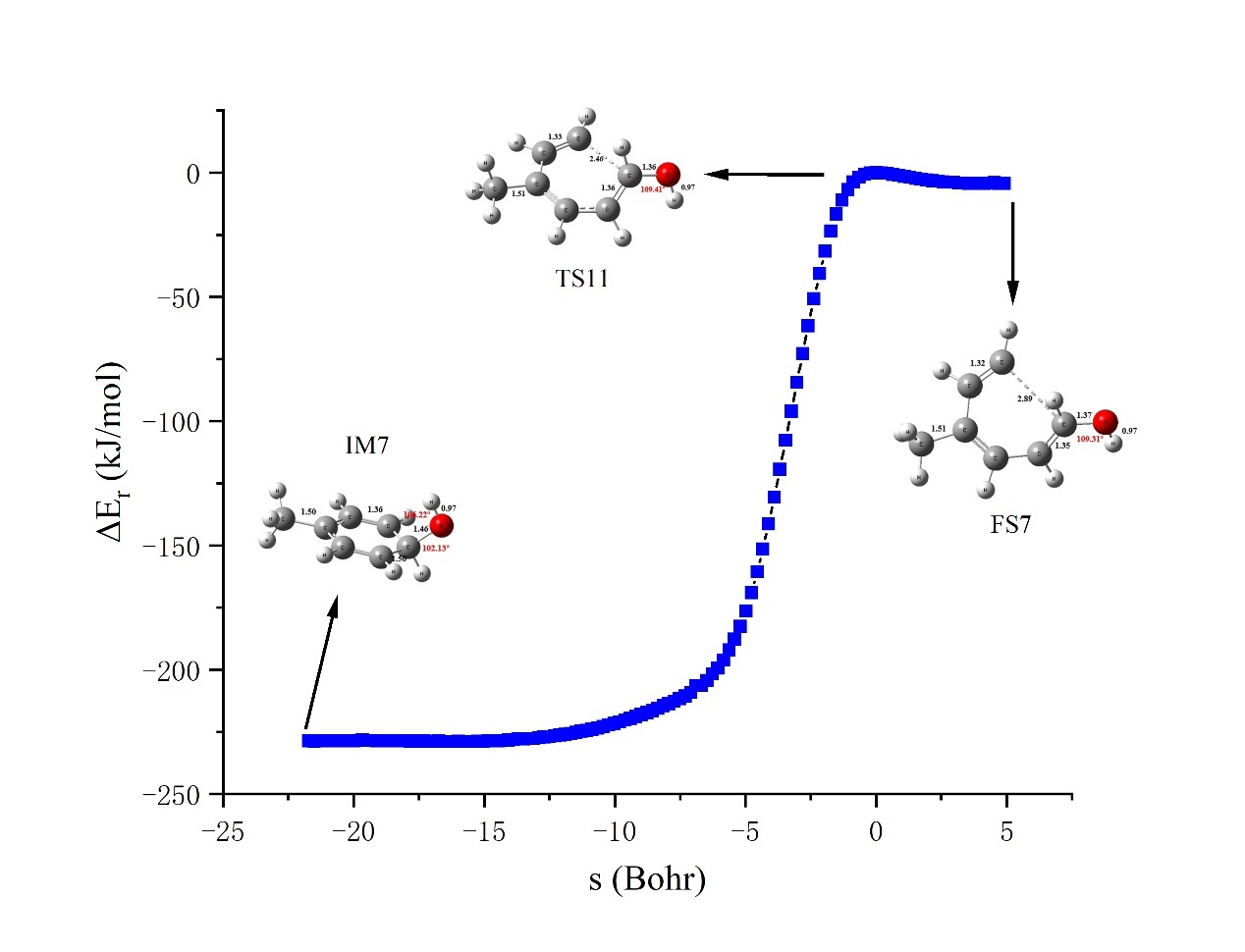

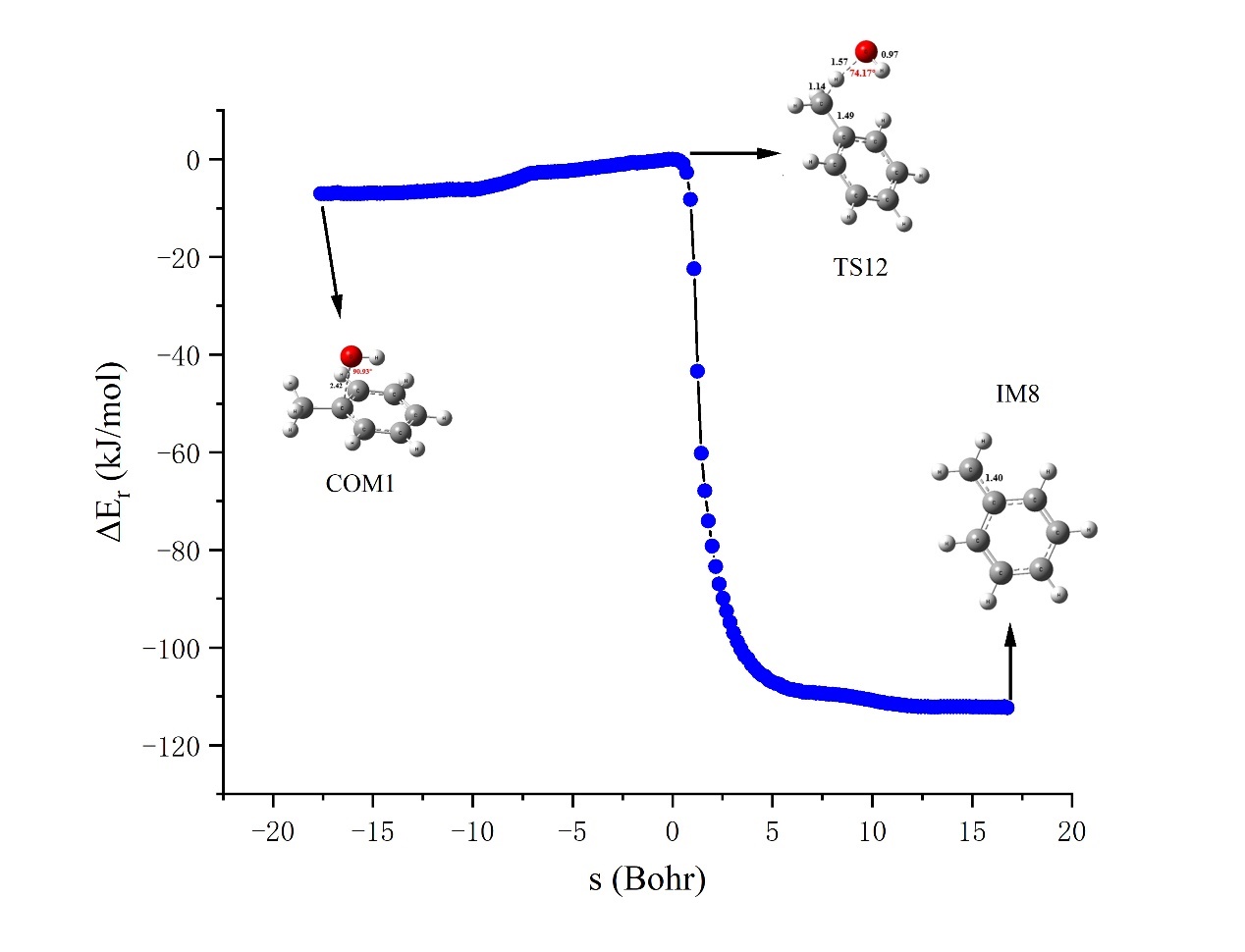

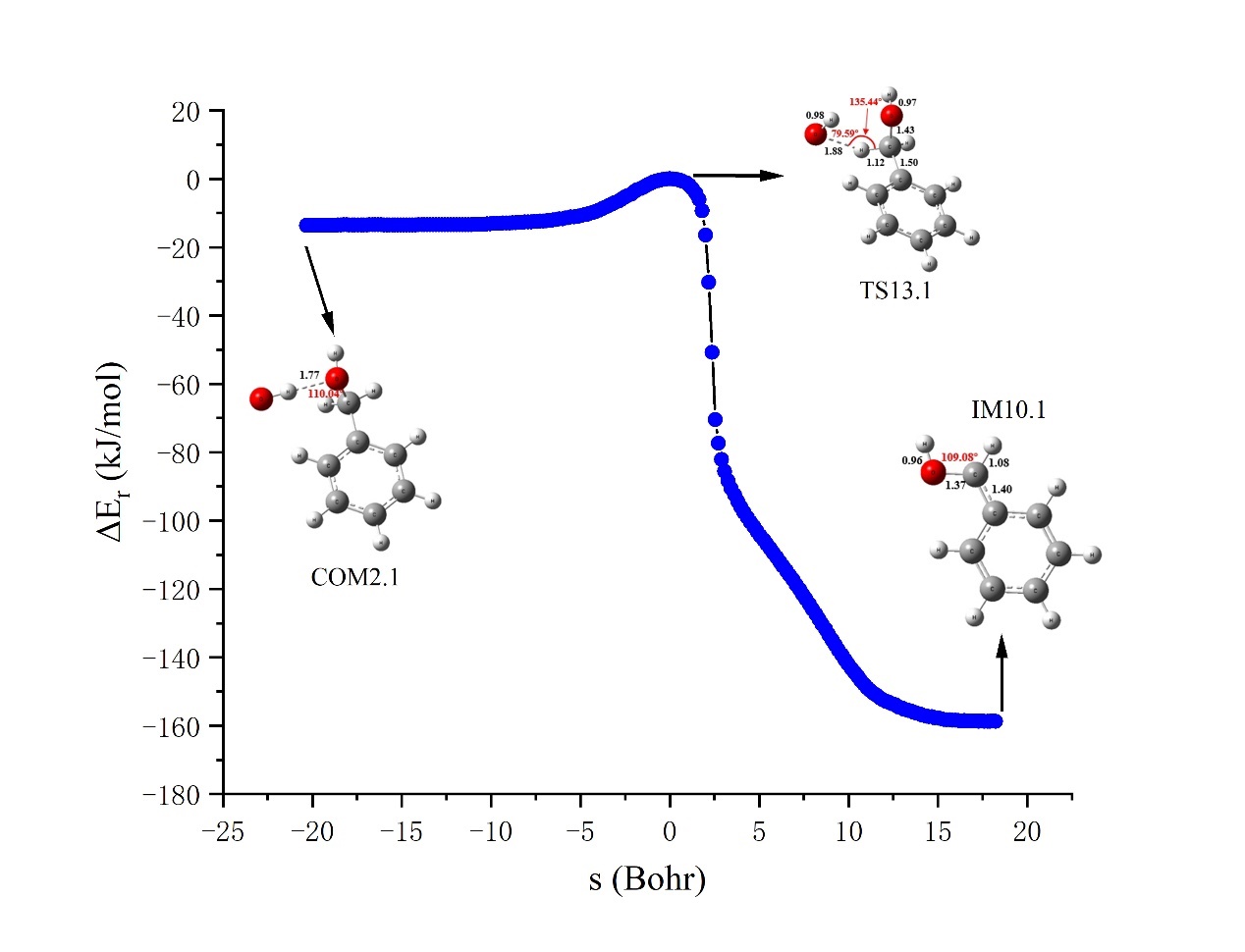

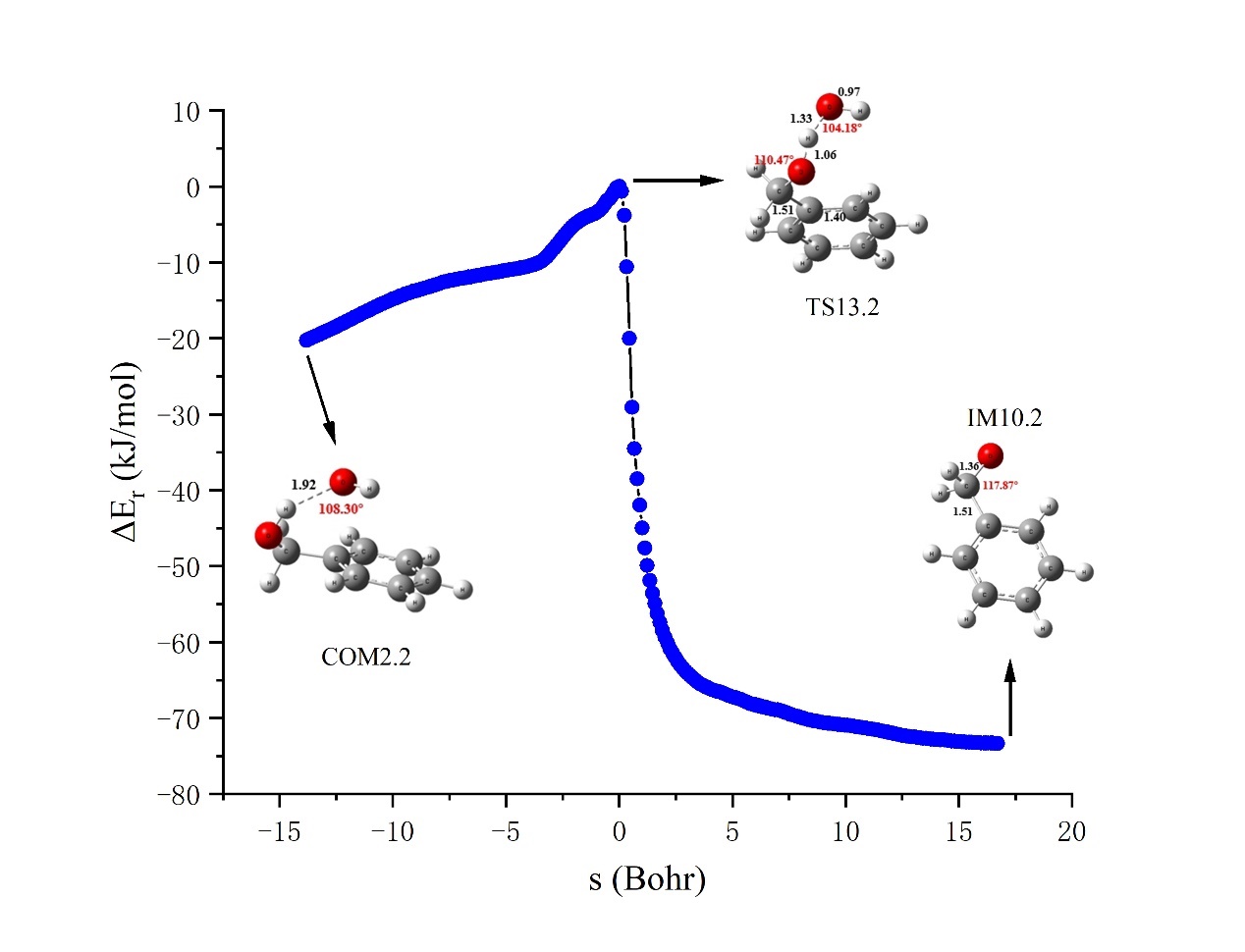

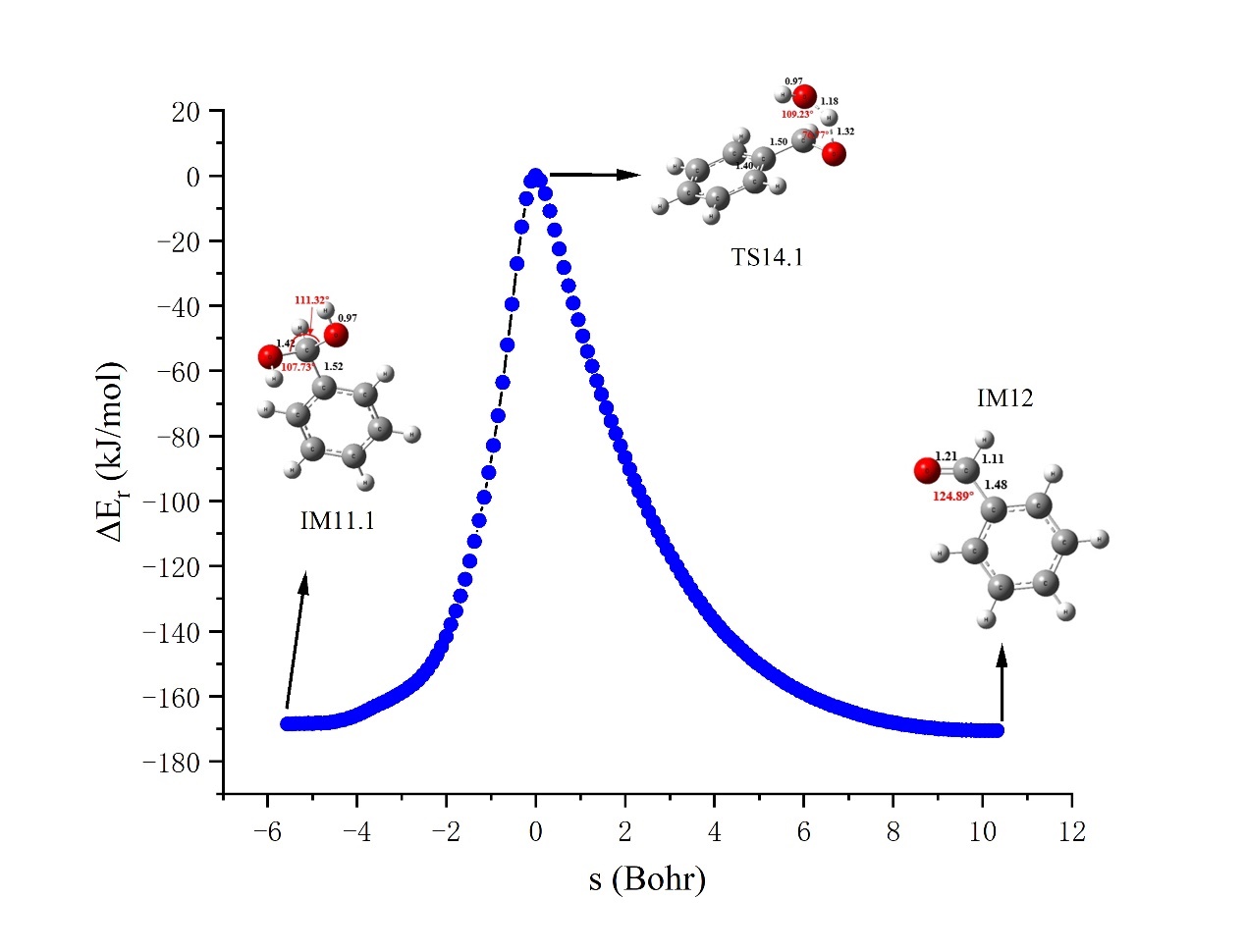

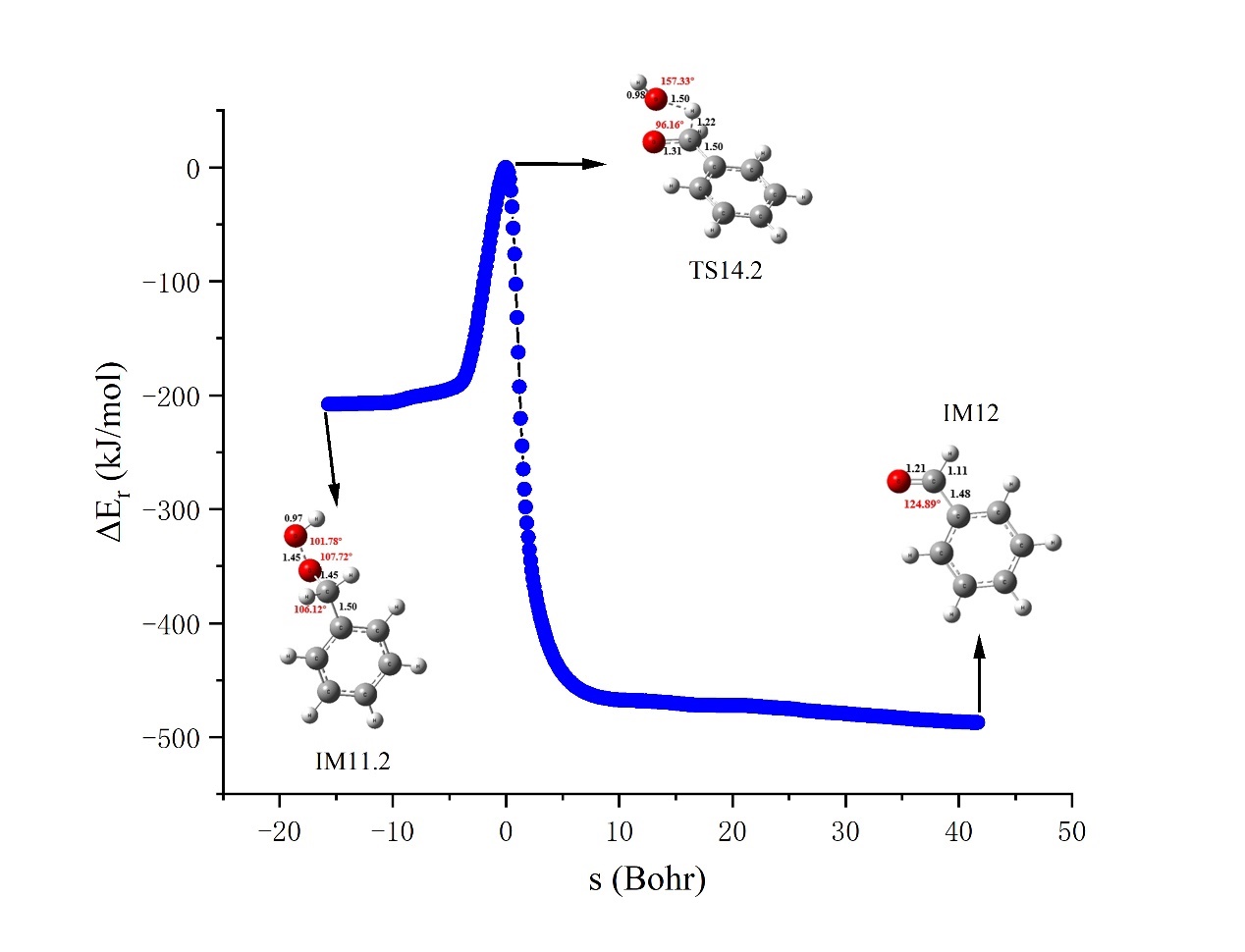

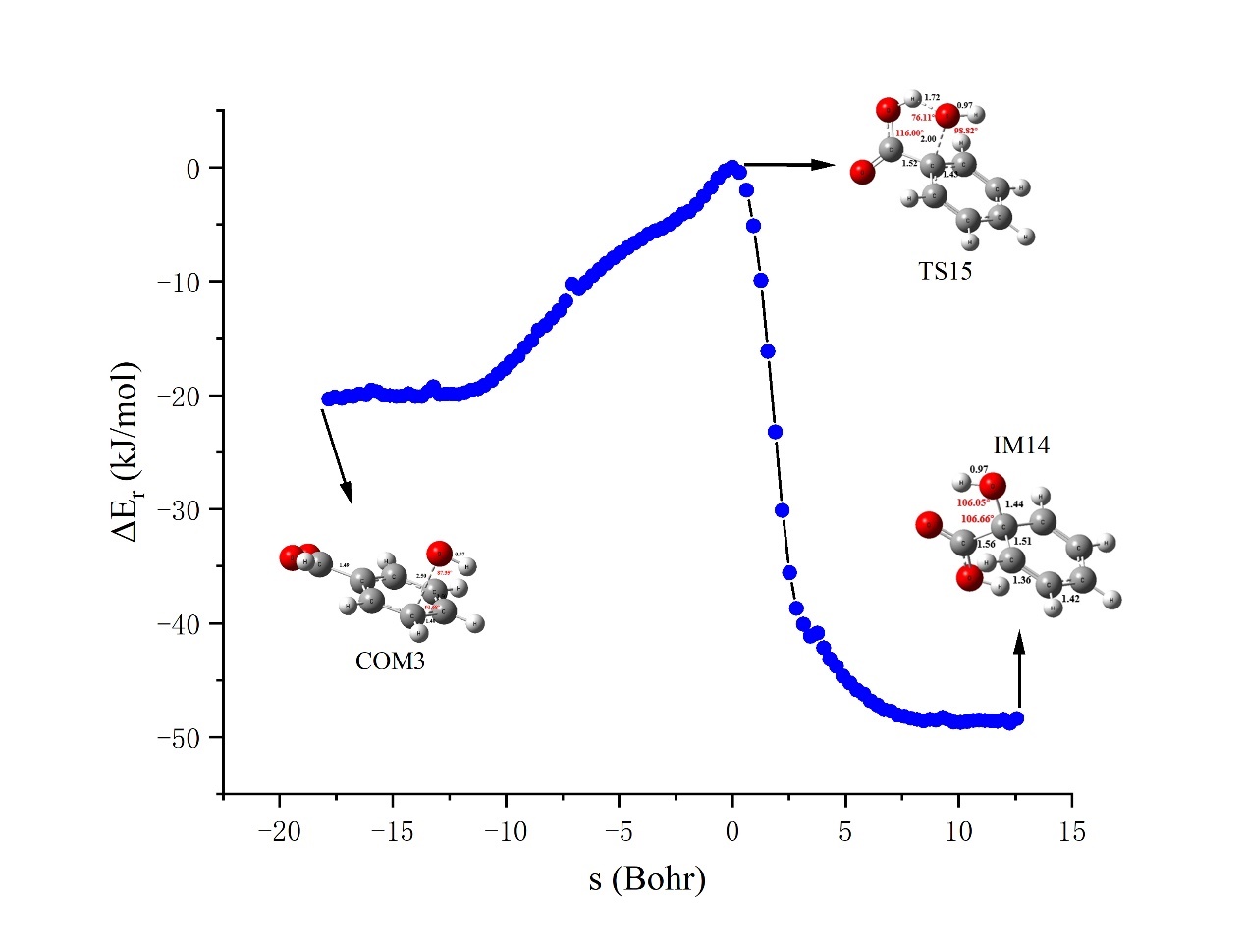

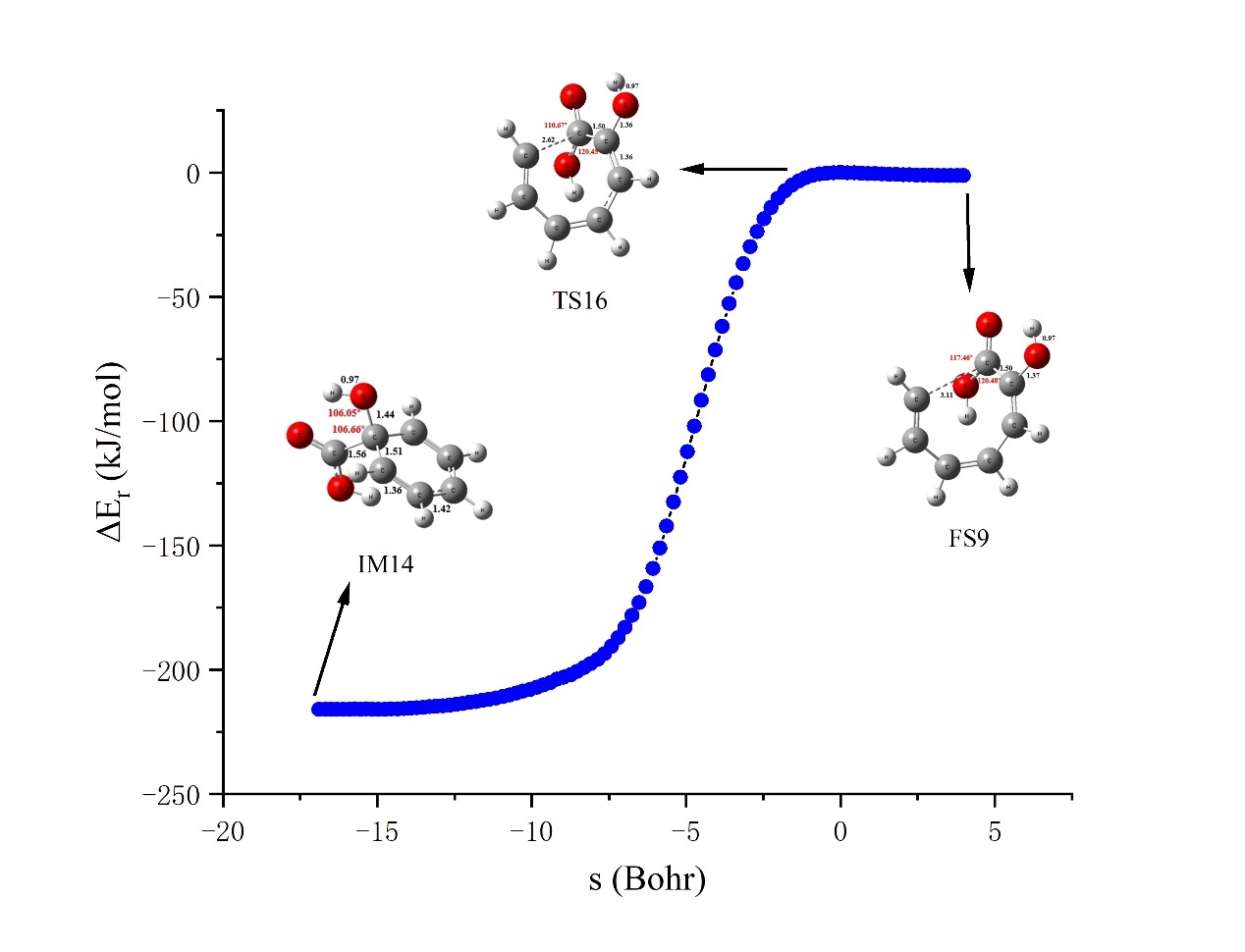

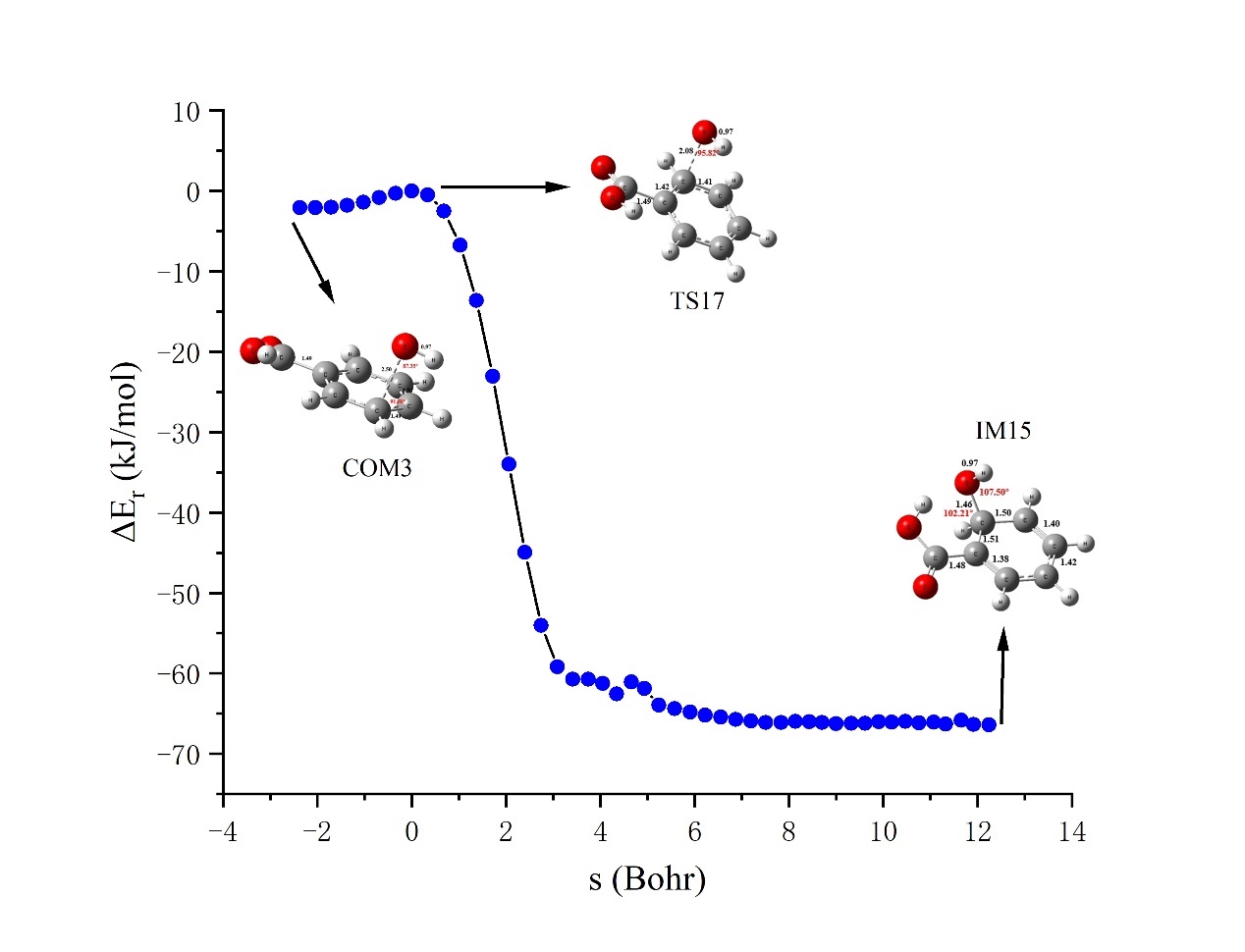

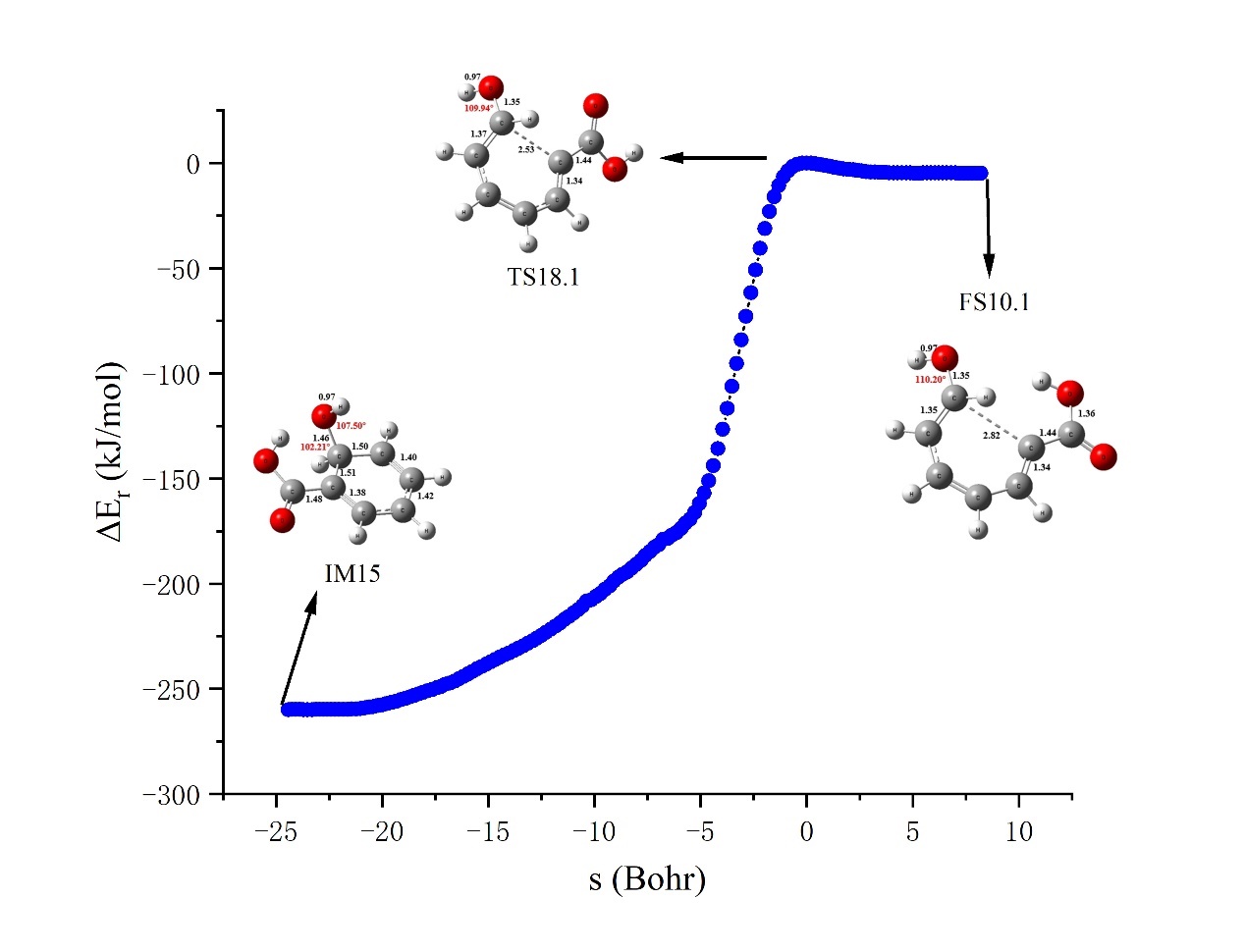

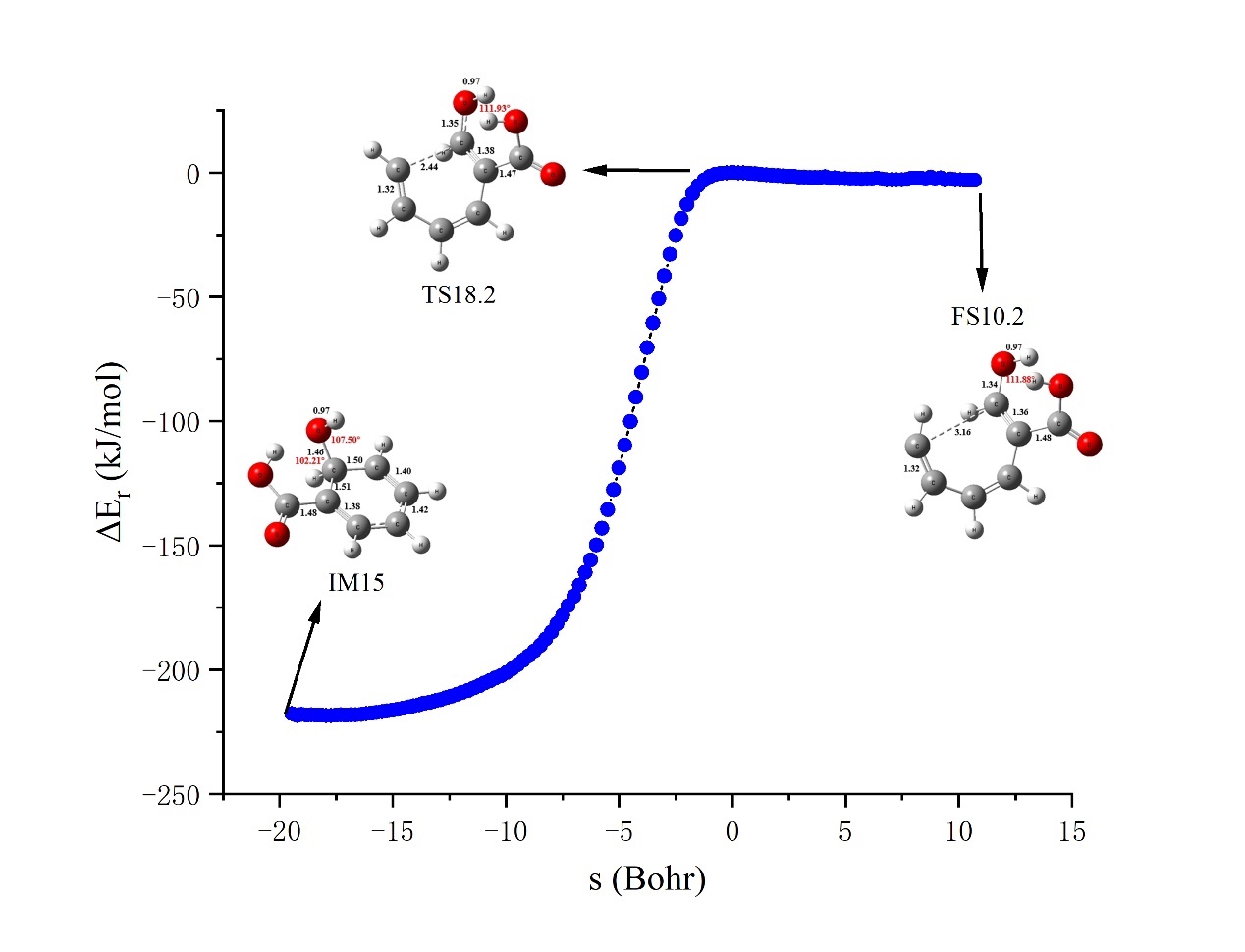

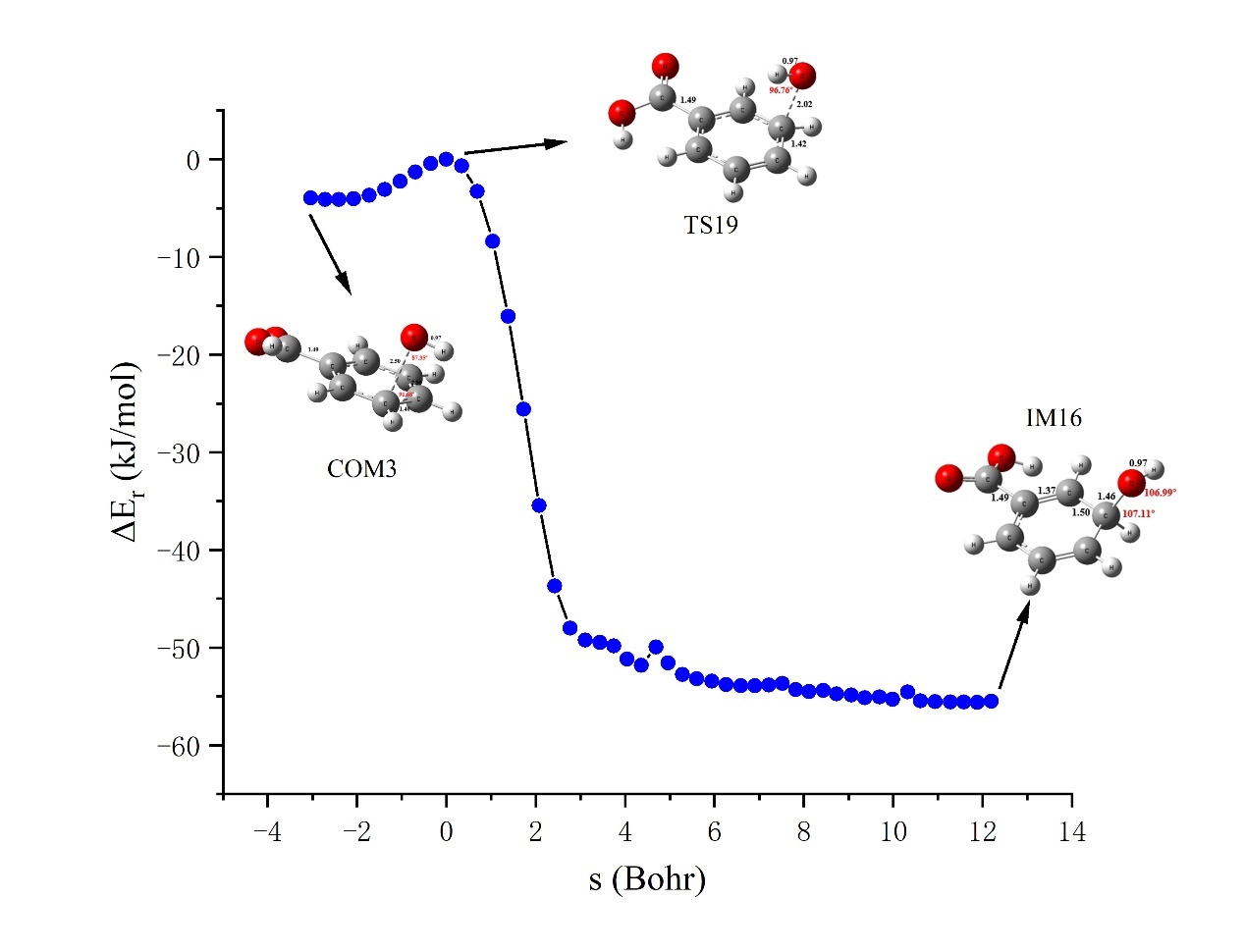

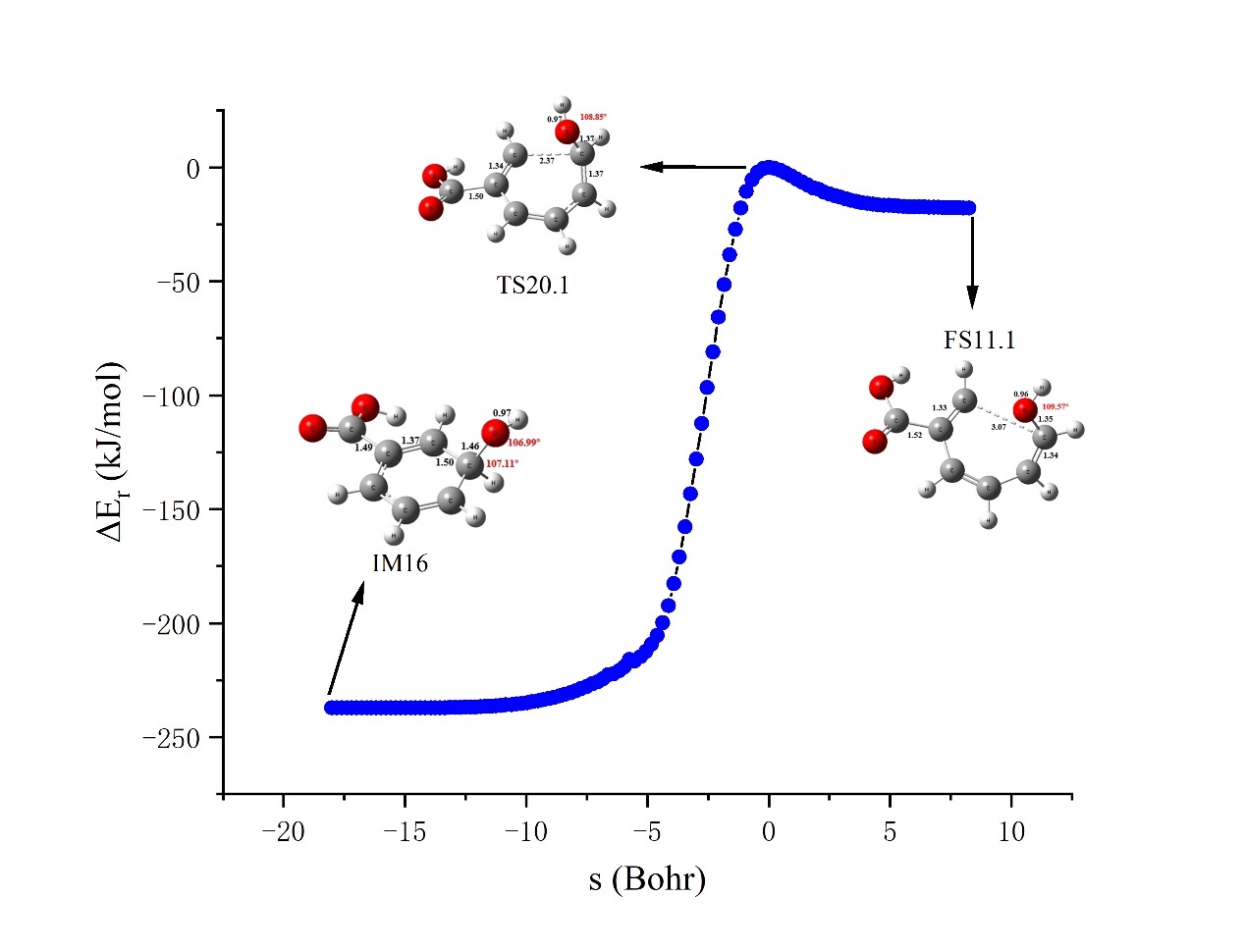

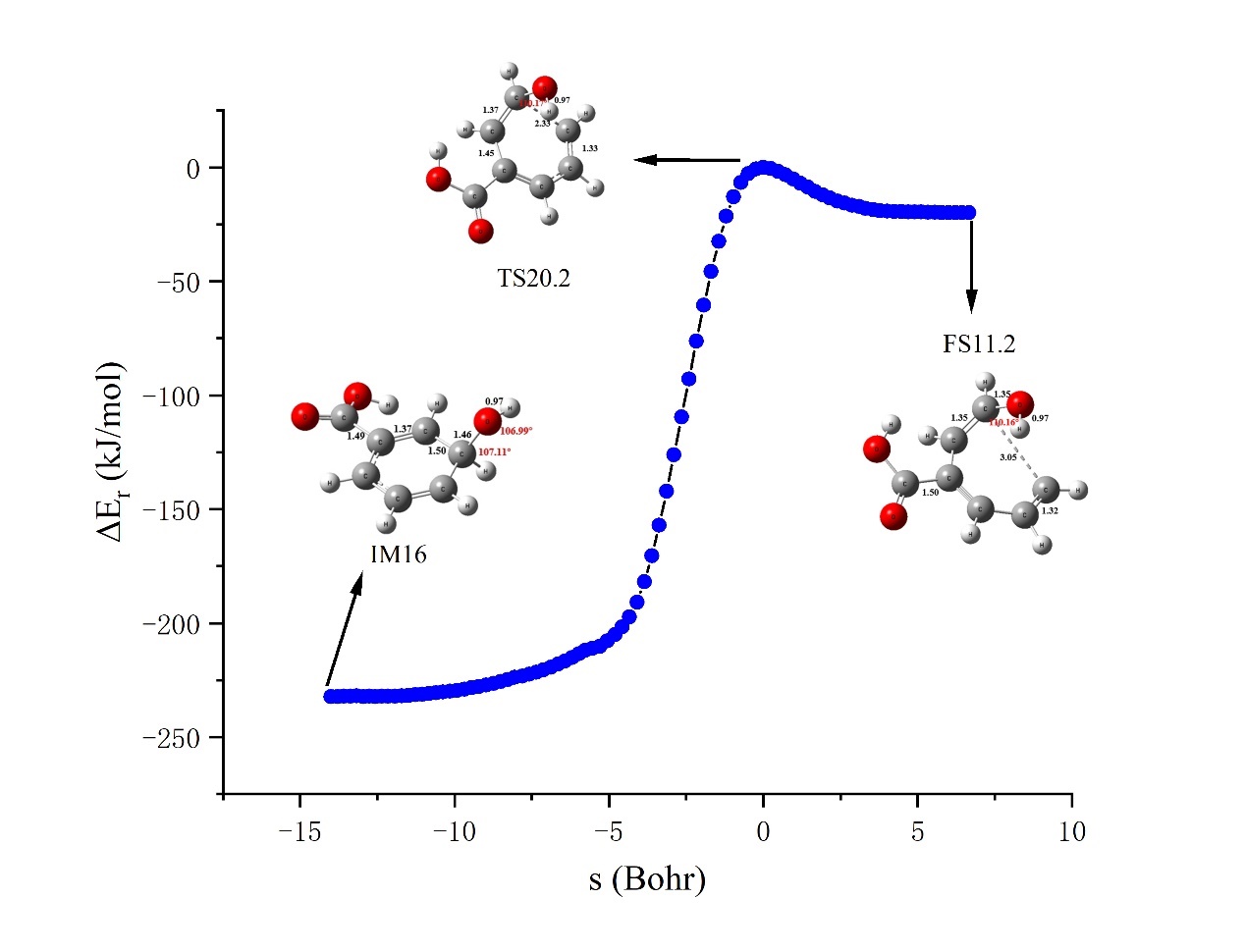

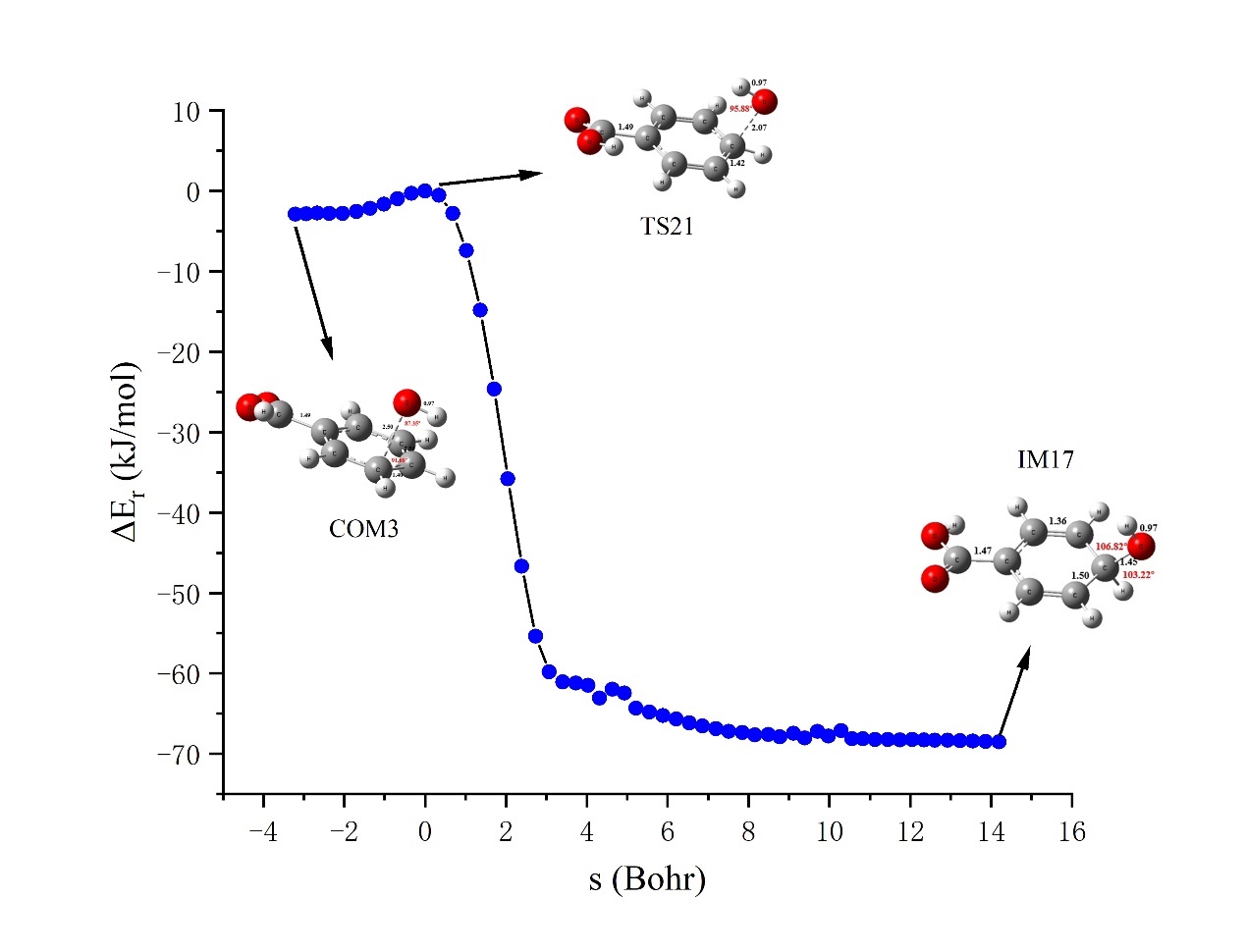

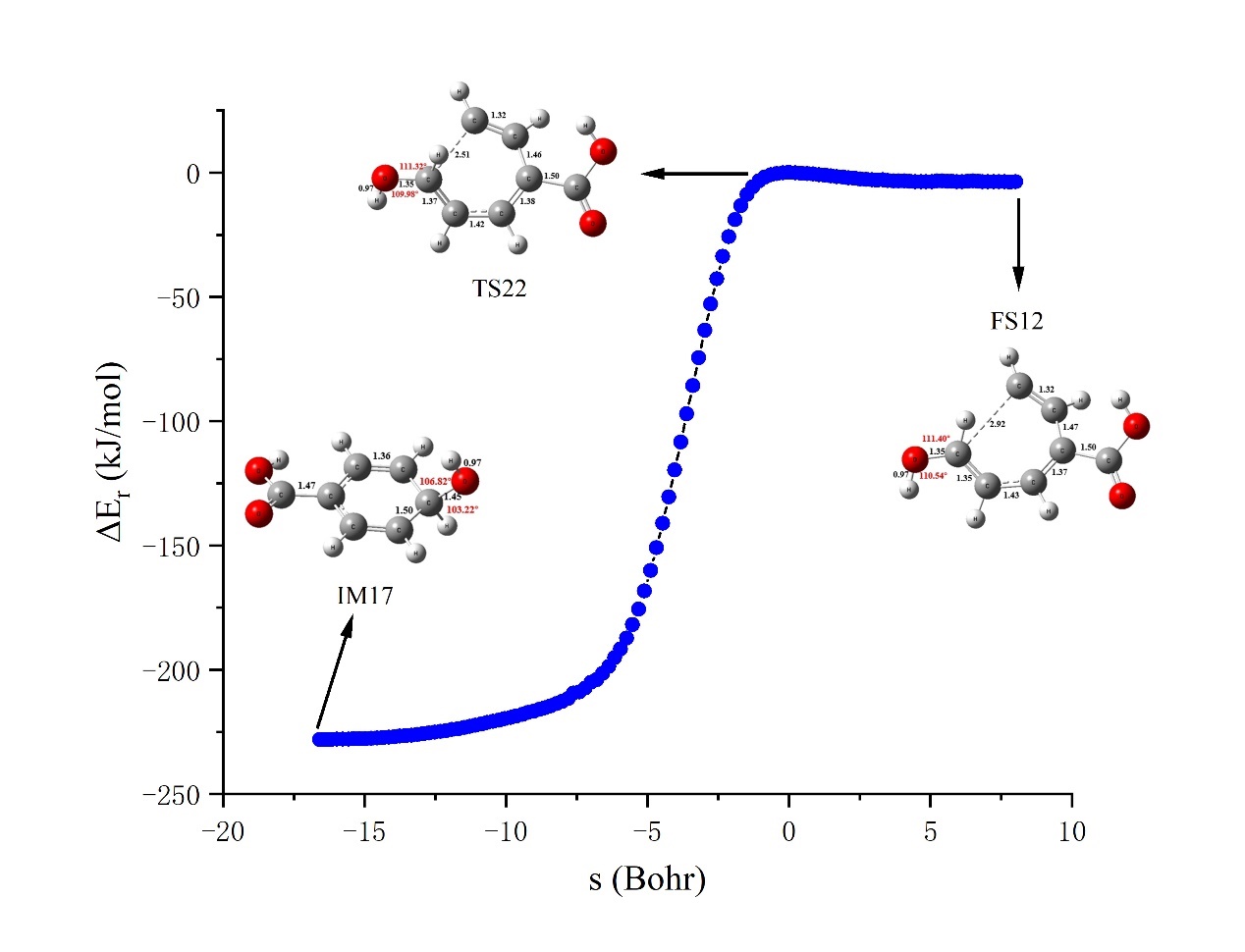


# Figure S4(a): Comprehensive Reaction Rate Constant Fitting at a Reaction Temperature of 25°C.


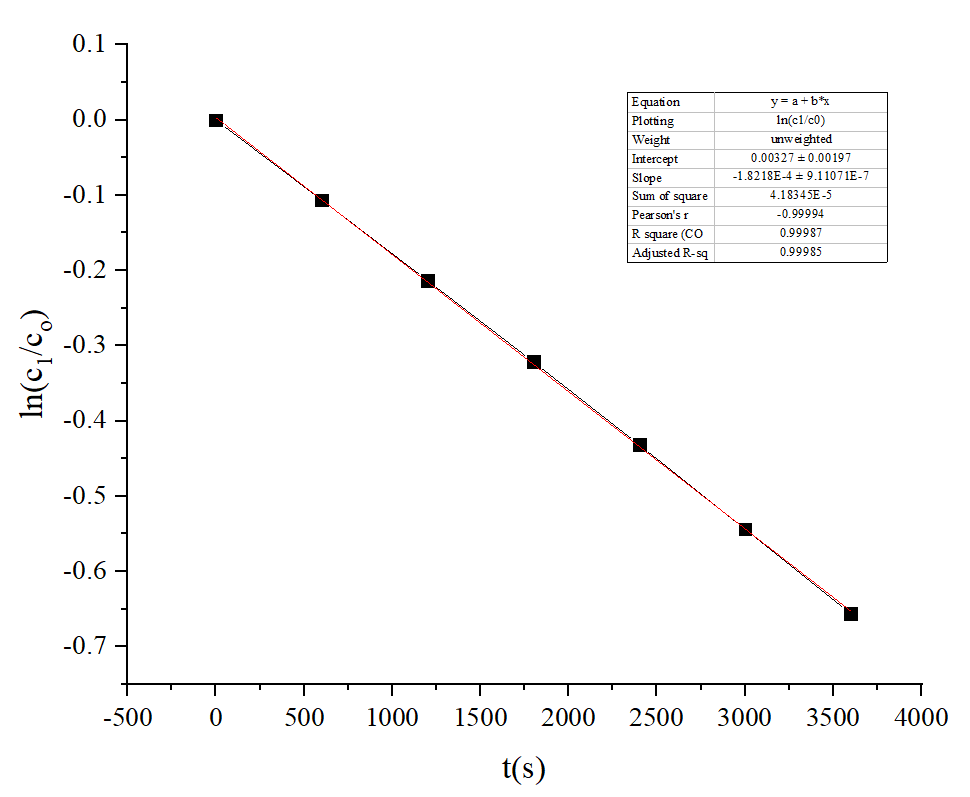


# Figure S4(b): Comprehensive Reaction Rate Constant Fitting at a Reaction Temperature of 40°C.


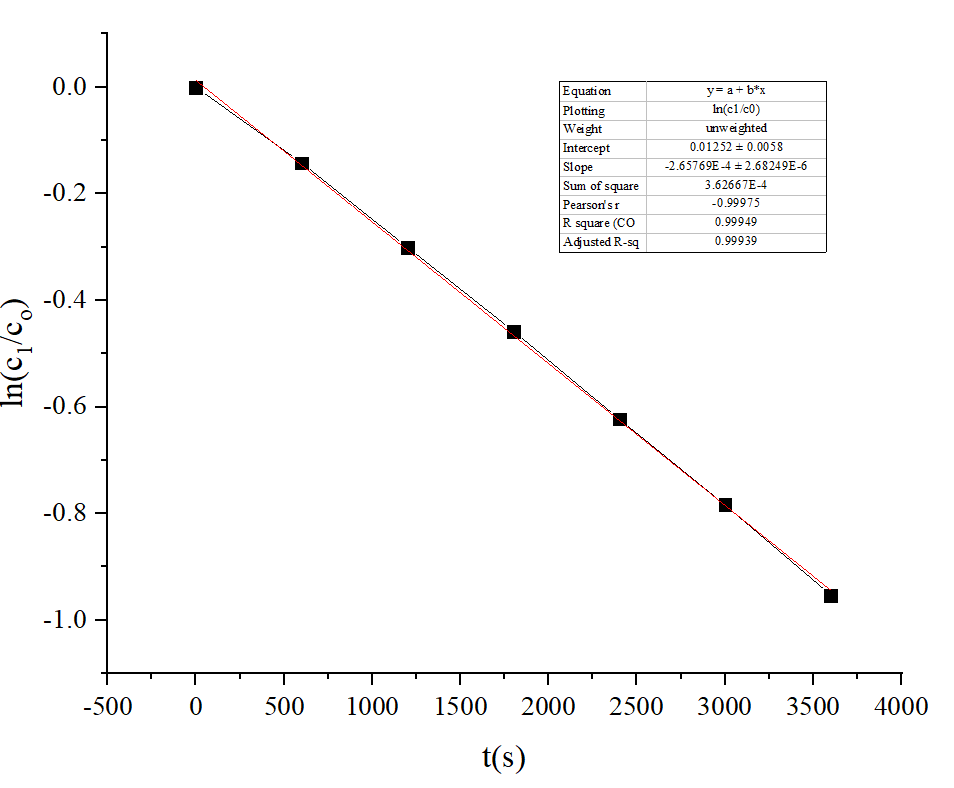


# Figure S4(c): Comprehensive Reaction Rate Constant Fitting at a Reaction Temperature of 50°C.


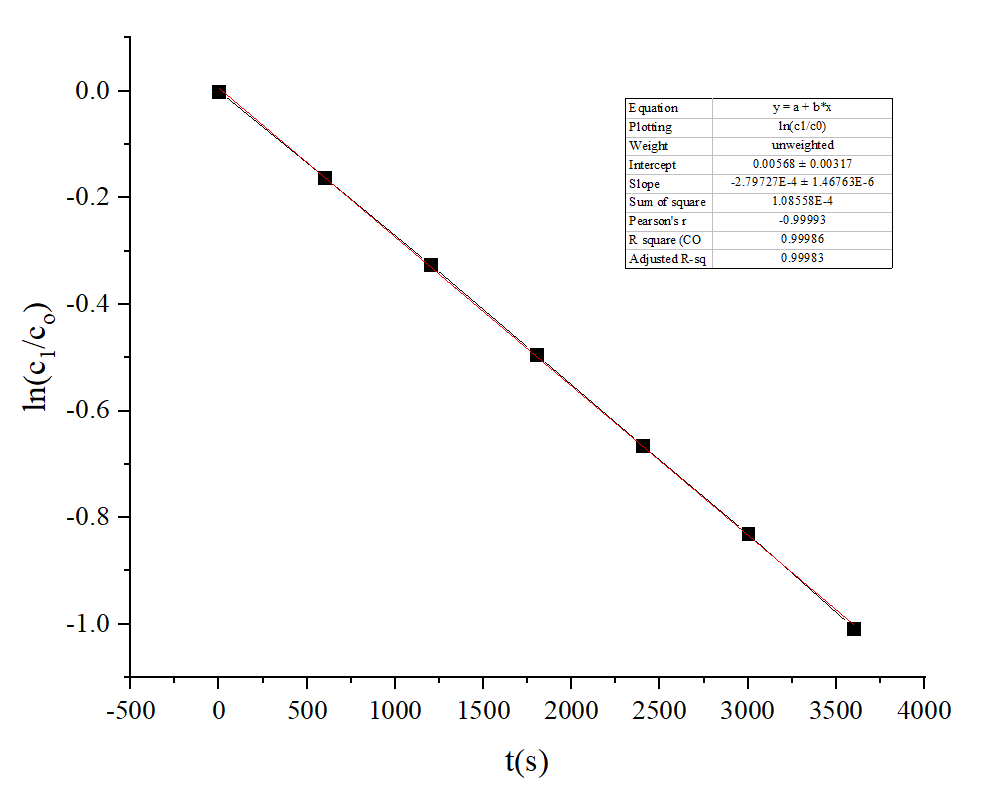


# Figure S4(d): Comprehensive Reaction Rate Constant Fitting at a Reaction Temperature of 60°C.


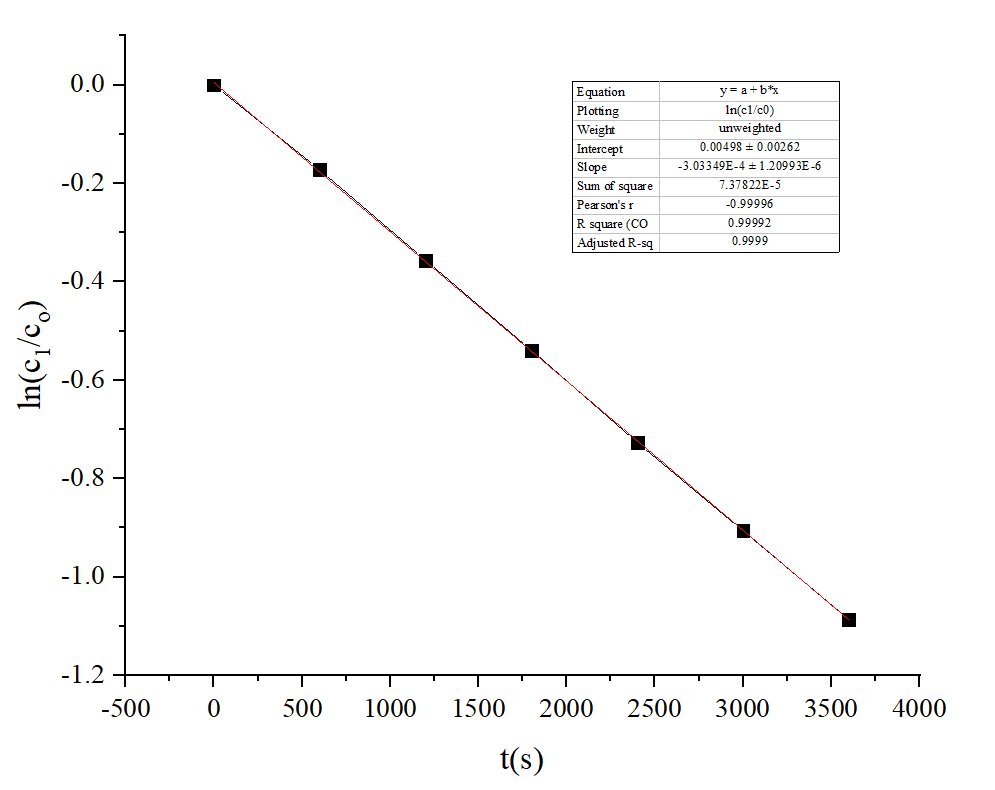


# Figure S4(e): Comprehensive Reaction Rate Constant Fitting at a Reaction Temperature of 70°C.


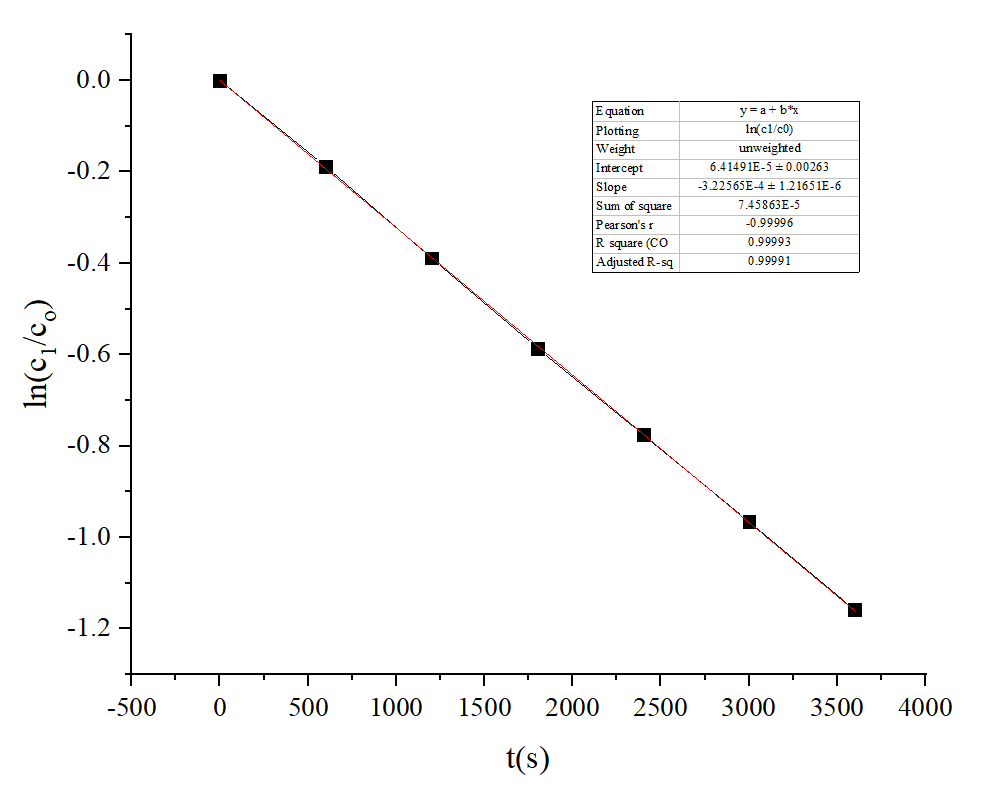


# Figure S5: Fitting of the Experimental Comprehensive Reaction Arrhenius Equation.


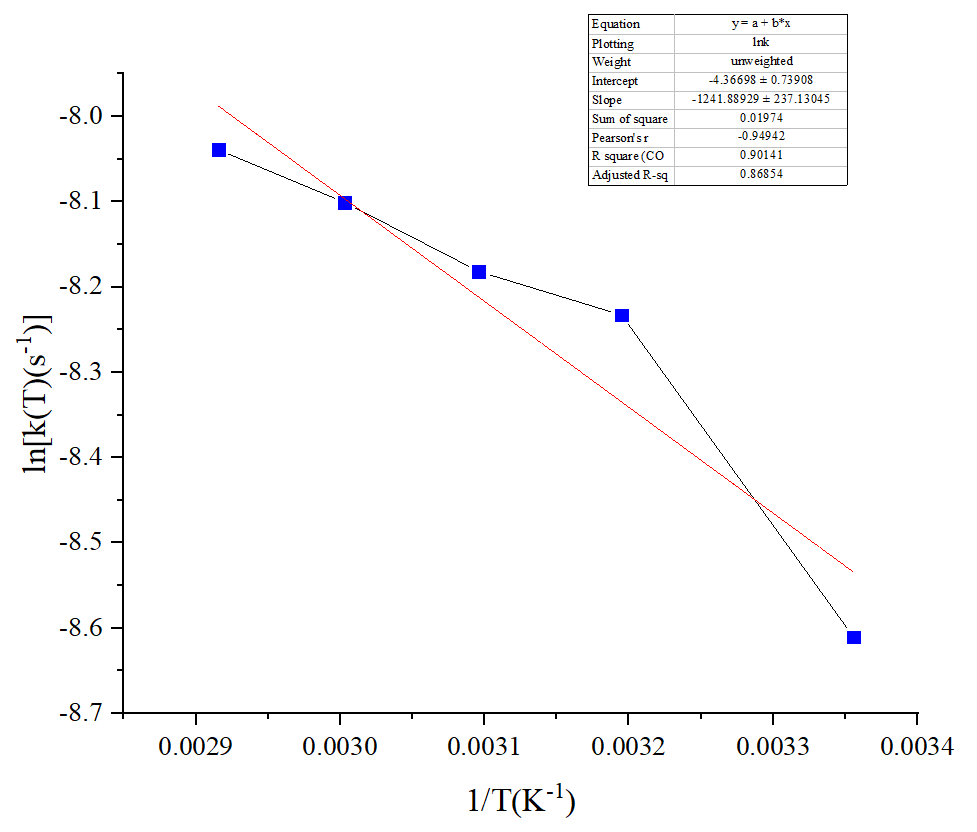


# Figure S6 (a) Arrhenius Equation Fitting for the Reaction IS+·OH→IM1+H_2_O


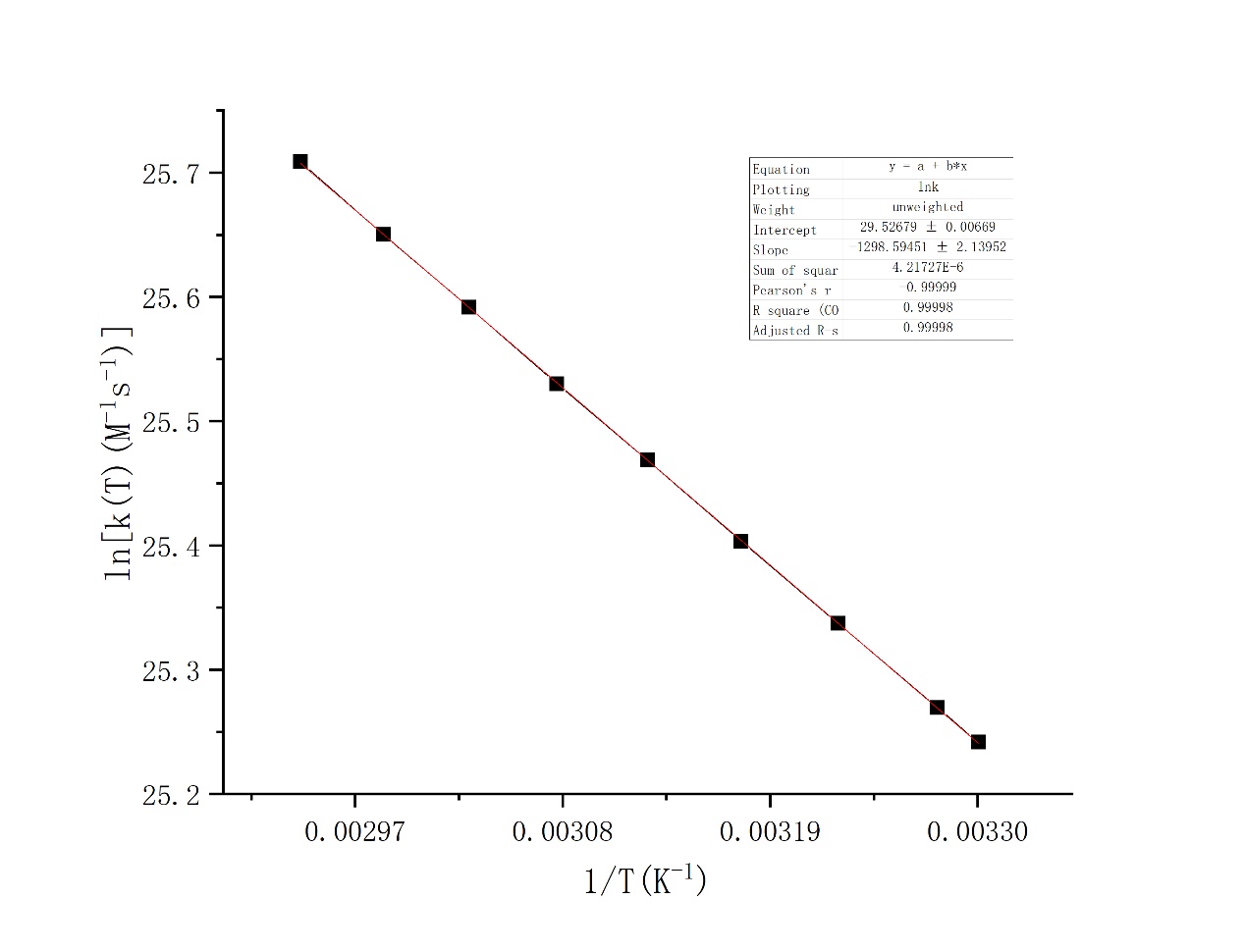
Figure S6 (b) Arrhenius Equation Fitting for the Reaction IS+·OH→IM2+H2O


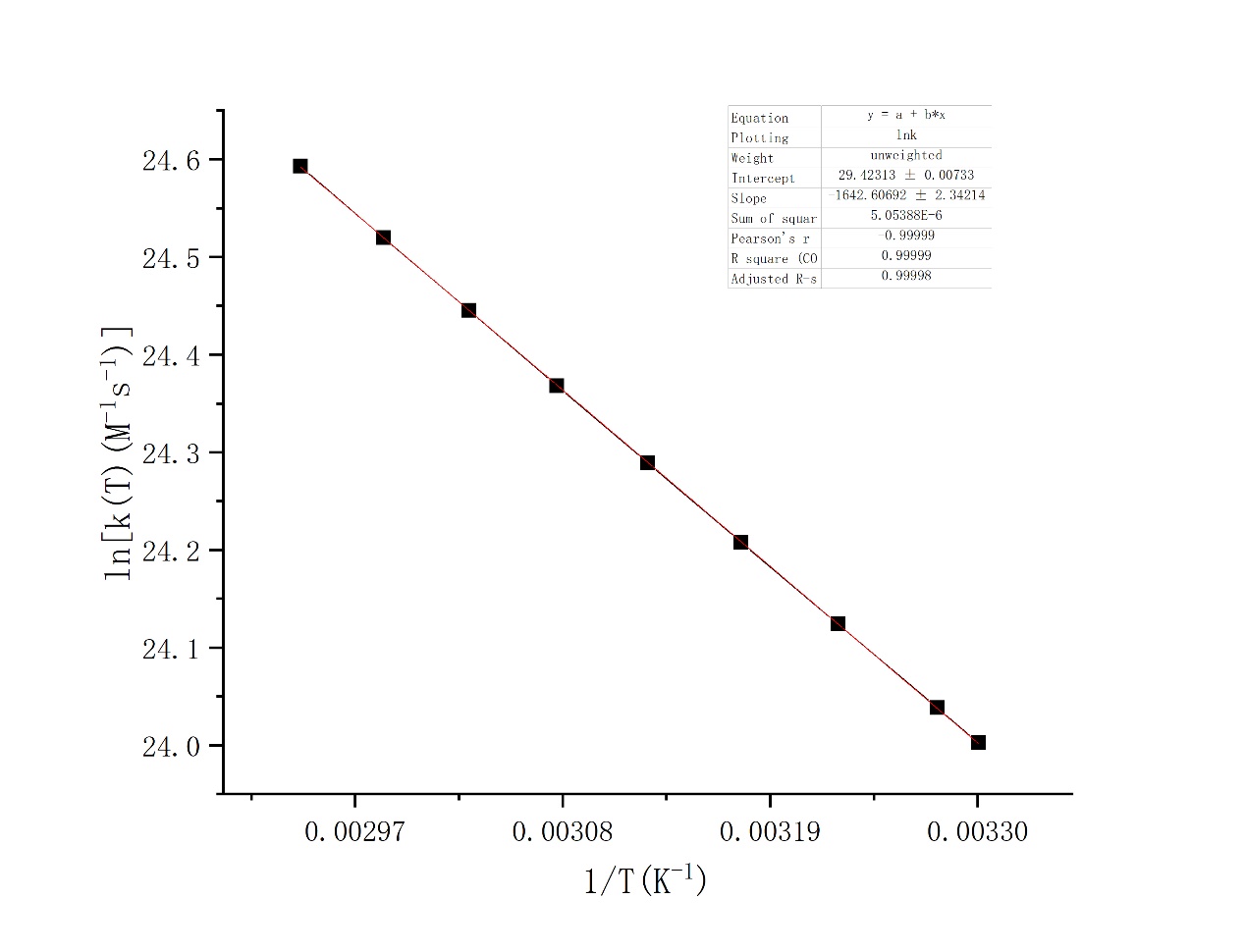


# Figure S6 (c) Arrhenius Equation Fitting for the Reaction IS+·OH→IM6


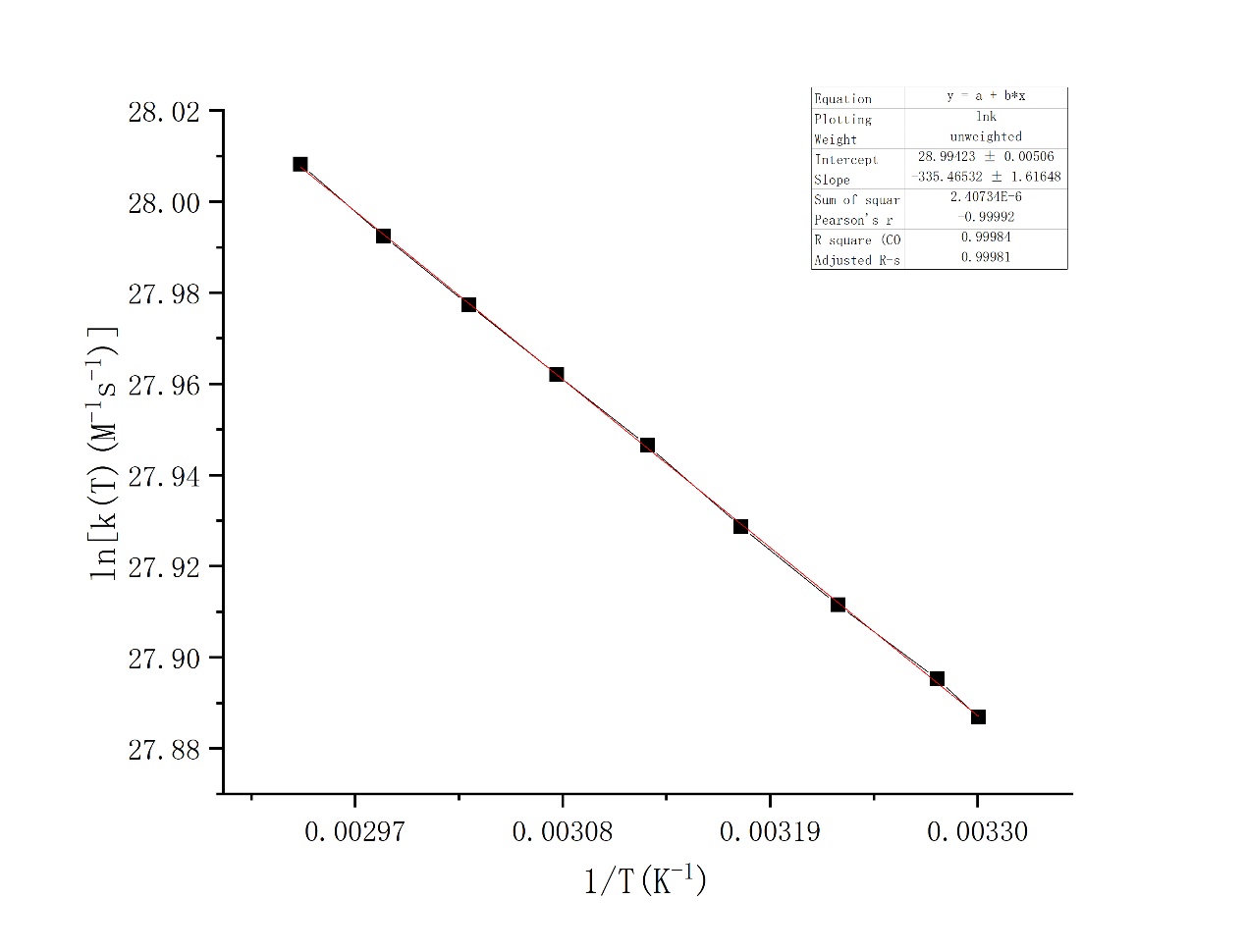
Figure S6 (d) Arrhenius Equation Fitting for the Reaction IS+·OH→IM7


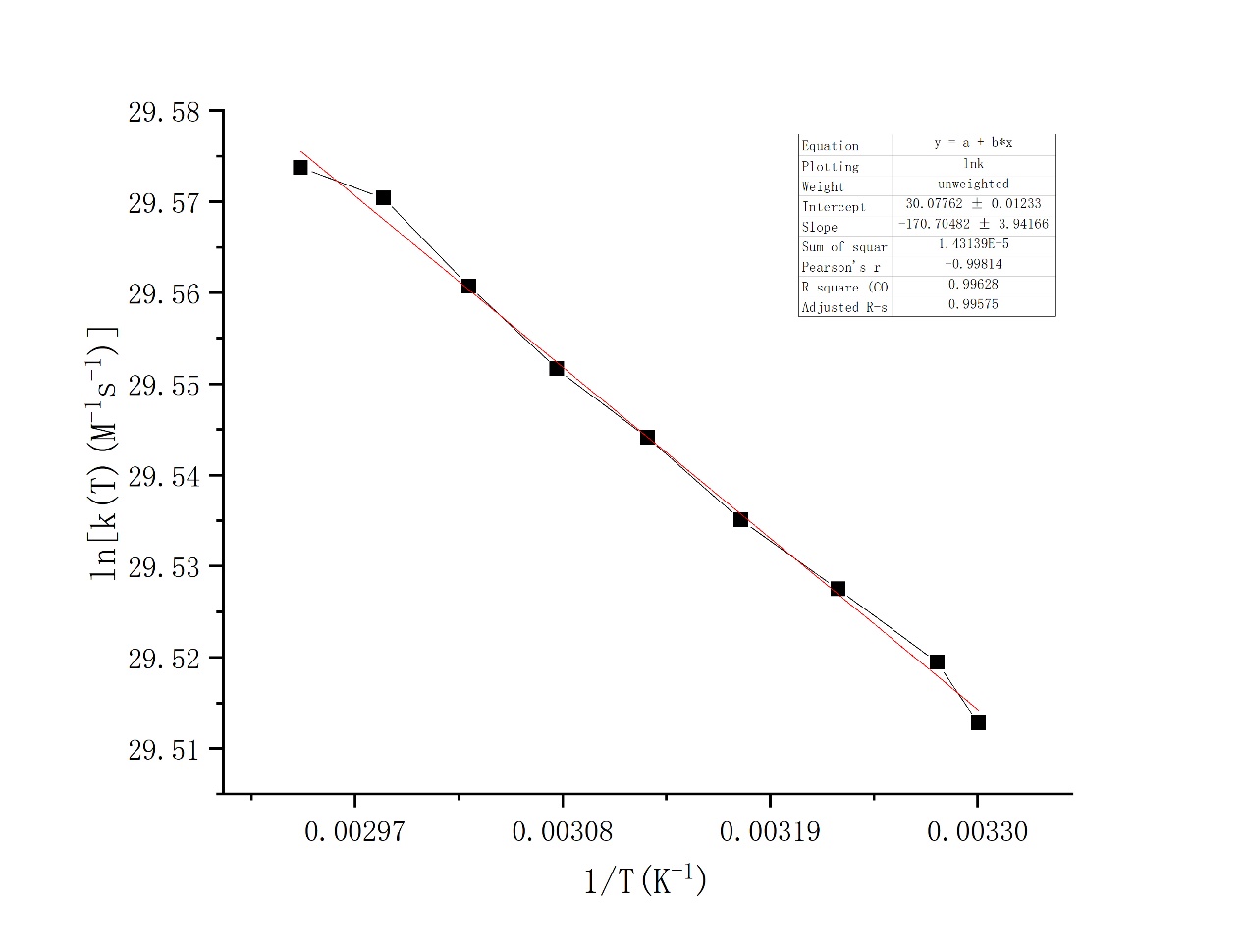


# Figure S6 (e) Arrhenius Equation Fitting for the Reaction IS+·OH→IM8+H_2_O


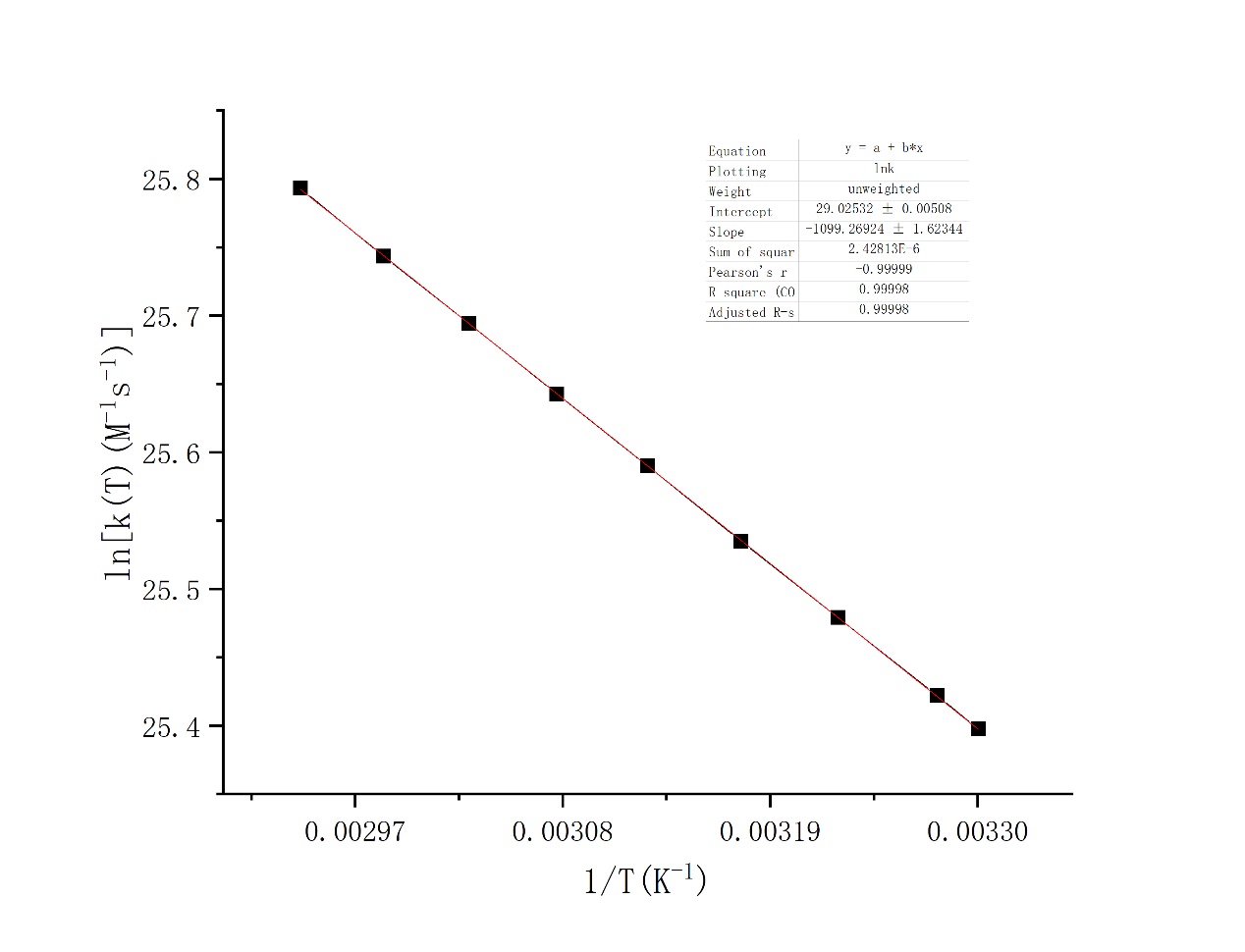


# Figure S6 (f) Arrhenius Equation Fitting for the Reaction FS8+·OH→IM14


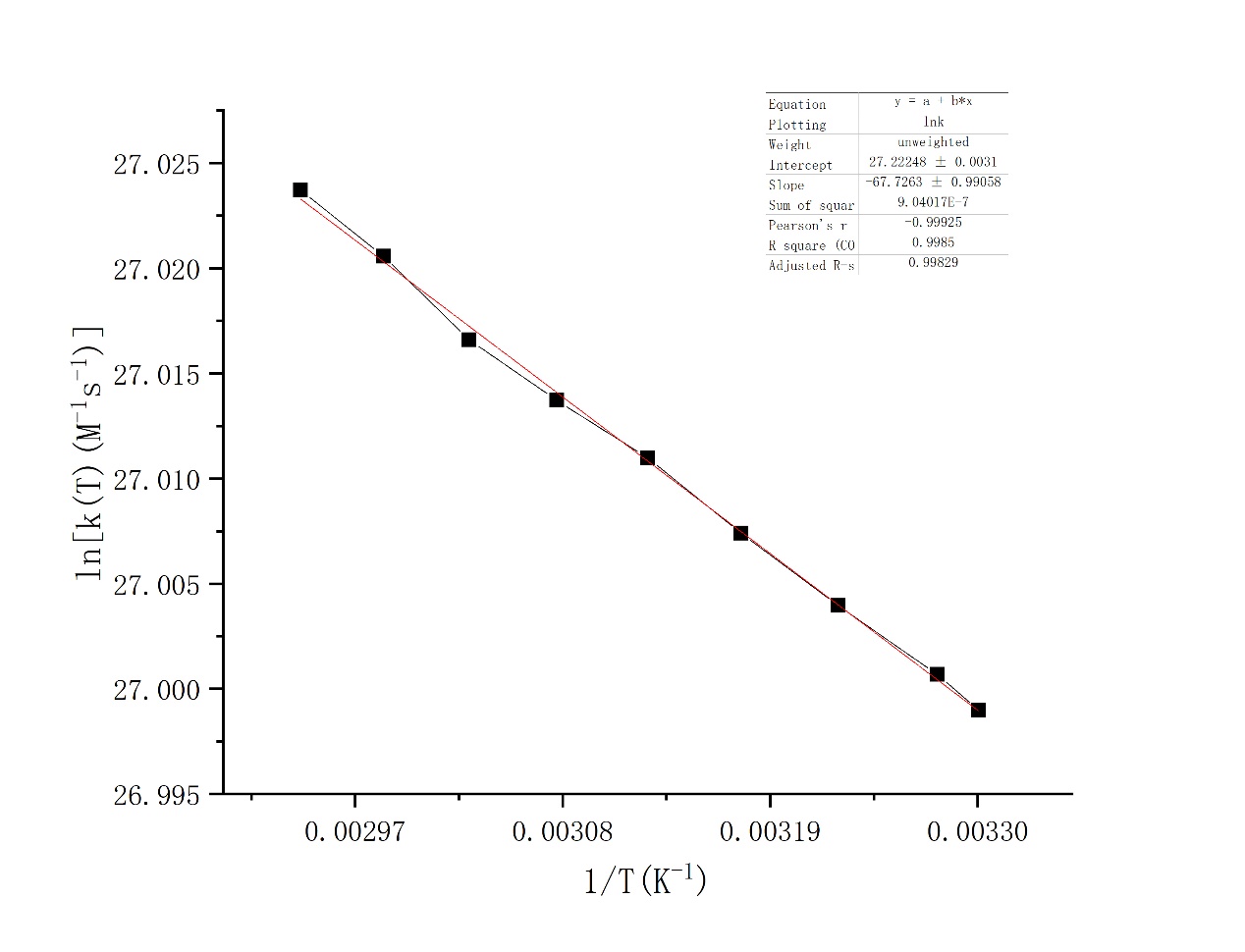


# Figure S6 (g) Arrhenius Equation Fitting for the Reaction FS8+·OH→IM15


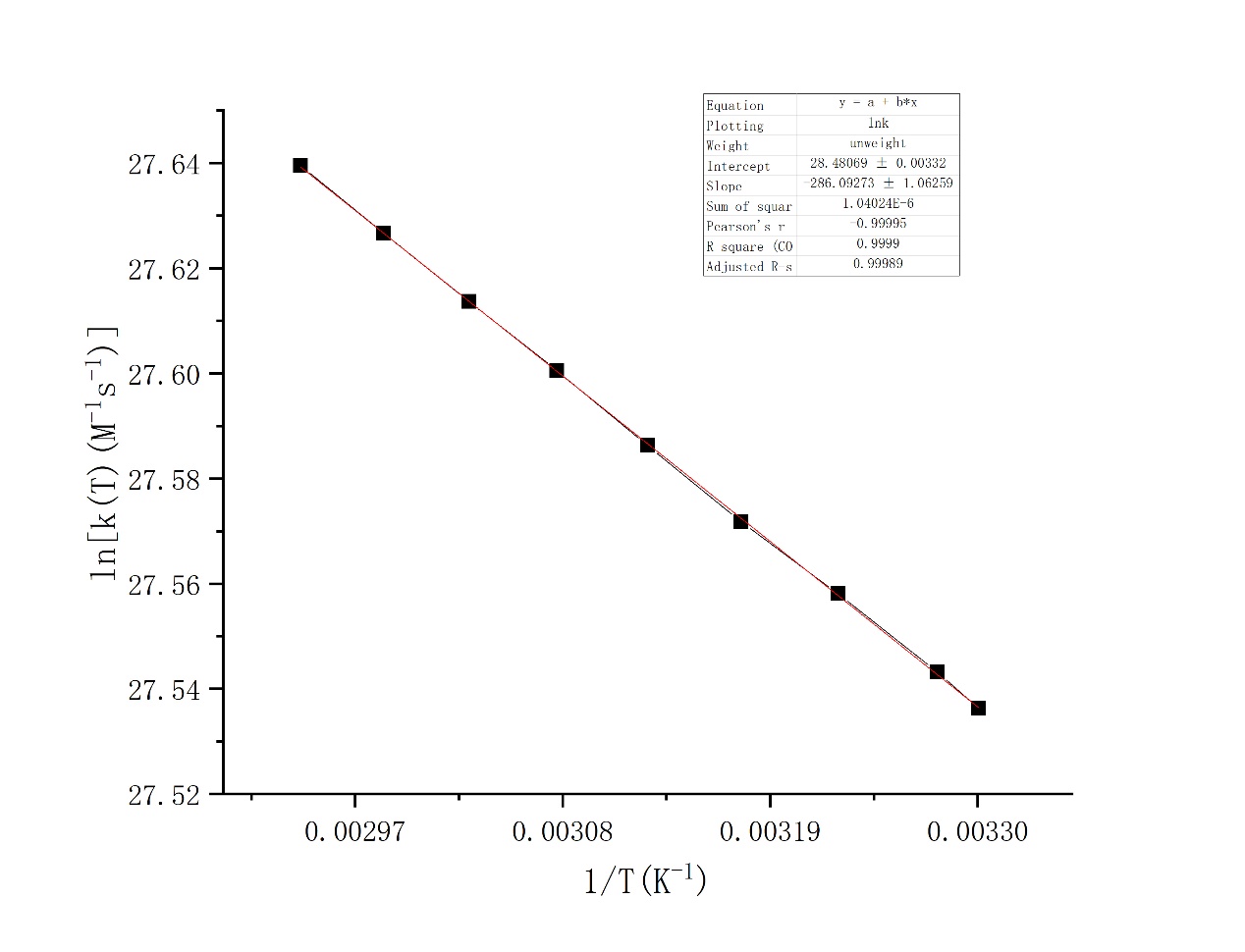


# Table S1 T1 Diagnostic Values for All Species in the Reaction under CCSD/cc-pVDZ

| Closed-shell Species | | Open-shell Species | | | | | |
| --- | --- | --- | --- | --- | --- | --- | --- |
| Species | T1 | Species | T1 | Species | T1 | Species | T1 |
| IS | 0.0095507 | COM1 | 0.0189224 | TS9.2 | 0.0420084 | TS16 | 0.0409235 |
| ·OH | 0.0055461 | TS1 | 0.0207513 | FS6.1 | 0.0420337 | FS9 | 0.0412709 |
| H_2_O | 0.0056632 | IM1 | 0.0415251 | FS6.2 | 0.0413679 | TS17 | 0.0345542 |
| FS1 | 0.0107212 | TS2 | 0.0207991 | TS10 | 0.0364242 | IM15 | 0.0287186 |
| FS2 | 0.0110817 | IM2 | 0.0415141 | IM7 | 0.0291566 | TS18.1 | 0.0391019 |
| FS3 | 0.0106374 | TS3 | 0.0210146 | TS11 | 0.0420578 | TS18.2 | 0.0404233 |
| IM9 | 0.0097637 | IM3 | 0.0416591 | FS7 | 0.0417174 | FS10.1 | 0.0408215 |
| IM11.1 | 0.0104769 | TS4 | 0.0365565 | TS12 | 0.0105997 | FS10.2 | 0.0371278 |
| IM11.2 | 0.0106228 | IM4 | 0.0290666 | IM8 | 0.0367456 | TS19 | 0.0345634 |
| IM12 | 0.0140949 | TS5 | 0.0431781 | COM2.1 | 0.0096635 | IM16 | 0.0285984 |
| FS8 | 0.014213 | FS4 | 0.0418054 | TS13.1 | 0.0114193 | TS20.1 | 0.0406651 |
| COM3 | 0.0166591 | TS6 | 0.0364479 | IM10.1 | 0.0352565 | TS20.2 | 0.0401268 |
|  |  | IM5 | 0.0289449 | TS14.1 | 0.013155 | FS11.1 | 0.0397311 |
|  |  | TS7.1 | 0.0418895 | COM2.2 | 0.0344149 | FS11.2 | 0.0374561 |
|  |  | TS7.2 | 0.0425023 | TS13.2 | 0.0308824 | TS21 | 0.034172 |
|  |  | FS5.1 | 0.0426419 | IM10.2 | 0.014636 | IM17 | 0.0283433 |
|  |  | FS5.2 | 0.0421397 | TS14.2 | 0.0169019 | TS22 | 0.0393482 |
|  |  | TS8 | 0.0361949 | IM13 | 0.016493 | FS12 | 0.0387644 |
|  |  | IM6 | 0.0288982 | TS15 | 0.0354374 |  |  |
|  |  | TS9.1 | 0.0419472 | IM14 | 0.0283646 |  |  |

# Table S2(a) Experimental instrument for degradation of toluene and model thereof

| Number | Instrument Name | Instrument Model | Instrument Manufacturer |
| --- | --- | --- | --- |
| 1 | Gas chromatograph | GC 9570 | Zhejiang Fuli Analytical Instrument Co., Ltd |
| 2 | pH meter | PHS-3C | Shanghai INESA Scientific Instrument Co., Ltd |
| 3 | Electric constant temperature water bath | DK-98-1 | Tianjin Teste Instrument Co., Ltd |
| 4 | Electronic balance | FR124CN | Shanghai OHAUS Instrument Co., Ltd |
| 5 | Hydrogen generator | HGZ-300 | Shandong Huifen Instrument Co., Ltd |
| 6 | Gas Flow Indicator | D08-4F | Beijing Qixing Huachuang Flow Meter Co., Ltd |
| 7 | Gas Flow Meter | D07 | Beijing Qixing Huachuang Flow Meter Co., Ltd |
| 8 | Electron Paramagnetic Resonance | MS-5000 | Magnettech |
| 9 | UV lamp | ZW10D | Yiwu Jiacheng lighting appliances |

# Table S2(b) Experimental Reagent specifications and supplier for degradation of toluene

| Number | Reagent name | Molecular formula | Molecular weight | specification | supplier |
| --- | --- | --- | --- | --- | --- |
| 1 | Potassium bisulfate | K_5_H_3_S_4_O_18_ | 614.76 | 42%~46% KHSO_5_ | McLean chemical reagent |
| 2 | Sodium persulfate | Na_2_S_2_O_8_ | 238.1 | ≥99%，Analytically pure | McLean chemical reagent |
| 3 | Phosphoric acid | H_3_PO_4_ | 98.00 | ≥85%，Analytically pure | Tianjin Beichen Fangzheng reagent |
| 4 | Sodium hydroxide | NaOH | 40 | ≥95%，Analytically pure | McLean chemical reagent |
| 5 | Hydrogen peroxide | H_2_O_2_ | 34.01 | 30%，Analytically pure | Tianjin Beichen Fangzheng reagent |
| 6 | Sodium hypochlorite | NaClO | 74.44 | Available chlorine≥30% | McLean chemical reagent |
| 7 | Toluene | C_7_H_8_ | 92.14 | ≥99.5%，Analytically pure | Sinopharm Group chemical reagents |
| 8 | Tert-butanol | C_4_H_10_O | 74.12 | ≥99.5%，Analytically pure | Tianjin City Damao chemical reagent |
| 9 | Anhydrous ethanol | C_2_H_6_O | 46.07 | ≥99.5%，Analytically pure | Tianjin City Damao chemical reagent |
| 10 | Furfuryl alcohol | C_5_H_6_O_2_ | 98.10 | ≥98%，Analytically pure | McLean chemical reagent |

# Table S3(a): Absolute Energy Data for All Species Calculated Using the B3LYP/6-311++G(d,p) Computational Scheme.

| Species | Zero-point correction | Thermal correction to Energy | Thermal correction to Enthalpy | Thermal correction to Gibbs Free Energy | Sum of electronic and thermal Energies | Sum of electronic and thermal Energies | Sum of electronic and thermal Enthalpies | Sum of electronic and thermal Free Energies |
| --- | --- | --- | --- | --- | --- | --- | --- | --- |
| IS | 0.127362 | 0.133483 | 0.134427 | 0.097374 | -271.5133 | -271.5071 | -271.5062 | -271.5433 |
| ·OH | 0.008413 | 0.010773 | 0.011717 | -0.008517 | -75.75574 | -75.75338 | -75.75244 | -75.77267 |
| H_2_O | 0.021093 | 0.023928 | 0.024873 | 0.003436 | -76.44006 | -76.43723 | -76.43628 | -76.45772 |
| COM1 | 0.138375 | 0.147358 | 0.148302 | 0.103778 | -347.2736 | -347.2646 | -347.2637 | -347.3082 |
| TS1 | 0.132737 | 0.141067 | 0.142011 | 0.09918 | -347.2699 | -347.2616 | -347.2606 | -347.3035 |
| IM1 | 0.114384 | 0.120526 | 0.12147 | 0.083642 | -270.8376 | -270.8314 | -270.8305 | -270.8683 |
| FS1 | 0.131435 | 0.138564 | 0.139508 | 0.100526 | -346.7613 | -346.7542 | -346.7532 | -346.7922 |
| TS2 | 0.132594 | 0.14104 | 0.141984 | 0.09863 | -347.269 | -347.2605 | -347.2596 | -347.3029 |
| IM2 | 0.114313 | 0.120392 | 0.121336 | 0.083839 | -270.8375 | -270.8314 | -270.8304 | -270.8679 |
| FS2 | 0.131411 | 0.138566 | 0.139511 | 0.100352 | -346.7609 | -346.7537 | -346.7528 | -346.7919 |
| TS3 | 0.132155 | 0.140824 | 0.141768 | 0.097445 | -347.2689 | -347.2603 | -347.2593 | -347.3037 |
| IM3 | 0.11393 | 0.119298 | 0.120242 | 0.084373 | -270.8369 | -270.8315 | -270.8306 | -270.8665 |
| FS3 | 0.131145 | 0.138517 | 0.139461 | 0.09936 | -346.7602 | -346.7528 | -346.7519 | -346.792 |
| TS4 | 0.138503 | 0.146405 | 0.147349 | 0.106019 | -347.2727 | -347.2648 | -347.2638 | -347.3052 |
| IM4 | 0.140136 | 0.147836 | 0.14878 | 0.10797 | -347.2915 | -347.2838 | -347.2828 | -347.3236 |
| TS5 | 0.136003 | 0.14409 | 0.145034 | 0.103589 | -347.2065 | -347.1984 | -347.1975 | -347.2389 |
| FS4 | 0.137144 | 0.146123 | 0.147067 | 0.103206 | -347.2162 | -347.2073 | -347.2063 | -347.2502 |
| TS6 | 0.138514 | 0.146555 | 0.147499 | 0.105469 | -347.2748 | -347.2668 | -347.2658 | -347.3078 |
| IM5 | 0.140569 | 0.148409 | 0.149353 | 0.10771 | -347.293 | -347.2852 | -347.2842 | -347.3259 |
| TS7.1 | 0.136821 | 0.144956 | 0.1459 | 0.103902 | -347.2078 | -347.1997 | -347.1987 | -347.2407 |
| TS7.2 | 0.136587 | 0.144939 | 0.145883 | 0.103216 | -347.2167 | -347.2083 | -347.2074 | -347.2501 |
| FS5.1 | 0.137083 | 0.146175 | 0.147119 | 0.102643 | -347.2089 | -347.1998 | -347.1989 | -347.2433 |
| FS5.2 | 0.137803 | 0.147069 | 0.148013 | 0.102734 | -347.2195 | -347.2102 | -347.2093 | -347.2545 |
| TS8 | 0.138124 | 0.146418 | 0.147363 | 0.104432 | -347.2728 | -347.2645 | -347.2636 | -347.3065 |
| IM6 | 0.140536 | 0.148339 | 0.149283 | 0.108002 | -347.2906 | -347.2828 | -347.2819 | -347.3232 |
| TS9.1 | 0.136909 | 0.14498 | 0.145924 | 0.104078 | -347.2078 | -347.1997 | -347.1987 | -347.2406 |
| TS9.2 | 0.1366 | 0.144716 | 0.14566 | 0.103884 | -347.2087 | -347.2006 | -347.1996 | -347.2414 |
| FS6.1 | 0.137528 | 0.146652 | 0.147597 | 0.102612 | -347.2099 | -347.2008 | -347.1998 | -347.2448 |
| FS6.2 | 0.136674 | 0.14593 | 0.146874 | 0.10206 | -347.2107 | -347.2015 | -347.2005 | -347.2453 |
| TS10 | 0.138092 | 0.146419 | 0.147363 | 0.10432 | -347.2744 | -347.266 | -347.2651 | -347.3081 |
| IM7 | 0.140625 | 0.148588 | 0.149533 | 0.107197 | -347.2916 | -347.2837 | -347.2827 | -347.3251 |
| TS11 | 0.136116 | 0.144375 | 0.145319 | 0.103136 | -347.2088 | -347.2006 | -347.1996 | -347.2418 |
| FS7 | 0.13666 | 0.145892 | 0.146836 | 0.102133 | -347.21 | -347.2008 | -347.1998 | -347.2445 |
| TS12 | 0.137041 | 0.145226 | 0.14617 | 0.103184 | -347.2709 | -347.2627 | -347.2618 | -347.3047 |
| IM8 | 0.114119 | 0.119801 | 0.120746 | 0.084438 | -270.874 | -270.8684 | -270.8674 | -270.9037 |
| IM9 | 0.132708 | 0.139827 | 0.140771 | 0.101024 | -346.7471 | -346.74 | -346.739 | -346.7788 |
| COM2.1 | 0.144142 | 0.152728 | 0.153672 | 0.109578 | -422.5108 | -422.5022 | -422.5012 | -422.5453 |
| TS13.1 | 0.143169 | 0.152077 | 0.153021 | 0.107853 | -422.5066 | -422.4977 | -422.4968 | -422.5419 |
| IM10.1 | 0.119389 | 0.126245 | 0.12719 | 0.087899 | -346.1226 | -346.1157 | -346.1148 | -346.1541 |
| IM11.1 | 0.138089 | 0.14603 | 0.146975 | 0.105101 | -422.0004 | -421.9924 | -421.9915 | -422.0334 |
| TS14.1 | 0.131239 | 0.138986 | 0.13993 | 0.098371 | -421.9425 | -421.9347 | -421.9338 | -421.9753 |
| COM2.2 | 0.144414 | 0.15387 | 0.154814 | 0.109233 | -422.5126 | -422.5031 | -422.5022 | -422.5478 |
| TS13.2 | 0.139542 | 0.148465 | 0.149409 | 0.104201 | -422.5089 | -422.5 | -422.499 | -422.5442 |
| IM10.2 | 0.117649 | 0.124495 | 0.125439 | 0.085532 | -346.0906 | -346.0838 | -346.0828 | -346.1227 |
| IM11.2 | 0.136243 | 0.144479 | 0.145423 | 0.10263 | -421.9004 | -421.8922 | -421.8912 | -421.934 |
| TS14.2 | 0.128273 | 0.136537 | 0.137481 | 0.094842 | -421.8297 | -421.8215 | -421.8205 | -421.8632 |
| IM12 | 0.109649 | 0.115939 | 0.116884 | 0.079106 | -345.5639 | -345.5577 | -345.5567 | -345.5945 |
| IM13 | 0.097274 | 0.103569 | 0.104513 | 0.066088 | -344.9191 | -344.9128 | -344.9118 | -344.9502 |
| FS8 | 0.114844 | 0.121989 | 0.122934 | 0.082721 | -420.8393 | -420.8322 | -420.8312 | -420.8715 |
| COM3 | 0.125138 | 0.135506 | 0.13645 | 0.087337 | -496.5927 | -496.5823 | -496.5814 | -496.6305 |
| TS15 | 0.1259 | 0.134676 | 0.13562 | 0.09132 | -496.592 | -496.5832 | -496.5823 | -496.6266 |
| IM14 | 0.127503 | 0.136296 | 0.13724 | 0.093145 | -496.6066 | -496.5978 | -496.5969 | -496.641 |
| TS16 | 0.123384 | 0.132528 | 0.133472 | 0.088858 | -496.5283 | -496.5192 | -496.5182 | -496.5628 |
| FS9 | 0.124015 | 0.133961 | 0.134906 | 0.088254 | -496.5281 | -496.5182 | -496.5172 | -496.5639 |
| TS17 | 0.125795 | 0.135031 | 0.135975 | 0.090452 | -496.5921 | -496.5828 | -496.5819 | -496.6274 |
| IM15 | 0.129046 | 0.137412 | 0.138356 | 0.095152 | -496.6207 | -496.6124 | -496.6114 | -496.6546 |
| TS18.1 | 0.124536 | 0.133987 | 0.134931 | 0.088427 | -496.5399 | -496.5305 | -496.5295 | -496.576 |
| TS18.2 | 0.1243 | 0.133591 | 0.134535 | 0.088974 | -496.5264 | -496.5171 | -496.5161 | -496.5617 |
| FS10.1 | 0.125485 | 0.135689 | 0.136634 | 0.088197 | -496.5409 | -496.5307 | -496.5298 | -496.5782 |
| FS10.2 | 0.124819 | 0.135011 | 0.135955 | 0.087996 | -496.5237 | -496.5135 | -496.5126 | -496.5605 |
| TS19 | 0.126006 | 0.135092 | 0.136036 | 0.090919 | -496.5911 | -496.5821 | -496.5811 | -496.6262 |
| IM16 | 0.127164 | 0.136416 | 0.13736 | 0.091588 | -496.6092 | -496.5999 | -496.599 | -496.6447 |
| TS20.1 | 0.123661 | 0.133047 | 0.133991 | 0.088176 | -496.5224 | -496.513 | -496.5121 | -496.5579 |
| TS20.2 | 0.123187 | 0.13264 | 0.133585 | 0.087837 | -496.527 | -496.5176 | -496.5166 | -496.5624 |
| FS11.1 | 0.124994 | 0.135122 | 0.136066 | 0.08816 | -496.5278 | -496.5177 | -496.5167 | -496.5646 |
| FS11.2 | 0.124336 | 0.134552 | 0.135496 | 0.087721 | -496.5334 | -496.5232 | -496.5223 | -496.57 |
| TS21 | 0.126001 | 0.135191 | 0.136135 | 0.090587 | -496.5912 | -496.5821 | -496.5811 | -496.6267 |
| IM17 | 0.12869 | 0.137444 | 0.138388 | 0.094143 | -496.6148 | -496.6061 | -496.6052 | -496.6494 |
| TS22 | 0.123567 | 0.132912 | 0.133856 | 0.088203 | -496.5331 | -496.5238 | -496.5228 | -496.5685 |
| FS12 | 0.124554 | 0.134684 | 0.135629 | 0.087991 | -496.5334 | -496.5233 | -496.5224 | -496.57 |

# Table S3(b): Absolute Energy Data for All Species Calculated at the G4MP2 Level of Theory.

| Species | E(ZPE) | E(Thermal) | G4MP2(0 K) | G4MP2 Energy | G4MP2 Enthalpy | G4MP2 Free Energy |
| --- | --- | --- | --- | --- | --- | --- |
| IS | 0.125654 | 0.131859 | -271.1053 | -271.0991 | -271.0982 | -271.1354 |
| ·OH | 0.008274 | 0.010634 | -75.67521 | -75.67285 | -75.67191 | -75.69214 |
| H_2_O | 0.020803 | 0.023639 | -76.36734 | -76.3645 | -76.36356 | -76.385 |
| COM1 | 0.136339 | 0.145515 | -346.7834 | -346.7742 | -346.7733 | -346.8189 |
| TS1 | 0.130561 | 0.139229 | -346.7737 | -346.765 | -346.7641 | -346.808 |
| IM1 | 0.112818 | 0.11912 | -270.4268 | -270.4205 | -270.4196 | -270.4584 |
| FS1 | 0.129774 | 0.136959 | -346.2753 | -346.2681 | -346.2672 | -346.3062 |
| TS2 | 0.130781 | 0.13932 | -346.773 | -346.7644 | -346.7635 | -346.807 |
| IM2 | 0.11273 | 0.119034 | -270.4271 | -270.4207 | -270.4198 | -270.459 |
| FS2 | 0.129778 | 0.136994 | -346.2746 | -346.2674 | -346.2664 | -346.3057 |
| TS3 | 0.130417 | 0.139144 | -346.7729 | -346.7641 | -346.7632 | -346.8075 |
| IM3 | 0.112703 | 0.119024 | -270.4262 | -270.4198 | -270.4189 | -270.4584 |
| FS3 | 0.129579 | 0.136978 | -346.2739 | -346.2665 | -346.2655 | -346.3055 |
| TS4 | 0.136463 | 0.144538 | -346.7797 | -346.7716 | -346.7707 | -346.8124 |
| IM4 | 0.13809 | 0.145958 | -346.8109 | -346.803 | -346.802 | -346.8432 |
| TS5 | 0.134351 | 0.14251 | -346.7168 | -346.7087 | -346.7077 | -346.7493 |
| FS4 | 0.135201 | 0.144439 | -346.7261 | -346.7168 | -346.7159 | -346.7607 |
| TS6 | 0.136475 | 0.144718 | -346.78 | -346.7718 | -346.7708 | -346.8134 |
| IM5 | 0.138636 | 0.146565 | -346.8092 | -346.8013 | -346.8003 | -346.842 |
| TS7.1 | 0.135062 | 0.143479 | -346.7219 | -346.7135 | -346.7125 | -346.7554 |
| TS7.2 | 0.135071 | 0.143341 | -346.7166 | -346.7084 | -346.7074 | -346.7498 |
| FS5.1 | 0.135941 | 0.145397 | -346.7243 | -346.7148 | -346.7139 | -346.7599 |
| FS5.2 | 0.135466 | 0.144631 | -346.7192 | -346.71 | -346.7091 | -346.7536 |
| TS8 | 0.13624 | 0.144653 | -346.7783 | -346.7699 | -346.7689 | -346.8122 |
| IM6 | 0.138559 | 0.146482 | -346.8077 | -346.7998 | -346.7989 | -346.8404 |
| TS9.1 | 0.135329 | 0.143476 | -346.716 | -346.7079 | -346.7069 | -346.7489 |
| TS9.2 | 0.134918 | 0.143154 | -346.7174 | -346.7092 | -346.7082 | -346.7503 |
| FS6.1 | 0.135879 | 0.145112 | -346.7192 | -346.71 | -346.7091 | -346.7542 |
| FS6.2 | 0.135188 | 0.144478 | -346.7209 | -346.7116 | -346.7107 | -346.7555 |
| TS10 | 0.136079 | 0.14461 | -346.7792 | -346.7707 | -346.7698 | -346.8136 |
| IM7 | 0.138687 | 0.146733 | -346.8081 | -346.8 | -346.7991 | -346.8413 |
| TS11 | 0.134599 | 0.14291 | -346.7172 | -346.7089 | -346.7079 | -346.7502 |
| FS7 | 0.135092 | 0.144394 | -346.7201 | -346.7108 | -346.7099 | -346.7549 |
| TS12 | 0.135042 | 0.14345 | -346.7827 | -346.7743 | -346.7734 | -346.8169 |
| IM8 | 0.112659 | 0.118413 | -270.4615 | -270.4557 | -270.4548 | -270.4912 |
| IM9 | 0.130716 | 0.137992 | -346.2635 | -346.2562 | -346.2553 | -346.2958 |
| COM2.1 | 0.142311 | 0.151822 | -421.948 | -421.9385 | -421.9375 | -421.9842 |
| TS13.1 | 0.140993 | 0.150038 | -421.9423 | -421.9332 | -421.9323 | -421.9779 |
| IM10.1 | 0.117786 | 0.124737 | -345.6313 | -345.6244 | -345.6234 | -345.6629 |
| IM11.1 | 0.136448 | 0.144454 | -421.4388 | -421.4308 | -421.4298 | -421.4717 |
| TS14.1 | 0.129656 | 0.137463 | -421.3774 | -421.3696 | -421.3687 | -421.4103 |
| COM2.2 | 0.142872 | 0.152225 | -421.9423 | -421.933 | -421.932 | -421.9772 |
| TS13.2 | 0.137665 | 0.14675 | -421.9307 | -421.9216 | -421.9207 | -421.9665 |
| IM10.2 | 0.115945 | 0.122905 | -345.5989 | -345.5919 | -345.591 | -345.6311 |
| IM11.2 | 0.134181 | 0.14262 | -421.334 | -421.3256 | -421.3246 | -421.3682 |
| TS14.2 | 0.126584 | 0.134914 | -421.2625 | -421.2542 | -421.2533 | -421.296 |
| IM12 | 0.108227 | 0.114591 | -345.0726 | -345.0662 | -345.0653 | -345.1032 |
| IM13 | 0.095935 | 0.102324 | -344.4273 | -344.4209 | -344.4199 | -344.4585 |
| FS8 | 0.11341 | 0.120619 | -420.2692 | -420.262 | -420.2611 | -420.3013 |
| COM3 | 0.123286 | 0.133883 | -495.9465 | -495.9359 | -495.935 | -495.9848 |
| TS15 | 0.124082 | 0.132892 | -495.937 | -495.9282 | -495.9272 | -495.9715 |
| IM14 | 0.125719 | 0.134692 | -495.9669 | -495.9579 | -495.957 | -496.0015 |
| TS16 | 0.122208 | 0.13135 | -495.8781 | -495.869 | -495.868 | -495.9125 |
| FS9 | 0.122153 | 0.132329 | -495.8804 | -495.8702 | -495.8692 | -495.9167 |
| TS17 | 0.124073 | 0.133463 | -495.9389 | -495.9295 | -495.9285 | -495.9745 |
| IM15 | 0.127323 | 0.13579 | -495.9741 | -495.9656 | -495.9647 | -496.0081 |
| TS18.1 | 0.122993 | 0.132308 | -495.8754 | -495.866 | -495.8651 | -495.9106 |
| TS18.2 | 0.122972 | 0.132617 | -495.8814 | -495.8718 | -495.8709 | -495.9187 |
| FS10.1 | 0.124141 | 0.134414 | -495.8824 | -495.8721 | -495.8712 | -495.9200 |
| FS10.2 | 0.123578 | 0.133841 | -495.8756 | -495.8653 | -495.8644 | -495.9125 |
| TS19 | 0.124228 | 0.133468 | -495.9378 | -495.9286 | -495.9277 | -495.9732 |
| IM16 | 0.125542 | 0.134877 | -495.9672 | -495.9578 | -495.9569 | -496.0029 |
| TS20.1 | 0.122229 | 0.131708 | -495.873 | -495.8635 | -495.8626 | -495.9086 |
| TS20.2 | 0.122102 | 0.13149 | -495.8763 | -495.8669 | -495.866 | -495.9114 |
| FS11.1 | 0.123564 | 0.133928 | -495.8749 | -495.8645 | -495.8636 | -495.9123 |
| FS11.2 | 0.123183 | 0.133395 | -495.8849 | -495.8747 | -495.8738 | -495.9214 |
| TS21 | 0.124257 | 0.13359 | -495.9378 | -495.9285 | -495.9275 | -495.9734 |
| IM17 | 0.126862 | 0.135757 | -495.9716 | -495.9627 | -495.9618 | -496.0063 |
| TS22 | 0.122466 | 0.131809 | -495.8791 | -495.8698 | -495.8688 | -495.9144 |
| FS12 | 0.123024 | 0.133309 | -495.8812 | -495.8709 | -495.87 | -495.9183 |

# Table S4: Cartesian Coordinates of Each Species Calculated Using the B3LYP/6-311++G(d,p) Computational Scheme.

| Species | Cartesian | | | |
| --- | --- | --- | --- | --- |
| IS | C | 1.196001 | -1.206008 | 0.001212 |
|  | C | 1.903241 | -0.002729 | 0.004596 |
|  | C | 1.203241 | 1.202169 | 0.001294 |
|  | C | -0.192285 | 1.202887 | -0.004783 |
|  | C | -0.913747 | 0.004116 | -0.00664 |
|  | C | -0.19711 | -1.200333 | -0.004784 |
|  | H | 1.731017 | -2.149443 | 0.000868 |
|  | H | 2.987419 | -0.00593 | 0.007261 |
|  | H | 1.741934 | 2.143489 | 0.000943 |
|  | H | -0.729404 | 2.145899 | -0.010154 |
|  | H | -0.738429 | -2.141239 | -0.009697 |
|  | C | -2.422299 | 0.002266 | 0.004609 |
|  | H | -2.825571 | -0.694242 | -0.735815 |
|  | H | -2.808179 | -0.30906 | 0.981285 |
|  | H | -2.821038 | 0.996317 | -0.207726 |
| COM1 | C | -2.068266 | -0.000152 | -0.004573 |
|  | C | -1.377106 | -1.208923 | -0.120406 |
|  | C | -0.006906 | -1.208894 | -0.348805 |
|  | C | 0.711221 | 0.001538 | -0.463544 |
|  | C | -0.007335 | 1.211103 | -0.342452 |
|  | C | -1.377498 | 1.209479 | -0.114009 |
|  | H | -3.137698 | -0.000795 | 0.172462 |
|  | H | -1.909734 | -2.148868 | -0.032285 |
|  | H | 0.527974 | -2.147651 | -0.442119 |
|  | H | 0.527281 | 2.150493 | -0.430831 |
|  | H | -1.91042 | 2.148776 | -0.020879 |
|  | C | 2.159708 | 0.002972 | -0.873401 |
|  | O | 1.228001 | -0.006109 | 1.900742 |
|  | H | 0.282458 | -0.005965 | 2.123542 |
|  | H | 2.675728 | 0.889505 | -0.501301 |
|  | H | 2.676324 | -0.884966 | -0.505526 |
|  | H | 2.241173 | 0.005604 | -1.965858 |
| COM2.1 | C | 2.531475 | -0.567491 | -0.215106 |
|  | C | 2.571235 | 0.824682 | -0.176646 |
|  | C | 1.40116 | 1.54421 | 0.072484 |
|  | C | 0.200563 | 0.87322 | 0.289391 |
|  | C | 0.151272 | -0.526266 | 0.25352 |
|  | C | 1.325713 | -1.237585 | -0.005711 |
|  | H | 3.436059 | -1.132415 | -0.410328 |
|  | H | 3.506985 | 1.347495 | -0.339455 |
|  | H | 1.426332 | 2.627769 | 0.103091 |
|  | H | -0.707889 | 1.433696 | 0.479699 |
|  | H | 1.296783 | -2.32162 | -0.03905 |
|  | C | -1.146311 | -1.251775 | 0.494845 |
|  | H | -0.971852 | -2.329527 | 0.526531 |
|  | H | -1.586776 | -0.942614 | 1.448676 |
|  | O | -2.073354 | -0.948603 | -0.57956 |
|  | H | -2.840459 | -1.525451 | -0.465271 |
|  | O | -3.293477 | 1.481568 | -0.080811 |
|  | H | -2.83517 | 0.62498 | -0.297588 |
| COM2.2 | C | -1.540413 | 1.321953 | -0.25716 |
|  | C | -2.424564 | 0.26897 | -0.00558 |
|  | C | -1.952849 | -1.046375 | 0.043887 |
|  | C | -0.60572 | -1.309595 | -0.156141 |
|  | C | 0.302353 | -0.259803 | -0.410336 |
|  | C | -0.193281 | 1.063286 | -0.466823 |
|  | H | -1.907378 | 2.34088 | -0.296379 |
|  | H | -3.477151 | 0.472458 | 0.154073 |
|  | H | -2.639437 | -1.860922 | 0.24287 |
|  | H | -0.235286 | -2.327921 | -0.114294 |
|  | H | 0.497041 | 1.872841 | -0.670137 |
|  | C | 1.737711 | -0.55921 | -0.785726 |
|  | H | 1.999312 | -1.563091 | -0.43173 |
|  | H | 1.83787 | -0.558796 | -1.875633 |
|  | O | 2.648388 | 0.417734 | -0.289678 |
|  | H | 2.399303 | 0.50884 | 0.649123 |
|  | O | 0.997167 | 0.014086 | 1.86164 |
|  | H | 0.421869 | 0.785789 | 1.99369 |
| COM3 | C | 2.098171 | 0.844234 | -0.453757 |
|  | C | 1.622846 | -0.391425 | -0.909181 |
|  | C | 0.275035 | -0.726345 | -0.743632 |
|  | C | -0.596734 | 0.16934 | -0.121971 |
|  | C | -0.112416 | 1.411966 | 0.318161 |
|  | C | 1.227899 | 1.742238 | 0.161304 |
|  | H | 3.142183 | 1.100834 | -0.587825 |
|  | H | 2.280309 | -1.067179 | -1.440542 |
|  | H | -0.073142 | -1.676543 | -1.131025 |
|  | H | -0.797051 | 2.106854 | 0.787504 |
|  | H | 1.592672 | 2.699333 | 0.513735 |
|  | C | -2.050263 | -0.110868 | 0.062116 |
|  | O | -2.471976 | -1.392372 | -0.023058 |
|  | H | -1.734215 | -2.015706 | -0.084691 |
|  | O | -2.872079 | 0.755277 | 0.281316 |
|  | O | 2.519104 | -1.536678 | 1.15378 |
|  | H | 3.40163 | -1.392246 | 0.768307 |
| IM1 | C | -1.893985 | 0.002182 | 0.000071 |
|  | C | -1.226061 | -1.233374 | 0.000135 |
|  | C | 0.145758 | -1.190616 | 0.000001 |
|  | C | 0.946535 | -0.064833 | -0.000146 |
|  | C | 0.242602 | 1.153126 | -0.000161 |
|  | C | -1.153651 | 1.183054 | -0.000084 |
|  | H | -2.97821 | 0.029627 | 0.000094 |
|  | H | -1.779511 | -2.166355 | 0.000241 |
|  | H | 0.80498 | 2.08215 | -0.000384 |
|  | H | -1.666017 | 2.138433 | -0.000228 |
|  | C | 2.453387 | -0.124526 | 0.000106 |
|  | H | 2.824699 | -0.651432 | 0.883574 |
|  | H | 2.824759 | -0.66152 | -0.877182 |
|  | H | 2.881789 | 0.879016 | -0.005645 |
| IM2 | C | -1.953118 | -0.18444 | 0.00393 |
|  | C | -1.115644 | -1.275765 | 0.001461 |
|  | C | 0.256235 | -1.249013 | -0.004191 |
|  | C | 0.88178 | 0.015562 | -0.006095 |
|  | C | 0.070953 | 1.156772 | -0.004256 |
|  | C | -1.322085 | 1.067295 | 0.001207 |
|  | H | -3.033864 | -0.269774 | 0.005684 |
|  | H | 0.539649 | 2.134897 | -0.008622 |
|  | H | -1.923411 | 1.970504 | 0.001073 |
|  | C | 2.386159 | 0.118919 | 0.004018 |
|  | H | 2.792397 | -0.18047 | 0.975519 |
|  | H | 2.832683 | -0.53764 | -0.74741 |
|  | H | 2.712334 | 1.14141 | -0.194484 |
|  | H | 0.854529 | -2.154907 | -0.008199 |
| IM3 | C | -1.901638 | 0 | 0.009515 |
|  | C | -1.272191 | -1.221877 | 0.002985 |
|  | C | 0.130195 | -1.206486 | -0.008176 |
|  | C | 0.842279 | 0 | -0.011697 |
|  | C | 0.130195 | 1.206486 | -0.008176 |
|  | C | -1.272192 | 1.221877 | 0.002985 |
|  | H | 0.67029 | 2.148229 | -0.01708 |
|  | H | -1.815802 | 2.160359 | 0.002609 |
|  | C | 2.351947 | 0 | 0.007565 |
|  | H | 2.729429 | -0.000005 | 1.035811 |
|  | H | 2.75501 | -0.885605 | -0.488437 |
|  | H | 2.75501 | 0.885609 | -0.488429 |
|  | H | 0.670291 | -2.148228 | -0.01708 |
|  | H | -1.815802 | -2.160359 | 0.002609 |
| IM4 | C | 1.400631 | 1.225153 | -0.030961 |
|  | C | 2.111697 | -0.000003 | -0.072768 |
|  | C | 1.400628 | -1.225156 | -0.030976 |
|  | C | 0.037546 | -1.249423 | 0.020495 |
|  | C | -0.802331 | 0.000005 | 0.025571 |
|  | C | 0.037551 | 1.249432 | 0.020524 |
|  | H | 1.955284 | 2.157864 | -0.032643 |
|  | H | 3.193983 | -0.000004 | -0.112965 |
|  | H | 1.955271 | -2.157873 | -0.032664 |
|  | H | -0.504108 | -2.188876 | 0.063134 |
|  | H | -0.504094 | 2.188891 | 0.063162 |
|  | C | -1.789563 | 0.000014 | -1.151897 |
|  | H | -2.424363 | 0.888845 | -1.104893 |
|  | H | -1.254949 | 0.000214 | -2.103395 |
|  | H | -2.42412 | -0.888997 | -1.105141 |
|  | O | -1.661862 | -0.000014 | 1.213095 |
|  | H | -1.074961 | -0.000076 | 1.980711 |
| IM5 | C | -1.092172 | 1.165911 | 0.332833 |
|  | C | -2.031828 | 0.220195 | 0.047048 |
|  | C | -1.657088 | -1.123448 | -0.202795 |
|  | C | -0.293066 | -1.494868 | -0.165125 |
|  | C | 0.702905 | -0.594704 | 0.110565 |
|  | C | 0.372843 | 0.855714 | 0.375582 |
|  | H | -1.372363 | 2.196966 | 0.520267 |
|  | H | -3.081034 | 0.494424 | 0.009055 |
|  | H | -2.413232 | -1.864858 | -0.430268 |
|  | H | -0.026595 | -2.529352 | -0.361162 |
|  | H | 0.782618 | 1.136835 | 1.356178 |
|  | C | 2.148872 | -0.980283 | 0.168935 |
|  | H | 2.736999 | -0.428849 | -0.572086 |
|  | H | 2.576936 | -0.732116 | 1.148399 |
|  | H | 2.286023 | -2.048684 | -0.008458 |
|  | O | 1.105336 | 1.740144 | -0.53071 |
|  | H | 0.765163 | 1.563379 | -1.41849 |
| IM6 | C | -1.264044 | -0.459941 | -0.422287 |
|  | C | -1.36693 | 1.017969 | -0.206014 |
|  | C | -0.270286 | 1.783659 | 0.069038 |
|  | C | 1.021224 | 1.213848 | 0.151909 |
|  | C | 1.215044 | -0.187469 | -0.037809 |
|  | C | 0.13757 | -0.979475 | -0.315414 |
|  | H | -1.674695 | -0.715574 | -1.409179 |
|  | H | -2.358381 | 1.454167 | -0.261325 |
|  | H | -0.385602 | 2.850118 | 0.23418 |
|  | H | 1.873748 | 1.844644 | 0.376885 |
|  | H | 0.264037 | -2.048489 | -0.45688 |
|  | C | 2.606513 | -0.758954 | 0.082882 |
|  | H | 2.61017 | -1.835876 | -0.094651 |
|  | H | 3.285826 | -0.291425 | -0.636688 |
|  | H | 3.020004 | -0.575301 | 1.079455 |
|  | O | -2.158244 | -1.182798 | 0.482111 |
|  | H | -1.843697 | -0.997711 | 1.377492 |
| IM7 | C | -0.703266 | -1.247106 | -0.276553 |
|  | C | -1.52518 | -0.000112 | -0.39149 |
|  | C | -0.703741 | 1.247134 | -0.276518 |
|  | C | 0.643358 | 1.22026 | -0.069028 |
|  | C | 1.369982 | 0.000536 | 0.03399 |
|  | C | 0.64357 | -1.219781 | -0.069141 |
|  | H | -1.236815 | -2.189312 | -0.343265 |
|  | H | -2.072744 | -0.000242 | -1.343784 |
|  | H | -1.237538 | 2.189189 | -0.343196 |
|  | H | 1.187704 | 2.155378 | 0.025901 |
|  | H | 1.188289 | -2.154698 | 0.025778 |
|  | C | 2.858041 | -0.000053 | 0.2208 |
|  | H | 3.191013 | -0.879129 | 0.778586 |
|  | H | 3.378802 | -0.019732 | -0.746124 |
|  | H | 3.195717 | 0.896296 | 0.747019 |
|  | O | -2.612444 | -0.000382 | 0.588492 |
|  | H | -2.191465 | 0.000042 | 1.458784 |
| IM8 | C | -1.131974 | -1.211099 | 0.000003 |
|  | C | -1.837115 | 0.000002 | -0.000021 |
|  | C | -1.131961 | 1.211106 | -0.000003 |
|  | C | 0.252086 | 1.218556 | 0.000012 |
|  | C | 0.994048 | -0.000005 | 0.00001 |
|  | C | 0.252082 | -1.218559 | 0.000022 |
|  | H | -1.674975 | -2.149866 | -0.000004 |
|  | H | -2.920797 | 0.000017 | -0.000048 |
|  | H | -1.674966 | 2.149869 | -0.000007 |
|  | H | 0.793164 | 2.158799 | 0.000026 |
|  | H | 0.793142 | -2.158811 | 0.000057 |
|  | C | 2.39844 | -0.000002 | -0.000021 |
|  | H | 2.955398 | 0.929341 | -0.000016 |
|  | H | 2.9554 | -0.929344 | -0.000018 |
| IM9 | C | -1.427703 | -1.319769 | 0.046774 |
|  | C | -2.309705 | -0.249405 | -0.112315 |
|  | C | -1.81508 | 1.053018 | -0.134833 |
|  | C | -0.445275 | 1.283423 | -0.002176 |
|  | C | 0.444156 | 0.218844 | 0.165096 |
|  | C | -0.061873 | -1.086694 | 0.186844 |
|  | H | -1.805169 | -2.336186 | 0.064707 |
|  | H | -3.373076 | -0.431153 | -0.220571 |
|  | H | -2.492377 | 1.889925 | -0.263829 |
|  | H | -0.063571 | 2.298784 | -0.031471 |
|  | H | 0.619597 | -1.921064 | 0.308453 |
|  | C | 1.919859 | 0.474132 | 0.346135 |
|  | H | 2.141853 | 1.523144 | 0.121301 |
|  | H | 2.201231 | 0.289874 | 1.390261 |
|  | O | 2.670106 | -0.396866 | -0.516475 |
|  | H | 3.584392 | -0.379693 | -0.210195 |
| IM10.1 | C | -1.346078 | -1.344344 | 0.000008 |
|  | C | -2.279778 | -0.300077 | 0.000014 |
|  | C | -1.823522 | 1.026493 | 0.000023 |
|  | C | -0.46887 | 1.307948 | 0.000013 |
|  | C | 0.495243 | 0.258269 | -0.000008 |
|  | C | 0.014778 | -1.083831 | -0.000005 |
|  | H | -1.690847 | -2.372962 | 0.000001 |
|  | H | -3.342044 | -0.513918 | 0.000035 |
|  | H | -2.538104 | 1.842582 | 0.000033 |
|  | H | -0.126432 | 2.337403 | 0.000015 |
|  | H | 0.724819 | -1.901565 | -0.000017 |
|  | C | 1.865637 | 0.566962 | -0.000017 |
|  | H | 2.235905 | 1.584378 | -0.000031 |
|  | O | 2.789651 | -0.438803 | -0.000015 |
|  | H | 3.675037 | -0.054011 | -0.000081 |
| IM10.2 | C | -1.797266 | -1.036884 | -0.000078 |
|  | C | -2.267196 | 0.27705 | -0.000055 |
|  | C | -1.358216 | 1.334968 | 0.000039 |
|  | C | 0.013123 | 1.083124 | 0.000124 |
|  | C | 0.487931 | -0.229186 | 0.000063 |
|  | C | -0.427142 | -1.288013 | -0.00003 |
|  | H | -2.497322 | -1.864846 | -0.000143 |
|  | H | -3.333245 | 0.473898 | -0.000128 |
|  | H | -1.716781 | 2.358262 | 0.000024 |
|  | H | 0.719711 | 1.904382 | 0.000172 |
|  | H | -0.063997 | -2.311053 | -0.000054 |
|  | C | 1.96913 | -0.533067 | 0.000101 |
|  | H | 2.238253 | -1.188052 | -0.855554 |
|  | H | 2.23832 | -1.18584 | 0.8576 |
|  | O | 2.836609 | 0.520661 | -0.000363 |
| IM11.1 | C | -1.920414 | -1.206981 | -0.054591 |
|  | C | -2.627342 | -0.007234 | -0.108577 |
|  | C | -1.938067 | 1.202298 | -0.0233 |
|  | C | -0.551289 | 1.211693 | 0.111206 |
|  | C | 0.164884 | 0.010704 | 0.159295 |
|  | C | -0.532608 | -1.198132 | 0.081045 |
|  | H | -2.447437 | -2.152264 | -0.118943 |
|  | H | -3.706197 | -0.013794 | -0.214961 |
|  | H | -2.480135 | 2.140447 | -0.063388 |
|  | H | -0.022041 | 2.153827 | 0.174592 |
|  | H | 0.011242 | -2.133522 | 0.123296 |
|  | C | 1.673797 | 0.019076 | 0.346884 |
|  | H | 1.921403 | 0.07463 | 1.411466 |
|  | O | 2.296086 | -1.175196 | -0.090783 |
|  | H | 2.049606 | -1.313798 | -1.016583 |
|  | O | 2.197778 | 1.149323 | -0.326677 |
|  | H | 3.108881 | 1.262914 | -0.027566 |
| IM11.2 | C | 2.111143 | 1.200886 | -0.142736 |
|  | C | 2.778529 | -0.014638 | -0.292252 |
|  | C | 2.086973 | -1.213852 | -0.118621 |
|  | C | 0.732596 | -1.196181 | 0.206957 |
|  | C | 0.057348 | 0.018876 | 0.366 |
|  | C | 0.755721 | 1.216407 | 0.180785 |
|  | H | 2.645475 | 2.134658 | -0.276022 |
|  | H | 3.833648 | -0.027515 | -0.541002 |
|  | H | 2.603107 | -2.160265 | -0.233116 |
|  | H | 0.194918 | -2.128012 | 0.346398 |
|  | H | 0.236016 | 2.161251 | 0.298816 |
|  | C | -1.411486 | 0.033841 | 0.688363 |
|  | H | -1.703319 | 0.964617 | 1.182577 |
|  | H | -1.694515 | -0.818332 | 1.31128 |
|  | O | -2.101801 | -0.062412 | -0.57884 |
|  | O | -3.526037 | -0.093401 | -0.30462 |
|  | H | -3.757569 | 0.848064 | -0.352221 |
| IM12 | C | 1.331166 | -1.327494 | -0.000015 |
|  | C | 2.213553 | -0.24221 | -0.000016 |
|  | C | 1.726229 | 1.064599 | -0.000001 |
|  | C | 0.352711 | 1.289362 | 0.000009 |
|  | C | -0.534805 | 0.205843 | 0.000014 |
|  | C | -0.038749 | -1.10662 | 0.000007 |
|  | H | 1.717996 | -2.339771 | -0.000036 |
|  | H | 3.283192 | -0.418509 | -0.000034 |
|  | H | 2.414437 | 1.901575 | -0.000005 |
|  | H | -0.039057 | 2.301133 | 0.000006 |
|  | H | -0.735859 | -1.936297 | 0.000008 |
|  | C | -1.982839 | 0.467832 | -0.000035 |
|  | H | -2.262732 | 1.537172 | -0.000042 |
|  | O | -2.847697 | -0.394147 | 0.000041 |
| IM13 | C | 1.278736 | 1.330844 | 0.000009 |
|  | C | 2.172753 | 0.25563 | 0.000005 |
|  | C | 1.703526 | -1.05807 | 0.000008 |
|  | C | 0.334207 | -1.303005 | 0.000013 |
|  | C | -0.56269 | -0.227906 | 0.000009 |
|  | C | -0.089104 | 1.0956 | 0.000008 |
|  | H | 1.65357 | 2.34766 | 0.000012 |
|  | H | 3.240024 | 0.445235 | 0 |
|  | H | 2.402703 | -1.885596 | 0.000003 |
|  | H | -0.050682 | -2.315987 | 0.000018 |
|  | H | -0.7967 | 1.916222 | 0.000006 |
|  | C | -2.004539 | -0.505405 | -0.000003 |
|  | O | -2.930781 | 0.245792 | -0.000042 |
| IM14 | C | 2.485636 | 0.277493 | -0.160312 |
|  | C | 1.846968 | 0.445754 | 1.093788 |
|  | C | 0.542985 | 0.096246 | 1.280829 |
|  | C | -0.303663 | -0.476674 | 0.174686 |
|  | C | 0.464718 | -0.661983 | -1.113796 |
|  | C | 1.772044 | -0.296637 | -1.240195 |
|  | H | 3.523794 | 0.558576 | -0.282491 |
|  | H | 2.414848 | 0.843952 | 1.927606 |
|  | H | 0.058614 | 0.202265 | 2.244864 |
|  | H | -0.080792 | -1.119859 | -1.931213 |
|  | H | 2.28401 | -0.467064 | -2.181167 |
|  | C | -1.504669 | 0.466648 | -0.124606 |
|  | O | -1.24077 | 1.758164 | -0.339168 |
|  | H | -0.295911 | 1.949263 | -0.216365 |
|  | O | -2.638564 | 0.048816 | -0.191628 |
|  | O | -0.860925 | -1.72938 | 0.604925 |
|  | H | -1.806607 | -1.693011 | 0.38338 |
| IM15 | C | 2.5883 | -0.246818 | -0.019005 |
|  | C | 1.921622 | 0.913013 | -0.262795 |
|  | C | 0.431776 | 0.970305 | -0.379831 |
|  | C | -0.252719 | -0.341498 | -0.071377 |
|  | C | 0.481617 | -1.484599 | 0.148156 |
|  | C | 1.885538 | -1.466894 | 0.168602 |
|  | H | 3.670663 | -0.244871 | 0.043464 |
|  | H | 2.448234 | 1.850115 | -0.402535 |
|  | H | 0.173953 | 1.267843 | -1.406691 |
|  | H | -0.043563 | -2.414174 | 0.333424 |
|  | H | 2.431311 | -2.381763 | 0.36116 |
|  | C | -1.728683 | -0.41627 | -0.058104 |
|  | O | -2.395512 | 0.759214 | -0.022985 |
|  | H | -1.754719 | 1.488093 | 0.151336 |
|  | O | -2.361833 | -1.459575 | -0.081207 |
|  | O | -0.117423 | 2.071032 | 0.407963 |
|  | H | 0.107553 | 1.905956 | 1.335801 |
| IM16 | C | -2.16122 | 0.817446 | -0.259638 |
|  | C | -1.794355 | -0.630932 | -0.314261 |
|  | C | -0.316669 | -0.872503 | -0.220473 |
|  | C | 0.587206 | 0.146994 | -0.085486 |
|  | C | 0.150387 | 1.500173 | -0.027813 |
|  | C | -1.225386 | 1.801129 | -0.124393 |
|  | H | -3.217512 | 1.053018 | -0.320861 |
|  | H | -2.164252 | -1.061038 | -1.25812 |
|  | H | -0.014295 | -1.914691 | -0.217733 |
|  | H | 0.879794 | 2.289006 | 0.093729 |
|  | H | -1.537708 | 2.838785 | -0.084503 |
|  | C | 2.052236 | -0.102236 | 0.074383 |
|  | O | 2.551763 | -1.277797 | -0.364784 |
|  | H | 1.885819 | -1.790439 | -0.844734 |
|  | O | 2.810616 | 0.699724 | 0.579166 |
|  | O | -2.488811 | -1.31377 | 0.769119 |
|  | H | -2.573584 | -2.240328 | 0.510296 |
| IM17 | C | 2.189398 | 0.038386 | -0.35676 |
|  | C | 1.356157 | 1.265227 | -0.147931 |
|  | C | 0.003748 | 1.221146 | -0.023867 |
|  | C | -0.700701 | -0.019632 | -0.061219 |
|  | C | 0.042029 | -1.234789 | -0.171911 |
|  | C | 1.392009 | -1.228206 | -0.304766 |
|  | H | 2.669491 | 0.109183 | -1.34449 |
|  | H | 1.886478 | 2.209137 | -0.094198 |
|  | H | -0.53028 | 2.151268 | 0.137045 |
|  | H | -0.501212 | -2.17091 | -0.14329 |
|  | H | 1.948013 | -2.155802 | -0.381897 |
|  | C | -2.163538 | -0.122702 | 0.077771 |
|  | O | -2.909466 | 1.00893 | -0.003314 |
|  | H | -2.382548 | 1.7766 | -0.265329 |
|  | O | -2.747304 | -1.176657 | 0.270358 |
|  | O | 3.312846 | -0.002201 | 0.555113 |
|  | H | 2.946831 | -0.076631 | 1.446997 |
| TS1 | C | 1.853616 | -1.169115 | 0.010585 |
|  | C | 0.502687 | -1.519157 | -0.039385 |
|  | C | -0.436917 | -0.500716 | -0.06625 |
|  | C | -0.129389 | 0.853557 | -0.024661 |
|  | C | 1.238627 | 1.173758 | 0.024351 |
|  | C | 2.212766 | 0.17917 | 0.041863 |
|  | H | 2.612591 | -1.943029 | 0.02552 |
|  | H | 0.197998 | -2.558919 | -0.061924 |
|  | H | -1.61399 | -0.821487 | -0.16275 |
|  | H | 1.531752 | 2.218663 | 0.051419 |
|  | H | 3.259958 | 0.45713 | 0.083503 |
|  | C | -1.187041 | 1.927524 | -0.02831 |
|  | H | -1.025412 | 2.633646 | -0.847421 |
|  | H | -2.180863 | 1.493117 | -0.13602 |
|  | H | -1.163215 | 2.500426 | 0.903502 |
|  | O | -2.871732 | -1.101065 | -0.032956 |
|  | H | -2.971049 | -0.841151 | 0.898664 |
| TS2 | C | 0.86867 | 1.380926 | -0.0438 |
|  | C | 1.045574 | 0.007723 | -0.090297 |
|  | C | 0.003921 | -0.900729 | -0.055639 |
|  | C | -1.315552 | -0.420762 | 0.007443 |
|  | C | -1.515451 | 0.963711 | 0.046969 |
|  | C | -0.443581 | 1.856176 | 0.021068 |
|  | H | 1.71202 | 2.060635 | -0.058267 |
|  | H | 2.182509 | -0.428273 | -0.190577 |
|  | H | -2.527918 | 1.349057 | 0.104561 |
|  | H | -0.626413 | 2.92466 | 0.05542 |
|  | C | -2.476756 | -1.38243 | 0.014952 |
|  | H | -2.640721 | -1.803876 | -0.982275 |
|  | H | -2.293931 | -2.219974 | 0.692829 |
|  | H | -3.398366 | -0.884436 | 0.321844 |
|  | O | 3.376181 | -0.893743 | -0.026699 |
|  | H | 3.386239 | -0.907638 | 0.945206 |
|  | H | 0.196178 | -1.967906 | -0.079325 |
| TS3 | C | -1.198604 | -0.000434 | -0.094049 |
|  | C | -0.535027 | -1.215997 | -0.056101 |
|  | C | 0.860288 | -1.203184 | -0.005476 |
|  | C | 1.573003 | 0.003442 | 0.01911 |
|  | C | 0.858011 | 1.206051 | -0.005416 |
|  | C | -0.53969 | 1.215199 | -0.056159 |
|  | H | -2.423047 | -0.002211 | -0.18487 |
|  | H | 1.394688 | 2.148822 | 0.01918 |
|  | H | -1.085907 | 2.151058 | -0.068228 |
|  | C | 3.081015 | 0.000056 | 0.05751 |
|  | H | 3.458427 | -0.71471 | 0.793299 |
|  | H | 3.495615 | -0.288571 | -0.914004 |
|  | H | 3.473103 | 0.987981 | 0.305509 |
|  | H | 1.39949 | -2.144824 | 0.0186 |
|  | H | -1.079418 | -2.152917 | -0.068345 |
|  | O | -3.69126 | -0.00172 | -0.003301 |
|  | H | -3.696845 | -0.001663 | 0.968756 |
| TS4 | C | -2.086621 | -0.000008 | -0.005034 |
|  | C | -1.392638 | -1.214131 | -0.09697 |
|  | C | -0.021009 | -1.219246 | -0.272797 |
|  | C | 0.720882 | -0.000005 | -0.323358 |
|  | C | -0.021013 | 1.219231 | -0.272842 |
|  | C | -1.392641 | 1.214114 | -0.097003 |
|  | H | -3.161813 | -0.000006 | 0.131198 |
|  | H | -1.934262 | -2.151173 | -0.039281 |
|  | H | 0.518048 | -2.156033 | -0.357422 |
|  | H | 0.51804 | 2.156016 | -0.357512 |
|  | H | -1.934269 | 2.151155 | -0.039347 |
|  | C | 2.125631 | -0.000004 | -0.865699 |
|  | O | 1.315823 | -0.000038 | 1.656442 |
|  | H | 0.432198 | 0.000641 | 2.05468 |
|  | H | 2.669627 | 0.887921 | -0.540767 |
|  | H | 2.669695 | -0.887828 | -0.54062 |
|  | H | 2.100611 | -0.000099 | -1.960254 |
| TS5 | C | 1.67099 | 1.064071 | -0.203305 |
|  | C | 2.00501 | -0.313358 | 0.119751 |
|  | C | 1.142338 | -1.358517 | -0.06316 |
|  | C | -0.226388 | -1.176799 | -0.444345 |
|  | C | -1.053585 | -0.197392 | 0.073227 |
|  | C | 0.442437 | 1.503888 | -0.471588 |
|  | H | 2.517841 | 1.749669 | -0.296946 |
|  | H | 3.038184 | -0.525271 | 0.37325 |
|  | H | 1.546873 | -2.367629 | -0.056773 |
|  | H | -0.631834 | -1.784456 | -1.24873 |
|  | H | 0.048451 | 2.481269 | -0.723581 |
|  | C | -2.407899 | 0.105045 | -0.486916 |
|  | H | -2.599347 | 1.180027 | -0.458307 |
|  | H | -2.493013 | -0.24178 | -1.516156 |
|  | H | -3.178732 | -0.381061 | 0.120235 |
|  | O | -0.946698 | 0.271278 | 1.365054 |
|  | H | -0.112251 | -0.04261 | 1.744589 |
| TS6 | C | 2.005789 | 0.040491 | -0.120718 |
|  | C | 1.504549 | -1.16591 | 0.386133 |
|  | C | 0.133941 | -1.444816 | 0.340293 |
|  | C | -0.764575 | -0.534507 | -0.20406 |
|  | C | -0.262543 | 0.719246 | -0.667014 |
|  | C | 1.134204 | 0.965381 | -0.66689 |
|  | H | 3.070871 | 0.238618 | -0.096933 |
|  | H | 2.18463 | -1.895434 | 0.81119 |
|  | H | -0.232646 | -2.391579 | 0.721596 |
|  | H | -0.903911 | 1.340725 | -1.278075 |
|  | H | 1.502478 | 1.898515 | -1.075805 |
|  | C | -2.236745 | -0.815562 | -0.277674 |
|  | O | -0.816867 | 1.928378 | 0.935039 |
|  | H | -0.31029 | 1.424911 | 1.590936 |
|  | H | -2.602298 | -0.728588 | -1.305733 |
|  | H | -2.471932 | -1.815158 | 0.090816 |
|  | H | -2.78968 | -0.084974 | 0.321275 |
| TS7.1 | C | -1.434327 | 1.450414 | 0.404779 |
|  | C | -2.19696 | 0.370806 | 0.281923 |
|  | C | -1.779731 | -0.91827 | -0.255162 |
|  | C | -0.49548 | -1.353072 | -0.37978 |
|  | C | 0.702408 | -0.584855 | -0.131283 |
|  | C | 0.759076 | 0.732418 | -0.500016 |
|  | H | -1.616494 | 2.444564 | 0.795635 |
|  | H | -3.221216 | 0.398646 | 0.668649 |
|  | H | -2.574013 | -1.634554 | -0.44057 |
|  | H | -0.347825 | -2.412079 | -0.583112 |
|  | C | 1.8506 | -1.264738 | 0.575134 |
|  | H | 1.733315 | -1.235515 | 1.664732 |
|  | H | 1.890396 | -2.318804 | 0.287958 |
|  | H | 2.818499 | -0.815831 | 0.333243 |
|  | O | 1.80141 | 1.577786 | -0.237624 |
|  | H | 2.356179 | 1.21261 | 0.46522 |
|  | H | 0.116359 | 1.14246 | -1.264323 |
| TS7.2 | C | -1.642609 | 0.082784 | -0.224428 |
|  | C | -1.499185 | -1.331419 | -0.037256 |
|  | C | -0.316264 | -1.995102 | 0.119685 |
|  | C | 1.008732 | -1.411163 | -0.014193 |
|  | C | 1.30859 | -0.115632 | 0.024148 |
|  | C | -0.827685 | 1.013993 | 0.357937 |
|  | H | -2.415461 | 0.436325 | -0.905548 |
|  | H | -2.39933 | -1.932269 | -0.140709 |
|  | H | -0.353075 | -3.076661 | 0.204976 |
|  | H | 1.810993 | -2.12282 | -0.242735 |
|  | C | 2.510835 | 0.718053 | -0.117405 |
|  | H | 2.380902 | 1.483746 | -0.889715 |
|  | H | 2.744919 | 1.245288 | 0.814915 |
|  | H | 3.387695 | 0.110528 | -0.387798 |
|  | O | -0.84579 | 2.346308 | 0.047974 |
|  | H | -1.328733 | 2.473337 | -0.781415 |
|  | H | -0.316072 | 0.842981 | 1.293312 |
| TS8 | C | -1.388168 | 0.957886 | -0.406025 |
|  | C | -0.3765 | 1.731961 | 0.136511 |
|  | C | 0.902842 | 1.19431 | 0.312405 |
|  | C | 1.20099 | -0.134274 | -0.057475 |
|  | C | 0.18573 | -0.908707 | -0.595777 |
|  | C | -1.136557 | -0.402482 | -0.729164 |
|  | H | -2.379794 | 1.365522 | -0.559116 |
|  | H | -0.567986 | 2.762147 | 0.413924 |
|  | H | 1.687302 | 1.813376 | 0.735063 |
|  | H | 0.386064 | -1.930199 | -0.898841 |
|  | H | -1.836354 | -0.931687 | -1.361285 |
|  | C | 2.594883 | -0.677332 | 0.126921 |
|  | O | -2.069764 | -1.291249 | 0.859209 |
|  | H | -1.510966 | -0.875351 | 1.533279 |
|  | H | 3.332741 | -0.049973 | -0.381593 |
|  | H | 2.868417 | -0.700004 | 1.186356 |
|  | H | 2.679368 | -1.692001 | -0.265834 |
| TS9.1 | C | -1.680849 | -0.245767 | -0.311028 |
|  | C | -1.364277 | 0.963088 | 0.242617 |
|  | C | -0.139611 | 1.661407 | -0.017784 |
|  | C | 1.084015 | 1.097574 | -0.22638 |
|  | C | 1.426209 | -0.314927 | -0.058935 |
|  | C | 0.518069 | -1.28932 | -0.043833 |
|  | H | -2.072914 | 1.407941 | 0.939274 |
|  | H | -0.17773 | 2.745888 | 0.053552 |
|  | H | 1.922874 | 1.769541 | -0.384394 |
|  | H | 0.596615 | -2.365397 | 0.060763 |
|  | C | 2.900434 | -0.606863 | 0.191867 |
|  | H | 3.07554 | -1.671008 | 0.356107 |
|  | H | 3.500056 | -0.288663 | -0.667448 |
|  | H | 3.262836 | -0.055821 | 1.065492 |
|  | O | -2.771862 | -0.98647 | 0.036605 |
|  | H | -3.133534 | -0.655031 | 0.871394 |
|  | H | -1.262792 | -0.586834 | -1.246726 |
| TS9.2 | C | 1.320392 | -0.551611 | 0.338498 |
|  | C | 1.02965 | 1.81457 | -0.124052 |
|  | C | -0.284506 | 2.001708 | -0.084999 |
|  | C | -1.301681 | 0.985368 | 0.146597 |
|  | C | -1.134721 | -0.364869 | 0.019929 |
|  | C | 0.149397 | -0.980837 | -0.215556 |
|  | H | 1.869137 | 2.479364 | -0.287875 |
|  | H | -0.678197 | 2.999241 | -0.307947 |
|  | H | -2.312816 | 1.354618 | 0.288759 |
|  | H | 0.187948 | -1.833804 | -0.892391 |
|  | C | -2.335689 | -1.280911 | -0.001134 |
|  | H | -2.357319 | -1.864741 | -0.928347 |
|  | H | -2.300108 | -2.002261 | 0.821951 |
|  | H | -3.269683 | -0.721028 | 0.07211 |
|  | O | 2.545467 | -1.072428 | 0.03527 |
|  | H | 2.478608 | -1.597796 | -0.775253 |
|  | H | 1.361641 | 0.025323 | 1.251123 |
| TS10 | C | -1.432737 | 0.000072 | -0.656763 |
|  | C | -0.715727 | 1.219069 | -0.526495 |
|  | C | 0.620171 | 1.211063 | -0.174289 |
|  | C | 1.314827 | -0.00002 | 0.01405 |
|  | C | 0.620118 | -1.211049 | -0.174383 |
|  | C | -0.715785 | -1.218966 | -0.526608 |
|  | H | -2.394022 | 0.000119 | -1.150992 |
|  | H | -1.235459 | 2.156782 | -0.682716 |
|  | H | 1.152142 | 2.148854 | -0.055196 |
|  | H | 1.152046 | -2.148872 | -0.055355 |
|  | H | -1.235557 | -2.156644 | -0.682908 |
|  | C | 2.75873 | -0.000051 | 0.428829 |
|  | O | -2.481069 | -0.00004 | 1.123919 |
|  | H | -1.687136 | -0.000513 | 1.679732 |
|  | H | 2.843015 | 0.000624 | 1.522125 |
|  | H | 3.277807 | -0.889155 | 0.064498 |
|  | H | 3.278138 | 0.888406 | 0.06341 |
| TS11 | C | 0.900643 | -1.082147 | -0.250083 |
|  | C | 1.684672 | -0.144654 | 0.357113 |
|  | C | 0.20805 | 1.772474 | -0.110831 |
|  | C | -1.016825 | 1.266882 | -0.173692 |
|  | C | -1.405112 | -0.133537 | 0.0167 |
|  | C | -0.523554 | -1.171441 | -0.095397 |
|  | H | 1.388744 | -1.803857 | -0.903855 |
|  | H | 0.594748 | 2.776184 | -0.243303 |
|  | H | -1.842806 | 1.929263 | -0.457349 |
|  | H | -0.949905 | -2.169311 | -0.177078 |
|  | C | -2.879998 | -0.398722 | 0.184636 |
|  | H | -3.099995 | -1.467639 | 0.157672 |
|  | H | -3.245076 | -0.005802 | 1.140613 |
|  | H | -3.463016 | 0.096518 | -0.599782 |
|  | O | 3.019789 | 0.011559 | 0.117361 |
|  | H | 3.262374 | -0.477145 | -0.682346 |
|  | H | 1.389363 | 0.376186 | 1.255864 |
| TS12 | C | 1.559935 | -1.295385 | -0.070165 |
|  | C | 2.320238 | -0.244933 | 0.441229 |
|  | C | 1.785607 | 1.041442 | 0.468617 |
|  | C | 0.500086 | 1.273974 | -0.010387 |
|  | C | -0.275308 | 0.229015 | -0.528326 |
|  | C | 0.275604 | -1.059317 | -0.549249 |
|  | H | 1.970289 | -2.299544 | -0.099447 |
|  | H | 3.323006 | -0.42749 | 0.812695 |
|  | H | 2.37189 | 1.865943 | 0.861002 |
|  | H | 0.088834 | 2.278861 | 0.012102 |
|  | H | -0.311356 | -1.880681 | -0.949968 |
|  | C | -1.669467 | 0.472459 | -1.015036 |
|  | H | -1.832602 | 1.510917 | -1.316262 |
|  | H | -2.425098 | 0.274903 | -0.196627 |
|  | H | -1.951593 | -0.19515 | -1.833368 |
|  | O | -3.226183 | -0.376893 | 1.091769 |
|  | H | -2.404077 | -0.616149 | 1.555632 |
| TS13.1 | C | 2.439481 | -0.762078 | 0.190131 |
|  | C | 2.673253 | 0.577134 | -0.11871 |
|  | C | 1.596846 | 1.452224 | -0.273753 |
|  | C | 0.293492 | 0.987797 | -0.119779 |
|  | C | 0.049851 | -0.356224 | 0.186482 |
|  | C | 1.133196 | -1.226492 | 0.340983 |
|  | H | 3.272446 | -1.444884 | 0.314032 |
|  | H | 3.6887 | 0.939423 | -0.232764 |
|  | H | 1.774431 | 2.496227 | -0.50603 |
|  | H | -0.545806 | 1.667284 | -0.219684 |
|  | H | 0.952044 | -2.269313 | 0.578711 |
|  | C | -1.359911 | -0.860018 | 0.311049 |
|  | H | -1.397143 | -1.820728 | 0.83219 |
|  | H | -1.95705 | -0.136965 | 0.92085 |
|  | O | -1.948262 | -0.966775 | -0.990641 |
|  | H | -2.779828 | -1.449209 | -0.891842 |
|  | O | -3.162591 | 1.253589 | 0.54635 |
|  | H | -3.078208 | 0.849593 | -0.339552 |
| TS13.2 | C | 1.495178 | -1.404766 | -0.39911 |
|  | C | 2.585266 | -0.67889 | 0.075974 |
|  | C | 2.417271 | 0.658986 | 0.441139 |
|  | C | 1.168125 | 1.261187 | 0.330107 |
|  | C | 0.067579 | 0.534476 | -0.143384 |
|  | C | 0.241887 | -0.80164 | -0.510374 |
|  | H | 1.61747 | -2.443771 | -0.684336 |
|  | H | 3.558403 | -1.148728 | 0.162025 |
|  | H | 3.260252 | 1.230525 | 0.812944 |
|  | H | 1.04102 | 2.300379 | 0.61681 |
|  | H | -0.602803 | -1.368217 | -0.880428 |
|  | C | -1.278364 | 1.221281 | -0.241188 |
|  | H | -1.54728 | 1.672413 | 0.72013 |
|  | H | -1.197228 | 2.045727 | -0.965226 |
|  | O | -2.322699 | 0.409091 | -0.730327 |
|  | H | -2.786104 | -0.114867 | 0.062665 |
|  | O | -2.781112 | -1.038869 | 1.017868 |
|  | H | -2.694898 | -1.879042 | 0.536107 |
| TS14.1 | C | 2.093025 | -1.091839 | -0.083821 |
|  | C | 2.621781 | 0.17666 | 0.156399 |
|  | C | 1.778834 | 1.287988 | 0.205607 |
|  | C | 0.408811 | 1.132631 | 0.015843 |
|  | C | -0.125473 | -0.137691 | -0.220792 |
|  | C | 0.722235 | -1.248653 | -0.273417 |
|  | H | 2.748043 | -1.954354 | -0.127339 |
|  | H | 3.689113 | 0.300091 | 0.301001 |
|  | H | 2.191012 | 2.274192 | 0.386085 |
|  | H | -0.253968 | 1.988992 | 0.038355 |
|  | H | 0.305726 | -2.231984 | -0.465413 |
|  | C | -1.597574 | -0.313278 | -0.427675 |
|  | H | -2.793539 | 0.40772 | 0.456192 |
|  | H | -1.847899 | -1.257666 | -0.921277 |
|  | O | -2.357042 | 0.755241 | -0.73457 |
|  | O | -2.352733 | -0.523716 | 1.029118 |
|  | H | -1.77012 | -0.214098 | 1.743149 |
| TS14.2 | C | 1.757796 | -1.375633 | -0.025014 |
|  | C | 2.697181 | -0.370226 | 0.217991 |
|  | C | 2.295315 | 0.965155 | 0.248186 |
|  | C | 0.959028 | 1.294661 | 0.038883 |
|  | C | 0.017724 | 0.289355 | -0.216982 |
|  | C | 0.422467 | -1.050063 | -0.23741 |
|  | H | 2.069943 | -2.413391 | -0.049828 |
|  | H | 3.736887 | -0.627916 | 0.383658 |
|  | H | 3.020902 | 1.747955 | 0.436593 |
|  | H | 0.64116 | 2.331763 | 0.064817 |
|  | H | -0.309457 | -1.82578 | -0.427222 |
|  | C | -1.408078 | 0.681408 | -0.447333 |
|  | H | -1.575283 | 1.697232 | -0.84326 |
|  | H | -1.872499 | 0.826381 | 0.666339 |
|  | O | -2.285737 | -0.210627 | -0.851235 |
|  | O | -3.015806 | -0.247439 | 0.957791 |
|  | H | -3.747907 | -0.679651 | 1.44653 |
| TS15 | C | 2.610852 | 0.076282 | 0.206039 |
|  | C | 1.861549 | -0.839029 | 0.957994 |
|  | C | 0.483762 | -0.837759 | 0.881293 |
|  | C | -0.185931 | 0.069413 | -0.003289 |
|  | C | 0.595519 | 1.045057 | -0.697489 |
|  | C | 1.970408 | 1.01563 | -0.618083 |
|  | H | 3.692182 | 0.068195 | 0.272599 |
|  | H | 2.36322 | -1.542704 | 1.610972 |
|  | H | -0.109114 | -1.529819 | 1.466536 |
|  | H | 0.086649 | 1.785286 | -1.302229 |
|  | H | 2.559962 | 1.73204 | -1.177108 |
|  | C | -1.63884 | 0.386975 | 0.294792 |
|  | O | -2.506073 | -0.603866 | 0.094501 |
|  | H | -2.019119 | -1.254782 | -0.477423 |
|  | O | -1.97769 | 1.46445 | 0.735506 |
|  | O | -0.62052 | -1.220926 | -1.480544 |
|  | H | 0.076571 | -1.874909 | -1.316585 |
| TS16 | C | 2.297371 | 0.014089 | -0.216988 |
|  | C | 1.593142 | 1.106729 | -0.634052 |
|  | C | 0.312034 | 1.542204 | -0.150935 |
|  | C | -0.770991 | 0.7718 | 0.160558 |
|  | C | 0.692098 | -1.147352 | 1.277687 |
|  | C | 1.917863 | -0.904899 | 0.844632 |
|  | H | 3.300143 | -0.108484 | -0.611713 |
|  | H | 2.115939 | 1.805178 | -1.281348 |
|  | H | 0.203612 | 2.605794 | 0.048381 |
|  | H | 0.231221 | -1.761915 | 2.038979 |
|  | H | 2.753649 | -1.404083 | 1.345243 |
|  | C | -1.167955 | -0.537434 | -0.456738 |
|  | O | -0.443153 | -1.066632 | -1.450448 |
|  | H | 0.375794 | -0.563406 | -1.607227 |
|  | O | -2.230587 | -1.047272 | -0.14538 |
|  | O | -1.788166 | 1.322449 | 0.879632 |
|  | H | -2.526487 | 0.68772 | 0.852275 |
| TS17 | C | 2.51323 | -0.391295 | -0.221912 |
|  | C | 1.745978 | -1.507226 | 0.124472 |
|  | C | 0.348481 | -1.432363 | 0.140468 |
|  | C | -0.290035 | -0.244225 | -0.19295 |
|  | C | 0.482477 | 0.906782 | -0.514024 |
|  | C | 1.891194 | 0.800597 | -0.561809 |
|  | H | 3.593929 | -0.464286 | -0.239867 |
|  | H | 2.231927 | -2.441959 | 0.376205 |
|  | H | -0.21934 | -2.323689 | 0.382991 |
|  | H | -0.010962 | 1.750483 | -0.972904 |
|  | H | 2.473923 | 1.668471 | -0.84347 |
|  | C | -1.77135 | -0.097043 | -0.22838 |
|  | O | -2.513922 | -0.984813 | 0.467308 |
|  | H | -1.963011 | -1.560124 | 1.017424 |
|  | O | -2.337606 | 0.776995 | -0.851629 |
|  | O | 0.302046 | 1.942196 | 1.282109 |
|  | H | 0.769533 | 1.284726 | 1.822129 |
| TS18.1 | C | 2.240061 | 0.246582 | 0.299443 |
|  | C | 2.277479 | -1.157218 | 0.048069 |
|  | C | 1.219676 | -1.987373 | -0.218634 |
|  | C | -0.170631 | -1.624412 | -0.136404 |
|  | C | -0.65494 | -0.378158 | -0.053064 |
|  | C | 1.363457 | 1.126932 | -0.274518 |
|  | H | 2.973627 | 0.65442 | 0.99247 |
|  | H | 3.242303 | -1.636512 | 0.193153 |
|  | H | 1.431227 | -3.041539 | -0.357298 |
|  | H | -0.875427 | -2.459335 | -0.08455 |
|  | C | -1.979911 | 0.18564 | -0.019267 |
|  | O | 1.266432 | 2.429676 | 0.064144 |
|  | H | 1.752483 | 2.594489 | 0.886575 |
|  | H | 0.83301 | 0.920103 | -1.192829 |
|  | O | -2.255274 | 1.326927 | -0.36196 |
|  | O | -2.929309 | -0.668942 | 0.455111 |
|  | H | -3.783164 | -0.204872 | 0.430359 |
| TS18.2 | C | -2.653153 | -0.229363 | 0.480541 |
|  | C | -2.235734 | 1.02449 | 0.554178 |
|  | C | -0.133106 | 1.026291 | -0.693385 |
|  | C | 0.282418 | -0.230461 | -0.305902 |
|  | C | -0.618634 | -1.360966 | -0.445977 |
|  | C | -1.939859 | -1.340672 | -0.144072 |
|  | H | -3.586675 | -0.501101 | 0.98355 |
|  | H | -2.624038 | 1.946003 | 0.967332 |
|  | H | -0.98772 | 1.120296 | -1.348428 |
|  | H | -0.166412 | -2.308565 | -0.721234 |
|  | H | -2.500029 | -2.2623 | -0.258903 |
|  | C | 1.650508 | -0.518152 | 0.162144 |
|  | O | 2.258615 | 0.42513 | 0.955704 |
|  | H | 1.607236 | 0.894149 | 1.501012 |
|  | O | 2.281342 | -1.50927 | -0.137905 |
|  | O | 0.553225 | 2.184196 | -0.641811 |
|  | H | 1.397545 | 2.084072 | -0.176396 |
| TS19 | C | -2.160356 | 0.809214 | -0.398202 |
|  | C | -1.260849 | 1.737401 | 0.093923 |
|  | C | 0.088769 | 1.401971 | 0.242886 |
|  | C | 0.550566 | 0.12197 | -0.114502 |
|  | C | -0.352053 | -0.820489 | -0.596121 |
|  | C | -1.732168 | -0.513286 | -0.693347 |
|  | H | -3.20335 | 1.068169 | -0.531212 |
|  | H | -1.593945 | 2.733275 | 0.358727 |
|  | H | 0.763126 | 2.145389 | 0.652092 |
|  | H | -0.002512 | -1.804491 | -0.877562 |
|  | H | -2.370954 | -1.157814 | -1.280459 |
|  | C | 1.979636 | -0.294093 | 0.005101 |
|  | O | 2.915978 | 0.661014 | 0.192836 |
|  | H | 2.547543 | 1.554661 | 0.151712 |
|  | O | 2.340347 | -1.451162 | -0.061272 |
|  | O | -2.374741 | -1.488635 | 0.954502 |
|  | H | -1.873849 | -0.965054 | 1.599745 |
| TS20.1 | C | -2.222749 | 0.595863 | -0.714633 |
|  | C | -2.38428 | -0.655464 | -0.185706 |
|  | C | -0.070497 | -1.108011 | -0.383227 |
|  | C | 0.603349 | 0.005454 | -0.072333 |
|  | C | -0.033026 | 1.301181 | 0.14914 |
|  | C | -1.313644 | 1.585679 | -0.204424 |
|  | H | -2.769066 | 0.810573 | -1.628916 |
|  | H | -2.940987 | -1.41355 | -0.727408 |
|  | H | 0.196607 | -2.143463 | -0.55211 |
|  | H | 0.61488 | 2.101126 | 0.485002 |
|  | H | -1.61456 | 2.629531 | -0.236545 |
|  | C | 2.101924 | -0.002857 | 0.057276 |
|  | O | 2.773019 | -1.060322 | -0.447622 |
|  | H | 2.169506 | -1.666834 | -0.903085 |
|  | O | 2.732074 | 0.878453 | 0.604607 |
|  | O | -2.189594 | -0.915673 | 1.142696 |
|  | H | -2.266822 | -1.868116 | 1.289053 |
| TS20.2 | C | -2.394424 | 0.813657 | -0.522316 |
|  | C | -1.505767 | -1.315105 | -0.190728 |
|  | C | -0.285143 | -0.916191 | -0.671045 |
|  | C | 0.486028 | 0.173743 | -0.100771 |
|  | C | -0.103361 | 1.374313 | 0.188411 |
|  | C | -1.474682 | 1.697962 | -0.141848 |
|  | H | -3.432191 | 0.899158 | -0.819184 |
|  | H | -2.124018 | -1.991232 | -0.768793 |
|  | H | 0.018653 | -1.334125 | -1.627555 |
|  | H | 0.517837 | 2.166273 | 0.589845 |
|  | H | -1.73445 | 2.758704 | -0.11372 |
|  | C | 1.960925 | 0.069211 | 0.03682 |
|  | O | 2.508147 | -1.143469 | -0.224967 |
|  | H | 1.816365 | -1.800771 | -0.402882 |
|  | O | 2.698564 | 0.979322 | 0.364883 |
|  | O | -1.936935 | -1.255116 | 1.099783 |
|  | H | -1.321863 | -0.72945 | 1.633564 |
| TS21 | C | 2.130925 | 0.094024 | -0.598387 |
|  | C | 1.405319 | -1.118585 | -0.713106 |
|  | C | 0.045826 | -1.146291 | -0.461818 |
|  | C | -0.636432 | 0.040883 | -0.140732 |
|  | C | 0.06407 | 1.259782 | -0.088146 |
|  | C | 1.4229 | 1.293304 | -0.327029 |
|  | H | 3.137348 | 0.1479 | -0.98897 |
|  | H | 1.92856 | -2.02776 | -0.980632 |
|  | H | -0.484461 | -2.086968 | -0.55452 |
|  | H | -0.477734 | 2.169264 | 0.136439 |
|  | H | 1.963561 | 2.230393 | -0.286796 |
|  | C | -2.106683 | 0.082087 | 0.105127 |
|  | O | -2.73744 | -1.067577 | 0.429441 |
|  | H | -2.11791 | -1.794714 | 0.586459 |
|  | O | -2.762075 | 1.100649 | 0.02228 |
|  | O | 2.991991 | -0.207145 | 1.259033 |
|  | H | 2.155277 | -0.27674 | 1.746533 |
| TS22 | C | -2.345303 | -0.162264 | 0.382829 |
|  | C | -0.95167 | 1.842702 | -0.215775 |
|  | C | 0.284531 | 1.38234 | -0.313766 |
|  | C | 0.724624 | 0.007071 | -0.068162 |
|  | C | -0.110562 | -1.084346 | -0.129714 |
|  | C | -1.52733 | -1.086605 | -0.20963 |
|  | H | -2.029164 | 0.462252 | 1.206099 |
|  | H | -1.427365 | 2.798382 | -0.390148 |
|  | H | 1.042456 | 2.059416 | -0.723124 |
|  | H | 0.378864 | -2.04978 | -0.213734 |
|  | H | -2.001127 | -1.903871 | -0.74935 |
|  | C | 2.17401 | -0.283399 | 0.054322 |
|  | O | 3.004487 | 0.760845 | 0.318119 |
|  | H | 2.510047 | 1.574677 | 0.492048 |
|  | O | 2.676158 | -1.388785 | -0.048243 |
|  | O | -3.682856 | -0.115258 | 0.217175 |
|  | H | -3.945822 | -0.688492 | -0.518829 |
| FS1 | C | 1.98835 | 0.497272 | -0.000055 |
|  | C | 0.862161 | 1.319191 | -0.00001 |
|  | C | -0.41222 | 0.752237 | -0.000049 |
|  | C | -0.588133 | -0.642016 | 0.000011 |
|  | C | 0.557421 | -1.440186 | 0.000136 |
|  | C | 1.839448 | -0.887317 | -0.000007 |
|  | H | 2.976226 | 0.943811 | -0.000125 |
|  | H | 0.963852 | 2.399812 | 0.000046 |
|  | H | 0.436522 | -2.518701 | 0.000177 |
|  | H | 2.709687 | -1.533321 | -0.000092 |
|  | C | -1.972662 | -1.233117 | -0.000065 |
|  | H | -2.544369 | -0.912897 | 0.876659 |
|  | H | -2.542392 | -0.917273 | -0.879692 |
|  | H | -1.927095 | -2.323358 | 0.002691 |
|  | O | -1.552099 | 1.525798 | 0.000037 |
|  | H | -1.301831 | 2.45916 | 0.000268 |
| FS2 | C | 1.316986 | 1.023827 | 0.004483 |
|  | C | 1.214057 | -0.366943 | 0.000899 |
|  | C | -0.039392 | -0.984461 | -0.004793 |
|  | C | -1.207646 | -0.220203 | -0.00661 |
|  | C | -1.101197 | 1.178498 | -0.004918 |
|  | C | 0.150381 | 1.787727 | 0.001486 |
|  | H | 2.294653 | 1.494079 | 0.006735 |
|  | H | -2.000124 | 1.785506 | -0.010316 |
|  | H | 0.225166 | 2.869619 | 0.001391 |
|  | C | -2.561634 | -0.885776 | 0.004741 |
|  | H | -2.483088 | -1.953112 | -0.210208 |
|  | H | -3.043701 | -0.776787 | 0.981994 |
|  | H | -3.229809 | -0.434558 | -0.73375 |
|  | H | -0.088968 | -2.067956 | -0.010193 |
|  | O | 2.322342 | -1.184637 | 0.000056 |
|  | H | 3.117805 | -0.635705 | 0.002167 |
| FS3 | C | -1.437122 | -0.019939 | 0.001807 |
|  | C | -0.723343 | -1.220478 | -0.000631 |
|  | C | 0.667224 | -1.196372 | -0.004497 |
|  | C | 1.380539 | 0.010312 | -0.005639 |
|  | C | 0.644371 | 1.198409 | -0.00464 |
|  | C | -0.750808 | 1.193543 | -0.000601 |
|  | H | 1.16679 | 2.149475 | -0.008648 |
|  | H | -1.306696 | 2.125756 | -0.001673 |
|  | C | 2.889777 | 0.019899 | 0.005847 |
|  | H | 3.283968 | -0.31764 | 0.97039 |
|  | H | 3.300422 | -0.646139 | -0.758479 |
|  | H | 3.278617 | 1.023899 | -0.176214 |
|  | H | 1.209478 | -2.136589 | -0.00809 |
|  | H | -1.265878 | -2.158976 | -0.001814 |
|  | O | -2.814001 | -0.094083 | 0.003854 |
|  | H | -3.178511 | 0.800632 | 0.003819 |
| FS4 | C | -2.166609 | -0.599507 | -0.069394 |
|  | C | -1.885122 | 0.811545 | 0.18727 |
|  | C | -0.737135 | 1.518575 | 0.032663 |
|  | C | 0.617949 | 1.122566 | -0.303419 |
|  | C | 1.326407 | 0.034508 | 0.080891 |
|  | C | -1.419185 | -1.511367 | -0.666979 |
|  | H | -3.166025 | -0.922238 | 0.247703 |
|  | H | -2.754967 | 1.37762 | 0.505184 |
|  | H | -0.84771 | 2.594063 | 0.158645 |
|  | H | 1.194181 | 1.869234 | -0.8406 |
|  | H | -1.506382 | -2.557839 | -0.923317 |
|  | C | 2.755652 | -0.201876 | -0.285862 |
|  | H | 2.84976 | -1.137349 | -0.846963 |
|  | H | 3.149453 | 0.613327 | -0.891151 |
|  | H | 3.366923 | -0.303898 | 0.616441 |
|  | O | 0.856504 | -0.96614 | 0.88312 |
|  | H | -0.08901 | -0.850464 | 1.058085 |
| FS5.1 | C | -1.847715 | 1.436553 | 0.36012 |
|  | C | -2.353346 | 0.222126 | 0.237828 |
|  | C | -1.72172 | -0.997036 | -0.263454 |
|  | C | -0.406613 | -1.306894 | -0.372024 |
|  | C | 0.782761 | -0.510467 | -0.094689 |
|  | C | 0.856383 | 0.795558 | -0.432877 |
|  | H | -2.213737 | 2.392401 | 0.7115 |
|  | H | -3.387077 | 0.077938 | 0.575839 |
|  | H | -2.421443 | -1.79493 | -0.493939 |
|  | H | -0.191994 | -2.341603 | -0.635272 |
|  | C | 1.94477 | -1.235644 | 0.548106 |
|  | H | 1.837794 | -1.285743 | 1.637255 |
|  | H | 1.991206 | -2.265022 | 0.182632 |
|  | H | 2.909804 | -0.769307 | 0.329839 |
|  | O | 1.912264 | 1.633165 | -0.19669 |
|  | H | 2.557977 | 1.201891 | 0.380053 |
|  | H | 0.092241 | 1.293883 | -1.012449 |
| FS5.2 | C | 1.673329 | 0.603103 | -0.12156 |
|  | C | 0.785556 | 1.740965 | 0.01777 |
|  | C | -0.566708 | 1.856904 | 0.067125 |
|  | C | -1.628449 | 0.863424 | -0.037851 |
|  | C | -1.592341 | -0.458364 | 0.03963 |
|  | C | 1.438644 | -0.690674 | 0.16997 |
|  | H | 2.677983 | 0.844761 | -0.466821 |
|  | H | 1.318913 | 2.688265 | 0.038461 |
|  | H | -0.939297 | 2.874439 | 0.141234 |
|  | H | -2.620311 | 1.299581 | -0.220219 |
|  | H | 0.511525 | -1.058123 | 0.591679 |
|  | C | -2.526454 | -1.586423 | -0.054393 |
|  | H | -3.55092 | -1.224461 | -0.230385 |
|  | H | -2.26232 | -2.260066 | -0.876165 |
|  | H | -2.534966 | -2.182754 | 0.864227 |
|  | O | 2.342741 | -1.701029 | 0.007021 |
|  | H | 3.155997 | -1.347025 | -0.382325 |
| FS6.1 | C | -1.858559 | -0.344589 | -0.18859 |
|  | C | -1.46628 | 0.893622 | 0.170183 |
|  | C | -0.193835 | 1.538648 | -0.086397 |
|  | C | 1.068095 | 1.055715 | -0.208187 |
|  | C | 1.589758 | -0.307053 | -0.060072 |
|  | C | 0.882652 | -1.421663 | -0.170532 |
|  | H | -2.211401 | 1.515853 | 0.664692 |
|  | H | -0.268449 | 2.623261 | -0.126091 |
|  | H | 1.841867 | 1.803911 | -0.360267 |
|  | H | 1.090641 | -2.482112 | -0.111278 |
|  | C | 3.080689 | -0.388876 | 0.259757 |
|  | H | 3.415992 | -1.422123 | 0.356576 |
|  | H | 3.659609 | 0.091065 | -0.535822 |
|  | H | 3.302368 | 0.138371 | 1.192858 |
|  | O | -3.075709 | -0.890323 | 0.094414 |
|  | H | -3.583965 | -0.282241 | 0.651547 |
|  | H | -1.256103 | -1.018215 | -0.784505 |
| FS6.2 | C | 1.535624 | -0.175304 | 0.264727 |
|  | C | 0.159113 | 2.355444 | -0.039195 |
|  | C | -1.06957 | 1.87227 | -0.091331 |
|  | C | -1.562563 | 0.510196 | 0.090966 |
|  | C | -0.911513 | -0.679953 | -0.001399 |
|  | C | 0.512922 | -0.892115 | -0.235313 |
|  | H | 0.611171 | 3.331001 | -0.159877 |
|  | H | -1.863933 | 2.593824 | -0.322484 |
|  | H | -2.638059 | 0.454383 | 0.231163 |
|  | H | 0.768295 | -1.776137 | -0.819892 |
|  | C | -1.719489 | -1.958452 | 0.056669 |
|  | H | -1.573414 | -2.54542 | -0.857096 |
|  | H | -1.395334 | -2.59106 | 0.890011 |
|  | H | -2.786247 | -1.758763 | 0.169992 |
|  | O | 2.856638 | -0.424424 | 0.030456 |
|  | H | 2.943527 | -1.149678 | -0.605749 |
|  | H | 1.413745 | 0.644728 | 0.959544 |
| FS7 | C | 1.028345 | -0.993316 | -0.213362 |
|  | C | 1.834071 | -0.033857 | 0.280595 |
|  | C | -0.198574 | 2.004497 | -0.007663 |
|  | C | -1.251996 | 1.23341 | -0.197556 |
|  | C | -1.404185 | -0.214374 | 0.002452 |
|  | C | -0.404995 | -1.134649 | -0.041086 |
|  | H | 1.515487 | -1.802519 | -0.757892 |
|  | H | 0.017223 | 3.059027 | -0.118737 |
|  | H | -2.161756 | 1.724561 | -0.567369 |
|  | H | -0.73377 | -2.172146 | -0.019976 |
|  | C | -2.835931 | -0.672715 | 0.163534 |
|  | H | -2.907232 | -1.761156 | 0.19805 |
|  | H | -3.277092 | -0.275438 | 1.084745 |
|  | H | -3.458753 | -0.310538 | -0.662133 |
|  | O | 3.182107 | 0.038571 | 0.06625 |
|  | H | 3.444349 | -0.651684 | -0.560335 |
|  | H | 1.504291 | 0.747346 | 0.952159 |
| FS8 | C | 1.904567 | 1.182853 | -0.002302 |
|  | C | 2.567238 | -0.045316 | -0.000798 |
|  | C | 1.837909 | -1.234355 | 0.002007 |
|  | C | 0.447074 | -1.200459 | 0.002676 |
|  | C | -0.221035 | 0.031055 | 0.000914 |
|  | C | 0.515133 | 1.222285 | -0.001159 |
|  | H | 2.470797 | 2.106674 | -0.004528 |
|  | H | 3.650799 | -0.075544 | -0.001821 |
|  | H | 2.352981 | -2.187622 | 0.003547 |
|  | H | -0.118014 | -2.122984 | 0.004715 |
|  | H | -0.008332 | 2.16994 | -0.002381 |
|  | C | -1.70309 | 0.118598 | 0.000558 |
|  | O | -2.333789 | 1.159628 | 0.004429 |
|  | O | -2.31169 | -1.085128 | -0.00509 |
|  | H | -3.271173 | -0.934428 | -0.005621 |
| FS9 | C | 2.303775 | 0.267639 | -0.391512 |
|  | C | 1.391574 | 1.222616 | -0.689769 |
|  | C | 0.125989 | 1.516709 | -0.027997 |
|  | C | -0.949422 | 0.728738 | 0.175331 |
|  | C | 1.234719 | -1.192543 | 1.345909 |
|  | C | 2.275787 | -0.721838 | 0.689402 |
|  | H | 3.225543 | 0.284809 | -0.964058 |
|  | H | 1.689273 | 1.954765 | -1.437082 |
|  | H | 0.018277 | 2.539984 | 0.328343 |
|  | H | 1.062725 | -1.898308 | 2.146985 |
|  | H | 3.264779 | -1.097792 | 0.975855 |
|  | C | -1.250272 | -0.611126 | -0.427251 |
|  | O | -0.424683 | -1.149442 | -1.32803 |
|  | H | 0.363918 | -0.596173 | -1.479513 |
|  | O | -2.28913 | -1.17692 | -0.140499 |
|  | O | -2.001147 | 1.204527 | 0.907843 |
|  | H | -2.69773 | 0.526224 | 0.870272 |
| FS10.1 | C | -2.117699 | -1.375731 | -0.061168 |
|  | C | -2.311387 | 0.034935 | -0.261419 |
|  | C | -1.579054 | 1.030215 | 0.293434 |
|  | C | 0.846107 | -0.381529 | 0.003072 |
|  | C | 0.391228 | -1.63611 | 0.059416 |
|  | C | -0.973049 | -2.09506 | 0.130143 |
|  | H | -3.025792 | -1.966804 | -0.152993 |
|  | H | -3.170919 | 0.325485 | -0.862963 |
|  | H | 1.14261 | -2.430089 | -0.004019 |
|  | H | -1.087357 | -3.167787 | 0.239363 |
|  | C | 2.173474 | 0.185549 | -0.043464 |
|  | O | 2.247618 | 1.539005 | -0.033063 |
|  | H | 1.353738 | 1.915895 | -0.018487 |
|  | O | 3.206129 | -0.464404 | -0.089419 |
|  | O | -1.76945 | 2.350648 | 0.077407 |
|  | H | -2.441774 | 2.484125 | -0.608332 |
|  | H | -0.822614 | 0.863564 | 1.047959 |
| FS10.2 | C | -2.856538 | -0.186767 | 0.455323 |
|  | C | -2.658182 | 1.07187 | 0.786268 |
|  | C | 0.131849 | 1.144708 | -0.691126 |
|  | C | 0.306725 | -0.141339 | -0.294181 |
|  | C | -0.712925 | -1.16335 | -0.571846 |
|  | C | -2.031128 | -1.151337 | -0.296363 |
|  | H | -3.792527 | -0.632958 | 0.803842 |
|  | H | -1.993628 | 1.911504 | 0.666441 |
|  | H | -0.781846 | 1.436125 | -1.193612 |
|  | H | -0.321266 | -2.072493 | -1.019865 |
|  | H | -2.583958 | -2.036837 | -0.596646 |
|  | C | 1.604648 | -0.646906 | 0.207122 |
|  | O | 2.336876 | 0.189088 | 1.009514 |
|  | H | 1.76235 | 0.807991 | 1.486567 |
|  | O | 2.0699 | -1.730549 | -0.069486 |
|  | O | 0.988403 | 2.179833 | -0.624081 |
|  | H | 1.842758 | 1.91841 | -0.245479 |
| FS11.1 | C | -2.463267 | 0.675165 | -0.490587 |
|  | C | -2.767759 | -0.53887 | 0.005057 |
|  | C | 0.141303 | -1.159459 | -0.611698 |
|  | C | 0.687036 | -0.017108 | -0.214319 |
|  | C | -0.020015 | 1.24982 | -0.015076 |
|  | C | -1.332424 | 1.531812 | -0.184459 |
|  | H | -3.230901 | 1.110897 | -1.123334 |
|  | H | -3.671421 | -1.055414 | -0.301061 |
|  | H | 0.419847 | -2.187474 | -0.792426 |
|  | H | 0.631064 | 2.079768 | 0.233036 |
|  | H | -1.583232 | 2.589631 | -0.13359 |
|  | C | 2.168909 | 0.040098 | 0.088914 |
|  | O | 2.951186 | -0.95842 | -0.367072 |
|  | H | 2.451701 | -1.570212 | -0.928322 |
|  | O | 2.67823 | 0.932397 | 0.732017 |
|  | O | -2.015576 | -1.17636 | 0.940796 |
|  | H | -2.410472 | -2.036884 | 1.132773 |
| FS11.2 | C | 2.561351 | -1.027531 | -0.471662 |
|  | C | 1.221997 | 1.690145 | -0.180601 |
|  | C | 0.19195 | 1.00641 | -0.710675 |
|  | C | -0.443906 | -0.202997 | -0.171286 |
|  | C | 0.163127 | -1.377202 | 0.139456 |
|  | C | 1.553506 | -1.766505 | -0.043147 |
|  | H | 3.616952 | -1.171256 | -0.657127 |
|  | H | 1.601985 | 2.584943 | -0.659259 |
|  | H | -0.247467 | 1.444 | -1.603003 |
|  | H | -0.478645 | -2.153533 | 0.540768 |
|  | H | 1.764446 | -2.814107 | 0.197 |
|  | C | -1.926884 | -0.163701 | 0.031383 |
|  | O | -2.511038 | 1.053911 | -0.050183 |
|  | H | -1.836778 | 1.742932 | -0.170847 |
|  | O | -2.627201 | -1.129726 | 0.26495 |
|  | O | 1.901773 | 1.423443 | 0.959979 |
|  | H | 1.544393 | 0.634284 | 1.393691 |
| FS12 | C | 2.49616 | -0.080374 | -0.241048 |
|  | C | 0.620781 | 2.146467 | 0.001451 |
|  | C | -0.458465 | 1.450556 | 0.295433 |
|  | C | -0.732193 | 0.026746 | 0.065612 |
|  | C | 0.179964 | -0.993986 | 0.05433 |
|  | C | 1.608039 | -1.024596 | 0.152557 |
|  | H | 2.21484 | 0.827643 | -0.760127 |
|  | H | 0.963447 | 3.161706 | 0.145543 |
|  | H | -1.245039 | 1.997055 | 0.831559 |
|  | H | -0.271642 | -1.981165 | 0.030574 |
|  | H | 2.024764 | -1.960323 | 0.520027 |
|  | C | -2.155678 | -0.398831 | -0.038503 |
|  | O | -3.089628 | 0.571407 | -0.222551 |
|  | H | -2.68286 | 1.440086 | -0.354514 |
|  | O | -2.548432 | -1.550473 | 0.017715 |
|  | O | 3.834728 | -0.17818 | -0.111169 |
|  | H | 4.0715 | -0.982919 | 0.375984 |

# Table S5: Imaginary Frequency Data of Each Transition State Calculated at the B3LYP/6-311++G(d,p) Level of Theory.

| Species | Imaginary Frequency | Infrared |
| --- | --- | --- |
| TS1 | -995.86 | 1694.8052 |
| TS2 | -998.56 | 1806.0029 |
| TS3 | -1075.74 | 1744.7063 |
| 7S4 | -245.37 | 9.651 |
| TS5 | -467.25 | 13.5683 |
| T56 | -197.91 | 11.4586 |
| TS7.1 | -220.57 | 6.0302 |
| 7S7.2 | -252.01 | 8.0523 |
| 7S8 | -257.79 | 10.2317 |
| TS9.1 | -237.38 | 1.3761 |
| TS9.2 | -259.95 | 2.4562 |
| TS10 | -233.71 | 7.9743 |
| TS11 | -233.89 | 3.6918 |
| TS12 | -165.62 | 50.9152 |
| TS13.1 | -150.43 | 10.096 |
| TS13.2 | -841.66 | 2994.5851 |
| TS14.1 | -1740.88 | 1289.7606 |
| TS14.2 | -1224.51 | 703.2761 |
| TS15 | -272.05 | 6.4686 |
| TS16 | -127.88 | 1.6388 |
| TS17 | -258.77 | 21.6736 |
| TS18.1 | -127.37 | 4.4138 |
| TS18.2 | -230.81 | 10.8457 |
| TS19 | -305.6 | 13.8516 |
| TS20.1 | -396.81 | 13.1429 |
| TS20.2 | -429.35 | 14.0807 |
| TS21 | -273.22 | 23.4653 |
| TS22 | -166.91 | 3.0639 |

# Table S6: Free Energy Data of Various Reaction Species at 303K-340K (in atomic units, a.u.).

| T | IS | ·OH | TS1 | TS2 | TS8 | TS10 | TS12 | FS8 | TS15 | TS17 |
| --- | --- | --- | --- | --- | --- | --- | --- | --- | --- | --- |
| 303 | -271.489 | -75.7501814 | -347.2273815 | -347.2261923 | -347.2299197 | -347.2315383 | -347.2275311 | -420.7810435 | -496.5152 | -496.5158 |
| 305 | -271.4891 | -75.750318 | -347.227667 | -347.226478 | -347.230203 | -347.231821 | -347.227814 | -420.781309 | -496.5155 | -496.5161 |
| 310 | -271.4897 | -75.750659 | -347.228385 | -347.227194 | -347.230912 | -347.232531 | -347.228524 | -420.781974 | -496.5163 | -496.5168 |
| 315 | -271.4903 | -75.751001 | -347.229107 | -347.227914 | -347.231626 | -347.233245 | -347.229238 | -420.782643 | -496.517 | -496.5176 |
| 320 | -271.4909 | -75.751343 | -347.229833 | -347.228638 | -347.232344 | -347.233963 | -347.229956 | -420.783315 | -496.5177 | -496.5183 |
| 325 | -271.4916 | -75.751687 | -347.230563 | -347.229367 | -347.233066 | -347.234685 | -347.230679 | -420.783992 | -496.5185 | -496.5191 |
| 330 | -271.4922 | -75.752032 | -347.231298 | -347.2301 | -347.233791 | -347.235411 | -347.231405 | -420.784672 | -496.5192 | -496.5199 |
| 335 | -271.4928 | -75.752377 | -347.232036 | -347.230837 | -347.234521 | -347.236141 | -347.232135 | -420.785356 | -496.52 | -496.5206 |
| 340 | -271.4935 | -75.752723 | -347.232779 | -347.231578 | -347.235255 | -347.236875 | -347.23287 | -420.786044 | -496.5207 | -496.5214 |
